# The Genetic Landscape of Dystrophin Mutations in Italy: A Nationwide Study

**DOI:** 10.3389/fgene.2020.00131

**Published:** 2020-03-03

**Authors:** Marcella Neri, Rachele Rossi, Cecilia Trabanelli, Antonio Mauro, Rita Selvatici, Maria Sofia Falzarano, Noemi Spedicato, Alice Margutti, Paola Rimessi, Fernanda Fortunato, Marina Fabris, Francesca Gualandi, Giacomo Comi, Silvana Tedeschi, Manuela Seia, Chiara Fiorillo, Monica Traverso, Claudio Bruno, Emiliano Giardina, Maria Rosaria Piemontese, Giuseppe Merla, Milena Cau, Monica Marica, Carmela Scuderi, Eugenia Borgione, Alessandra Tessa, Guia Astrea, Filippo Maria Santorelli, Luciano Merlini, Marina Mora, Pia Bernasconi, Sara Gibertini, Valeria Sansone, Tiziana Mongini, Angela Berardinelli, Antonella Pini, Rocco Liguori, Massimiliano Filosto, Sonia Messina, Gianluca Vita, Antonio Toscano, Giuseppe Vita, Marika Pane, Serenella Servidei, Elena Pegoraro, Luca Bello, Lorena Travaglini, Enrico Bertini, Adele D'Amico, Manuela Ergoli, Luisa Politano, Annalaura Torella, Vincenzo Nigro, Eugenio Mercuri, Alessandra Ferlini

**Affiliations:** ^1^Unit of Medical Genetics, Department of Medical Sciences, University of Ferrara, Ferrara, Italy; ^2^Neuroscience Section, Department of Pathophysiology and Transplantation, Dino Ferrari Center, University of Milan, Milan, Italy; ^3^Laboratory of Medical Genetics, IRCCS Ca’ Granda Ospedale Maggiore Policlinico, Milan, Italy; ^4^Paediatric Neurology and Muscular Diseases Unit, University of Genoa and G. Gaslini Institute, Genoa, Italy; ^5^Center of Translational and Experimental Myology, IRCCS Gaslini, Genova, Italy; ^6^Molecular Genetics Laboratory UILDM, Santa Lucia Foundation, Rome, Italy; ^7^Division of Medical Genetics, IRCCS Casa Sollievo della Sofferenza, Foggia, Italy; ^8^Laboratory of Genetics and Genomics, Department of Medical Science and Public Health, University of Cagliari, Cagliari, Italy; ^9^Clinica Pediatrica e Malattie Rare, Brotzu, Cagliari, Italy; ^10^Unit of Neuromuscular Diseases, Oasi Research Institute-IRCCS, Troina, Italy; ^11^Department of Molecular Medicine, IRCCS Fondazione Stella Maris, Pisa, Italy; ^12^Department of Biomedical and Neuromotor Sciences, University of Bologna, Bologna, Italy; ^13^Neuromuscular Diseases and Neuroimmunology Unit, Fondazione IRCCS Istituto Neurologico Carlo Besta, Milan, Italy; ^14^Neurorehabilitation Unit, Department Biomedical Sciences for Health, University of Milan, Milan, Italy; ^15^Neuromuscular Center, AOU Città della Salute e della Scienza, University of Turin, Turin, Italy; ^16^Child Neurology and Psychiatry Unit, “Casimiro Mondino” Foundation, Pavia, Italy; ^17^Child Neurology Unit, IRCCS Istituto delle Scienze Neurologiche, Bologna, Italy; ^18^Department of Biomedical and Neuro Motor Sciences, University of Bologna, Bologna, Italy; ^19^Department of Clinical and Experimental Medicine, University of Messina and Nemo Sud Clinical Center, Messina, Italy; ^20^Centro Clinico Nemo, Policlinico A. Gemelli, Fondazione Policlinico Universitario A. Gemelli IRCCS, Rome, Italy; ^21^UOC Neurofisiopatologia, Fondazione Policlinico Universitario A. Gemelli IRCCS, Institute of Neurology, Catholic University of Sacred Heart, Rome, Italy; ^22^Department of Neurosciences, University of Padua, Padua, Italy; ^23^Unit of Neuromuscular and Neurodegenerative Disorders, Department of Neurosciences, Bambino Gesu Children's Research Hospital IRCCS, Rome, Italy; ^24^Cardiomiology and Medical Genetics, University of Campania “Luigi Vanvitelli, Naples, Italy; ^25^Department of Precision Medicine, University of Campania “Luigi Vanvitelli, Naples, Italy; ^26^Pediatric Neurology, Catholic University, Rome, Italy; ^27^Dubowitz Neuromuscular Unit, Institute of Child Health, University College London, London, United Kingdom

**Keywords:** dystrophin, muscular dystrophy, nationwide study, exon skipping therapy, read-through therapy

## Abstract

Dystrophinopathies are inherited diseases caused by mutations in the dystrophin (*DMD*) gene for which testing is mandatory for genetic diagnosis, reproductive choices and eligibility for personalized trials. We genotyped the *DMD* gene in our Italian cohort of 1902 patients (BMD n = 740, 39%; DMD n =1162, 61%) within a nationwide study involving 11 diagnostic centers in a 10-year window (2008–2017). In DMD patients, we found deletions in 57%, duplications in 11% and small mutations in 32%. In BMD, we found deletions in 78%, duplications in 9% and small mutations in 13%. In BMD, there are a higher number of deletions, and small mutations are more frequent than duplications. Among small mutations that are generally frequent in both phenotypes, 44% of DMD and 36% of BMD are nonsense, thus, eligible for stop codon read-through therapy; 63% of all out-of-frame deletions are eligible for single exon skipping. Patients were also assigned to Italian regions and showed interesting regional differences in mutation distribution. The full genetic characterization in this large, nationwide cohort has allowed us to draw several correlations between DMD/BMD genotype landscapes and mutation frequency, mutation types, mutation locations along the gene, exon/intron architecture, and relevant protein domain, with effects on population genetic characteristics and new personalized therapies.

## Introduction

Dystrophin gene *(DMD* OMIM *300377*)* mutations account for different allelic conditions: Duchenne muscular dystrophy (DMD, OMIM *310200), which is the most common form of muscular dystrophy in childhood, occurring in 1 in 3,500 to 5,000 male births, and Becker muscular dystrophy (BMD, OMIM *300376), a milder form, with an incidence of 1 in 20,000 live male births (Mah, 2016).

Allelic dystrophin mutations can also give rise to isolated cardiac involvement, or X-linked dilated cardiomyopathy (XLDC, OMIM*302045) (Neri et al., 2012).

*DMD* gene mutations cause reduction (BMD) or complete absence (DMD) of the dystrophin protein (DYS), which expression is vital for a series of striated muscles and brain functions (Muntoni et al., 2003). The phenotype of DMD/BMD is characterized by a delayed achievement of motor milestones and by an elevated level of the muscle isoform, creatine phosphokinase (M-CK), present also at birth, which rises as a direct consequence of muscle damage.

The dystrophin gene (*DMD*), with its 79 constitutive exons, and at least other 7 alternatively-used exons, is the largest known human gene, spanning 2.2 Mb of genomic DNA (Muntoni et al., 2003). Due to its enormous size, the gene mutation rate is high, and 1 out of 3 are *de novo* mutations. Gross rearrangements (i.e., deletions and duplications) account for the majority of cases (75%) and the remaining mutations are small mutations (25%) or rarely deep intronic CNVs/small mutations (see many reports as cited in Leiden online variation database https://databases.lovd.nl/shared/genes/DMD) (Artsma-Rus et al., 2006; White and den Dunnen, 2006).

Mutations can occur everywhere in the gene, but few mutational hot spots are known: deletions cluster preferentially between exons 45 to 55 and duplications in the region of exons 2 to 10 (Ankala et al., 2012; White et al., 2006). An additional hot spot occurs at the 5′ end of the gene involving intron 7. It has been previously reported that deletions are mostly maternally inherited, whereas duplications originate from the grand paternal germline, therefore presenting more frequently as familial cases and with a higher recurrence risk (Hu et al., 1990; White et al., 2006). Very rare *DMD* complex rearrangements are described, such as intronic CNVs causing new cryptic splice sites which induce pseudoexon shuffling into the transcript or exon orientation inversion leading to exon skipping. These atypical genomic configurations escape the routine DNA-based diagnostic procedures (either MLPA or sequencing) and could be identified only by RNA studies (Ferlini et al., 2013).

RNA studies are also of relevance in cases of mutations with uncertain pathogenic meaning, like missense, synonymous or even nonsense mutations, which may occur in exonic splicing enhancers/silencers, therefore affecting splicing choices. The study of the RNA profile in skeletal muscle/myogenic cells might therefore be compulsory in some cases (Falzarano et al., 2015).

The genotype-phenotype correlation generally follows the frame rule: mutations (all types) that disrupt the translational open reading frame cause almost complete protein absence and lead to the severe DMD phenotype, whereas mutations maintaining the reading frame allow a shorter protein production and are associated with the milder BMD clinical phenotype (Monaco et al., 1988).

Exceptions to the rules have however been described and hold approximately for 10% of DMD/BMD cases (Bladen et al., 2015). Alternative splicing, spontaneous exon skipping or alternative translation initiation mechanisms play a role in these exceptions, but still, the explanation of many of these events remains unknown (Gualandi et al., 2009; Wein et al., 2014; Tuffery-Giraud et al., 2017).

Understanding the type and frequency of patient-specific mutations is mandatory for genetic diagnosis and counseling and for establishing the eligibility for mutation-specific clinical trials that, in the last years, increasingly target specific groups of mutations, such as deletions amenable to skipping individual exons or nonsense mutations (https://clinicaltrials.gov/).

Molecular analysis of the huge *DMD* gene is routinely performed in many laboratories worldwide using different techniques and specific guidelines have been defined (Abbs et al., 2010).

We present here the results of genetic analysis in a large Italian cohort of 1902 genotyped patients diagnosed in a temporal window of 10 years in 11 Italian diagnostic centers belonging to the EURO-NMD (https://ern-euro-nmd.eu) European Reference Center (as Health Care Providers or HCPs). This represents the first report on Italian dystrophinopathies and the largest cohort of proband male patients with independent mutations reported until now. Such a genetic *DMD* mapping drives many considerations and reflection on dystrophinopathy diagnosis, prevention, and therapy.

## Materials and Methods

### Ethical Aspects

This is a collaborative effort including the 11 reference centers (see the full list in **Table 1**) providing dystrophin genetic analysis in Italy. Ethical consent was collected in each center as part of the routine diagnostic procedures for *DMD* genetic diagnosis. Data analyses in this paper were carried out based on the EU project BIO-NMD Ethical Approval N. 9/2005.

**Table 1:** Centers of the Italian network and genotypic data of the patients. All the variants identified by the centers were submitted to the LOVD database (www.lovd.nl).

	PATIENTS ENROLLED	DMD	BMD	DELETIONS ALL	DELETIONS DMD	DELETIONS BMD	DUPLICATIONS ALL	DUPLICATIONS DMD	DUPLICATIONS BMD	NONSENSE ALL	NONSENSE DMD	NONSENSE BMD	MISSENSE ALL	MISSENSE DMD	MISSENSE BMD	FRAMESHIFTING ALL	FRAMESHIFTING DMD	FRAMESHIFTING BMD	SPLICING (CANONICAL SITES) ALL	SPLICING (CANONICAL SITES) DMD	SPLICING (CANONICAL SITES) BMD	SPLICING CONSENSUS SITES ALL	SPLICING CONSENSUS SITES DMD	SPLICING CONSENSUS SITES BMD	SYNONIMOUS ALL	SYNONIMOUS DMD	SYNONIMOUS BMD
**ROMA Bambino Gesu Children's Research Hospital IRCCS**	160	109	51	144	97	47	13	9	4	2	2	0	1	1	0	1	1	0	0	0	0	0	0	0	0	0	0
**ROMA UILDM Sez Lazio/ Fondazione Santa Lucia**	236	119	117	216	100	116	6	5	1	4	4	0	0	0	0	9	9	0	0	0	0	1	1	0	0	0	0
**GENOVA Paediatric Neurology and Muscular Diseases Unit, University of Genoa and G. Gaslini Institute**	140	78	62	113	62	51	14	5	9	6	4	2	1	1	0	4	4	0	2	2	0	0	0	0	0	0	0
**PISA IRCCS Fondazione Stella Maris Department of Molecular Medicine**	74	26	48	63	20	43	5	4	1	2	0	2	0	0	0	1	1	0	2	1	1	0	0	0	0	0	0
**MILANO Laboratory of Medical Genetics Foundation IRCCS Ca' Granda Ospedale Maggiore Policlinico**	25	13	12	21	11	10	4	2	2	0	0	0	0	0	0	0	0	0	0	0	0	0	0	0	0	0	0
**MILANO Neurology Unit, IRCCS Ca' Granda Ospedale Maggiore Policlinico**	146	79	67	69	24	45	17	14	3	31	26	5	5	2	3	13	9	4	7	4	3	2	1	1	0	0	0
**NAPOLI Università degli Studi della Campania L. Vanvitelli**	276	165	111	151	67	84	29	14	15	44	39	5	4	3	1	31	30	1	13	10	3	3	3	0	0	0	0
**Division of Medical Genetics, IRCCS Casa Sollievo della Sofferenza, S. GIOVANNI ROTONDO**	37	22	15	35	20	15	2	2	0	0	0	0	0	0	0	0	0	0	0	0	0	0	0	0	0	0	0
**University of Cagliari, P.O. Pediatrico Microcitemico “A. Cao”, CAGLIARI**	25	14	11	13	6	7	7	4	3	3	1	2	3	1	2	1	1	0	0	0	0	1	1	0	0	0	0
**Unit of Neuromuscular Diseases, Oasi Research Institute-IRCCS, TROINA (En)**	13	7	6	13	7	6	0	0	0	0	0	0	0	0	0	0	0	0	0	0	0	0	0	0	0	0	0
**FERRARA Unit of Medical Genetics, University of Ferrara**	769	530	239	404	253	151	93	64	29	108	90	18	24	6	18	79	72	7	43	36	7	14	12	2	4	1	3
**TOTAL**	1902	1162	740	1242	667	575	190	123	67	200	166	34	38	14	24	139	127	12	67	53	14	21	18	3	4	1	3

This study was performed based on the RARER (Emilia Romagna Region Grant, Area 1A) project and evaluated and approved by the Local Ethical Committee of the University Hospital of Ferrara, Italy (ethical approval n. 139–2012, date of approval December, 20, 2012). Written informed consent was obtained from both patients and parents either for study participation and for publication aims.

### Enrolled Patients

Enrollment criteria for the study were the following: i) male patients with a clinical diagnosis of dystrophinopathy (DMD or BMD) with or without muscle biopsy; ii) *DMD* mutation (any type) identified; iii) genetic diagnoses made between the 1^st^ of January 2008 and the 31^st^ of December 2017. Of the 1902 index patients who fulfilled the inclusion criteria in the 11 centers, 1162 were clinically classified as DMD and 740 as BMD (see **Table 1**).

Categorization of patients as DMD or BMD was based on clinical criteria (Birnkrant et al., 2018)

### Laboratory Methods

The genetic diagnosis was performed by deletion and duplication detection (Multiple Ligation Probe Amplification, LOG-PCR, multiplex PCR) in the majority of cases as the first approach and then by sequencing analysis (Sanger method, Multiplicom Next Generation Sequencing, and Motorplex).

Genomic DNA was extracted from EDTA-preserved whole blood using either automated or manual methods following manufacturer's instructions

MLPA assay was performed using the P034/P035 *DMD* Kit (MRC Holland). Amplified products were analyzed using an ABI 3100 analyzer (Applied Biosystems, Foster City, CA) with GeneMarker software version 1.5.1 (Soft Genetics, State College, PA). Peak heights were normalized, and a deletion or duplication was identified when the normalized peak ratio value was 0 or 2 for male patients. (Schouten et al., 2002; Schwartz and Dunø, 2004; Vengalil et al., 2017). When a single-exon deletion or duplication was observed, Sanger sequencing was used for verification.

The LOG-PCR (Trimarco et al., 2008) is a new tool for complete screening of *DMD* exons. This method uses only 4 quantitative multiplex PCRs, which are run under the same reaction and cycling conditions, and can also be useful for assessing carrier status when the mutation is known. It detects deletions and duplications and provides a proof of principle for higher-throughput multiplex PCR methods.

In the Ospedale Maggiore Policlinico Milano laboratory, MLPA was used as the first screening for deletions or as confirmation of positive results by home-made quantitative multiplex-PCR.

For detection of small mutations, analysis was carried out by Sanger: PCR primers (provided by request) were designed by Vector NTI Advance (Informax Frederick, Maryland USA) analysis software to amplify all coding exons and flanking intronic sequences of *DMD* (RefSeq NM_004006.2). PCR products generated using Taq DNA polymerase (Roche, Indianapolis, IN) were sequenced in both forward and reverse orientations using the BigDye Terminator Cycle Sequencing kit (version 1.1) and analyzed on a sequencer. Patient sequence data were aligned for comparison with corresponding wild-type sequence.

In the Ferrara center for Next Generation Sequencing (NGS), the library preparation was done with the *DMD* MASTR™ assay (Multiplicom, Niel Belgium) according to the manufacturer's instructions. All 79 exons were sequenced in 118 amplicons spanning 280 to 400 bp and with a minimal coverage per allele of 50×. Sequencing was done on MySeq (Alame et al., 2016).

In the Genoa, Roma UILDM, and Cagliari centers for NGS sequencing, the amplification of coding regions and flanked intronic regions (10 bp) of *DMD* gene was performed by Ampliseq method (reference sequence NM_004006.2); sequencing was performed by IonTorrent method on PGM platform. The data analysis was then performed by CLC Genomics Workbench e Ion Reporter software. All mutations were validated by Sanger sequencing.

Motorplex is a targeting NGS workflow created for diagnosis of genetic myopathies (Savarese et al., 2014). More than 95% of targeted nucleotides were read at a 100× depth and a 500× depth was obtained for 80% of these. MotorPlex may discover low-allelic fraction variants in single samples, such as in somatic mosaicisms. The MotorPlex is likewise the cheapest genetic test ever presented that is able to screen 93 complex conditions at the cost of a few PCR reactions.

### Data Analysis

The results were analyzed by looking for frequency distribution of deletions, duplications, and small mutations in both DMD and BMD patients.

Within deletions and duplications, we analyzed those occurring in single or multiple exons, the last either being in-frame or out-of-frame. Deletion or duplication sites were thus considered depending on the adjacent intron length and on the involvement of regions encoding crucial proteins domains. Some evolutionary aspects of the giant introns composing the *DMD* gene were also highlighted and discussed. Within the small mutation group, we explored the frequency of small mutations occurring in in-frame and out-of-frame exons.

We categorized our patients based on their place of birth and consequently assigned them to specific Italian regional areas. North, Center, South (including Sicily) and Sardinia were considered separately because of their known different genetic profiles and ethnic characteristics (Capocasa et al., 2014).

Finally, since during the last decade there have been an increasing number of new therapeutic approaches targeting specific groups of mutations with some drugs that already received the approval of regulatory authorities in the USA and/or in Europe, we also have identified the frequency of mutations amenable for personalized therapies.

More specifically, we identified the frequency of nonsense mutations eligible for stop codon readthrough therapy, and the frequency of the groups of deletions amenable for skipping of exon 44, 45, 51, and 53, which are/were in clinical trials.

## Results

**Figure 1:**
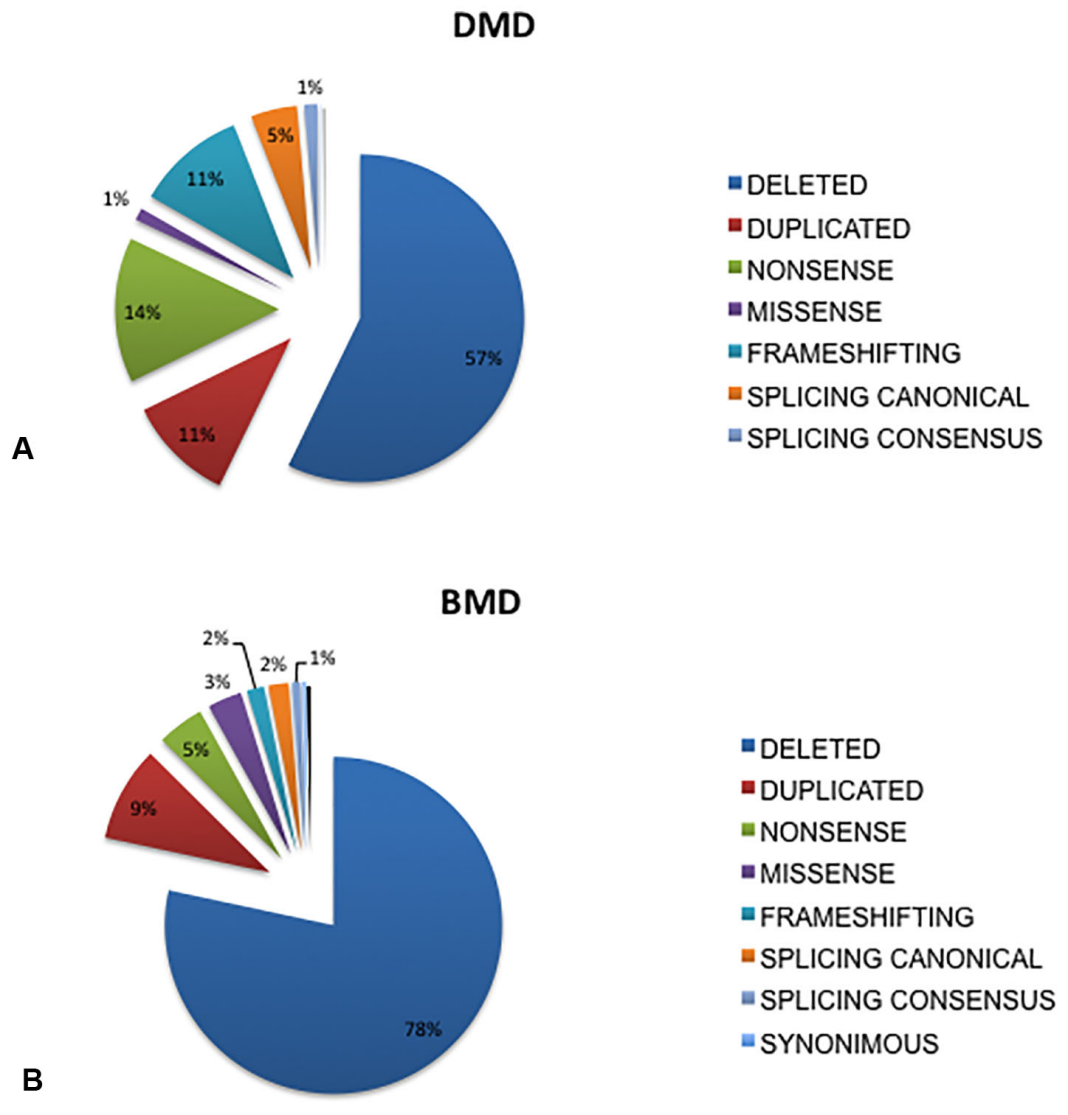
Overview of mutations distribution in DMD and BMD patients from Italy. Deletions were the most frequent occurring mutations, accounting for 57% of mutation types in the DMD patients **(A)** and in 78% in the BMD patients **(B)**, duplications occurred at a similar rate in both DMD (11%) and BMD patients (9%), small mutations occurred in 32% of DMD and 13% of BMD patients. Among all mutation types, nonsense are the most frequently occurring small changes both in DMD (14%) and in BMD (5%), followed by frameshifting (DMD 11%, BMD 2%), and splicing canonical sites (DMD 5%, BMD 2 %), missense (DMD 1%, BMD 3%). Mutations occurring in splicing consensus sequences are 1% both in DMD and in BMD.

A *DMD* mutation was identified in 1902 patients. **Table 1** shows the genotypic data of the Italian cohort. **Figure 1** shows the mutation type distribution in DMD and BMD Italian patients.

Deletions were the most frequently occurring mutations, accounting for 57% of mutation types in the DMD patients (**Figure 1A**) and 78% in the BMD patients (**Figure 1B**). Duplications occurred at a similar rate in both DMD (11%) and BMD patients (9%). Small mutations occurred in 32% of DMD and 13% of BMD patients.

Among all small mutations, nonsense were the most frequent in both DMD (14%) and in BMD (5%), followed by frameshifting (DMD 11%, BMD 2%), splicing canonical sites (DMD 5%, BMD 2 %), and missense (DMD 1%, BMD 3%). Mutations occurring in splicing consensus sequences were 1% in both DMD and in BMD. Not unexpectedly, missense mutations were more frequently found in BMDs, which also show less nonsense changes than DMD. A few (4) proven pathogenic synonymous mutations were found (**Supplementary Table 1**).

### Deletions

The most common single exon deletion in DMD was exon 45, while the most common multiple exon deletion was 45 to 52. Deletions were very heterogeneous and non-randomly distributed, occurring in the two known hot spots at the 5′ and 3′ end of the gene (**Supplementary Figure 1**).

Single exon deletion was never found in 29 in-frame exons (namely exons 3, 4, 9, 15, 16, 23, 25, 26, 28, 29, 32, 33, 34, 35, 36, 37, 38, 39, 41,42, 49, 60, 64, 71, 72, 73, 74, 75, 79) both in DMD and BMD. A few (12) out-of-frame exons (7, 11, 20, 59, 62, 65, 66, 67, 69, 70, 76, 77) were also never the site of single exon deletions.

### Duplications

The most common single exon duplication occurred in exon 2 while the most common multiple exon duplication was in exon 3 to 7. The major 5′ breakpoints occurred within intron M1 or 2 for single duplications and within intron 2 (5′ end) and intron 7 (3′ end) for the multiple exon duplications (**Supplementary Figure 1**). Since genomic architecture of duplications cannot be identified neither by MLPA or CGH testing, the frame rule is not unambiguously applicable to these rearrangements to predict, for instance, possible self-reframing capability.

Similarly to deletions, n = 35 in-frame exons were never the site of isolated duplications (neither in DMD nor in BMD), as in-frame exons 4, 9, 10, 13, 14, 15, 23, 24, 25, 26, 27, 28, 30, 31, 32, 33, 34, 35, 36, 37, 38, 39, 40, 42, 47, 48, 49, 60, 64, 71, 72, 73, 74, 75, 79 and n = 26 out-of-frame exons 6,7,17,19, 20, 43, 46,51, 52, 53, 56, 57, 58, 59, 61, 62, 63, 65, 66, 67, 68, 69, 70, 76, 77, 78 also were never singly duplicated.

There were a few in-frame exons which were only singly deleted (exons 3, 16, 29, 41) or singly duplicated (exons 10, 13, 14, 24, 27, 30, 31, 40, 47, 48) and some out-of-frame exons only singly deleted (11) or singly duplicated (exons 6, 17,19, 43, 46, 51, 52, 53, 56, 57, 58, 61, 63, 68, 78). In general, 41 *DMD* exons are never the site of isolated deletions, whilst 61 *DMD* exons are never the site of isolated duplications.

This means that single-exon rearrangements affect approximately less than a half of the 79 *DMD* exons and conversely, the majority of large rearrangements involve multiple exons.

### Small Mutations

The rate of small mutations in DMDs was 32%. Among these, nonsense was the most frequently occurring class of mutation at 44%, followed by frameshifting (34%), splicing canonical sites (14%), splicing consensus (4%), and the rare missense (4%).

BMDs show 13% of small mutations (**Figure 1**).

Distribution of the small changes was along the whole gene, without hot spots, with a rather homogenous spectrum of change types; among small mutations in BMD nonsense were the majority (36%) followed by missense (26%), splicing canonical sites (15%), splicing consensus sequence (7%), frameshifting (13%) and missense (3%). Compared to other reports, missense variations were very frequent in the Italian BMDs. In BMDs, also the small changes distribution was random without preferred sites.

**Figure 2:**
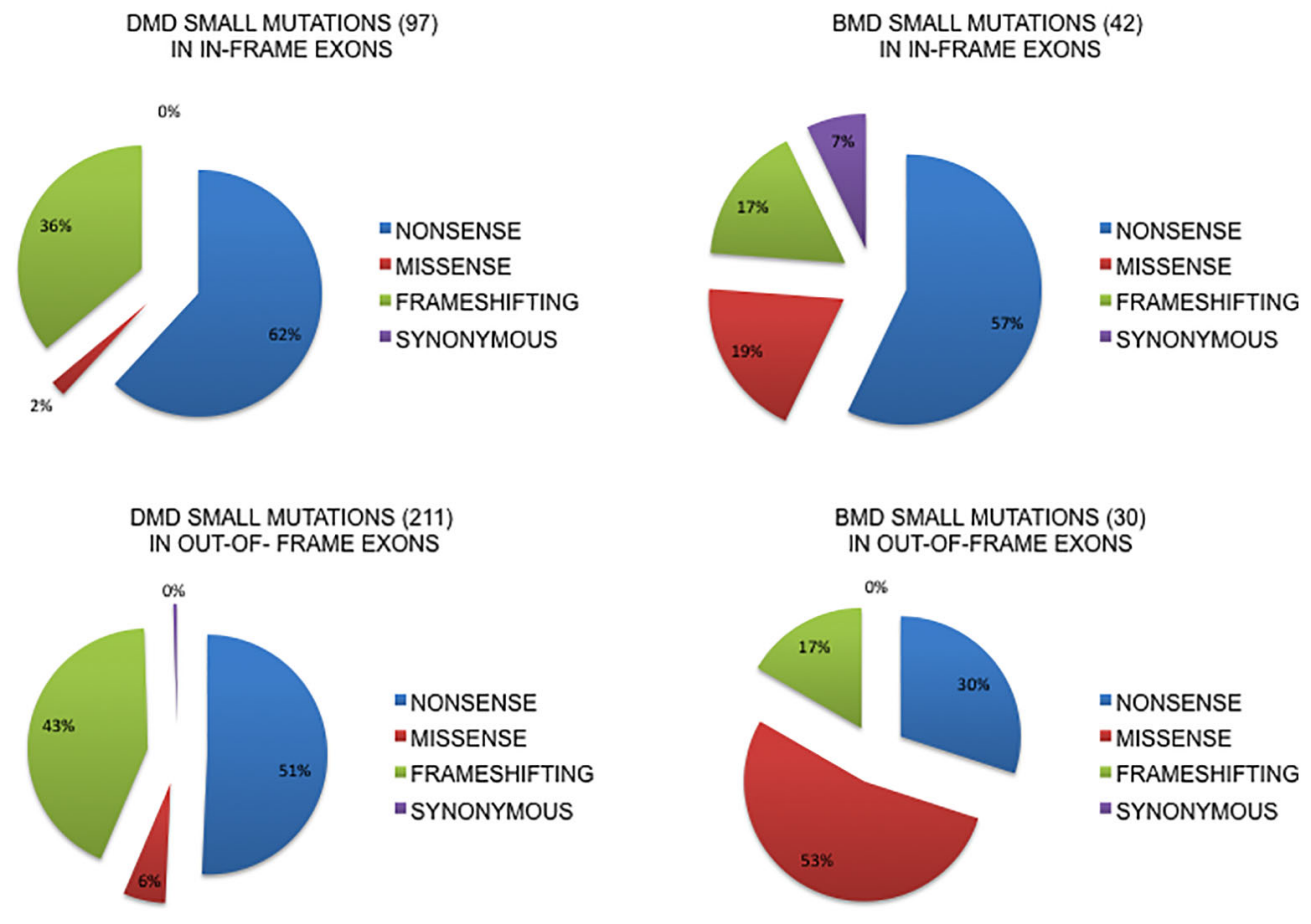
Small mutations distribution in in-frame or out-of-frame exons in DMD and BMD patients. The majority of small changes in DMD (68.5%, n=211) locates in out-of-frame exons, while only 31.4% (n=97) locates in in-frame exons. Nonsense mutations occur in 62% in-frame exons compared to 51% in out-of-frame exons, frameshifting changes are more frequent in out-of-frame exons and the missense are 2% in in-frame vs 6% out-of-frame. In BMDs, small changes are prevalent in in-frame exons (58.3%, n= 42) compared to out-of-frame (41.6%, n=30) exons. Nonsense mutations occur much more frequently, almost double, in in-frame exons (57% vs 30%), frameshifting changes are equally distributed (17%) and missense mutations are more represented in out-of-frame exons (53% vs 19%).

**Figure 2** shows the localization of small changes (all) in in-frame or out-of-frame exons both in DMD and BMD patients. The majority of small changes in DMD (68.5%, n = 211) locates in out-of-frame exons, while only 31.4% (n = 97) locates in in-frame exons, thus predicting a general low self-reframing capability. This type of change is not homogenously distributed since nonsense mutations occur in 62% of in-frame exons compared to 51% in out-of-frame exons, frameshifting changes are more frequent in out-of-frame exons (43% vs 36% in-frame) and the missense are 2% in in-frame vs 6% out-of-frame.

In BMDs, small changes are prevalent in in-frame exons (58.3%, n = 42) compared to out-of-frame (41.6%, n = 30) exons. Nonsense mutations occur much more frequently, almost double, in in-frame exons (57% vs 30%), frameshifting changes are equally distributed (17%) and missense mutations are more represented in out-of-frame exons (53% vs 19%). We also analyzed the splicing mutations involving the canonical splice sites, both donor and acceptor, and for this specific group of mutations it is difficult to predict the exact consequences on splicing patterns and therefore on framing of the exons involved. It is well known from literature that the effect of mutations at the canonical splice site usually lead to single exon skipping (upstream or downstream exon) but if there is a strong cryptic site in the neighborhood it can be used instead (Habara et al., 2009; Abramowicz and Gos, 2019) (see the complete list of splicing mutations in DMD and BMD in **Supplementary Table 2**).

### Regional *DMD* Mutation Distribution

**Figure 3:**
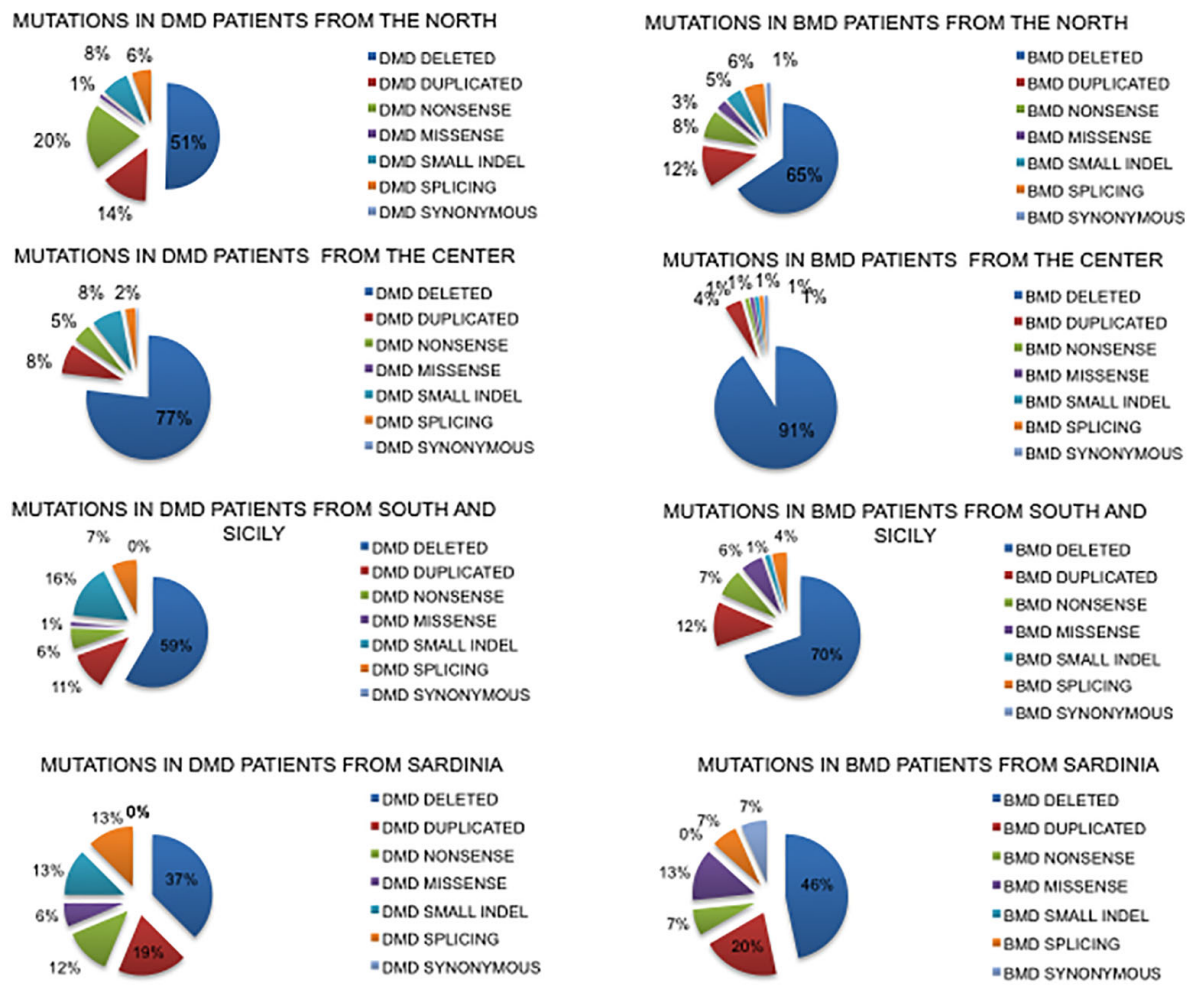
Geographical distribution of mutations based on the place of birth of DMD and BMD patients. Nonsense mutations account for only 6% in the South compared to 20% in the North, with the counterpart of frameshifting changes being 8% in the North and 16% in the South. Sardinian patients show a very different mutation spectrum.

Deletions had a similar distribution in the North and South regions (51%–59% respectively in DMDs and 65%–70% in BMDs) while in Central Italy they were more frequent both in DMD (77%) and in BMD (91%) (**Figure 3**).

Duplications had a similar distribution in North and South regions (14%–11% DMD and 12% BMD while in Central Italy they were less frequent (8% in DMD and 4% in BMD).

Small mutations were differently distributed in DMDs. Nonsense mutations account for only 6% in the South region compared to the 20% in the North, with frameshifting around 8% in the North and 16% in the South. In Central Italy, small mutations were much lower.

Data for Sardinia may suffer due to the low number of patients diagnosed (so far 32), with small mutations very frequent (42% in DMD and 34% in BMD) compared to large rearrangements.

### Mutations Amenable to Mutation-Specific Therapies

In our cohort, the percentage of DMD patients eligible for stop codon reversion therapy was 14.4%. We also explored the stop codon type (TGA, TAA, TAG) occurring in DMD. All three nonsense codons were present in the mutational scenario, however regional differences occurred (**Supplementary Figure 2**). In the North region, the three stop codons were equally present in mutated DMDs, while in the South and in Sicily half (49%) of DMD patients carried the TGA stop codon. In the Center, the TAA stop codon was poorly represented (13%). In Sardinian patients, the TAG stop codon was not present.

The trend in BMD patients in the North, in the South and in Sicily was very similar to DMDs, while in the Center only TAA stop codons occurred. Sardinian BMD patients showed only TGA stop codons.

In our Italian cohort, the percentage of DMD patients eligible for exon skipping was 63% of the patients with out-of-frame deletions; among these, 17.8% was eligible for exon 53 skipping, 17% for exon 51, 11.4% for exon 44, 16.6% for exon 45. There were some regional differences (**Supplementary Figure 3**). The North and Center regions showed very similar percentages of skipping amenability, with the exon 53 skipping being the most frequently skippable exon, and the South and Sicily had a different pattern, with exon 45 being the predominant skippable exon. In Sardinian patients, only those eligible for exon 44 skipping were found.

**Figure 4:**
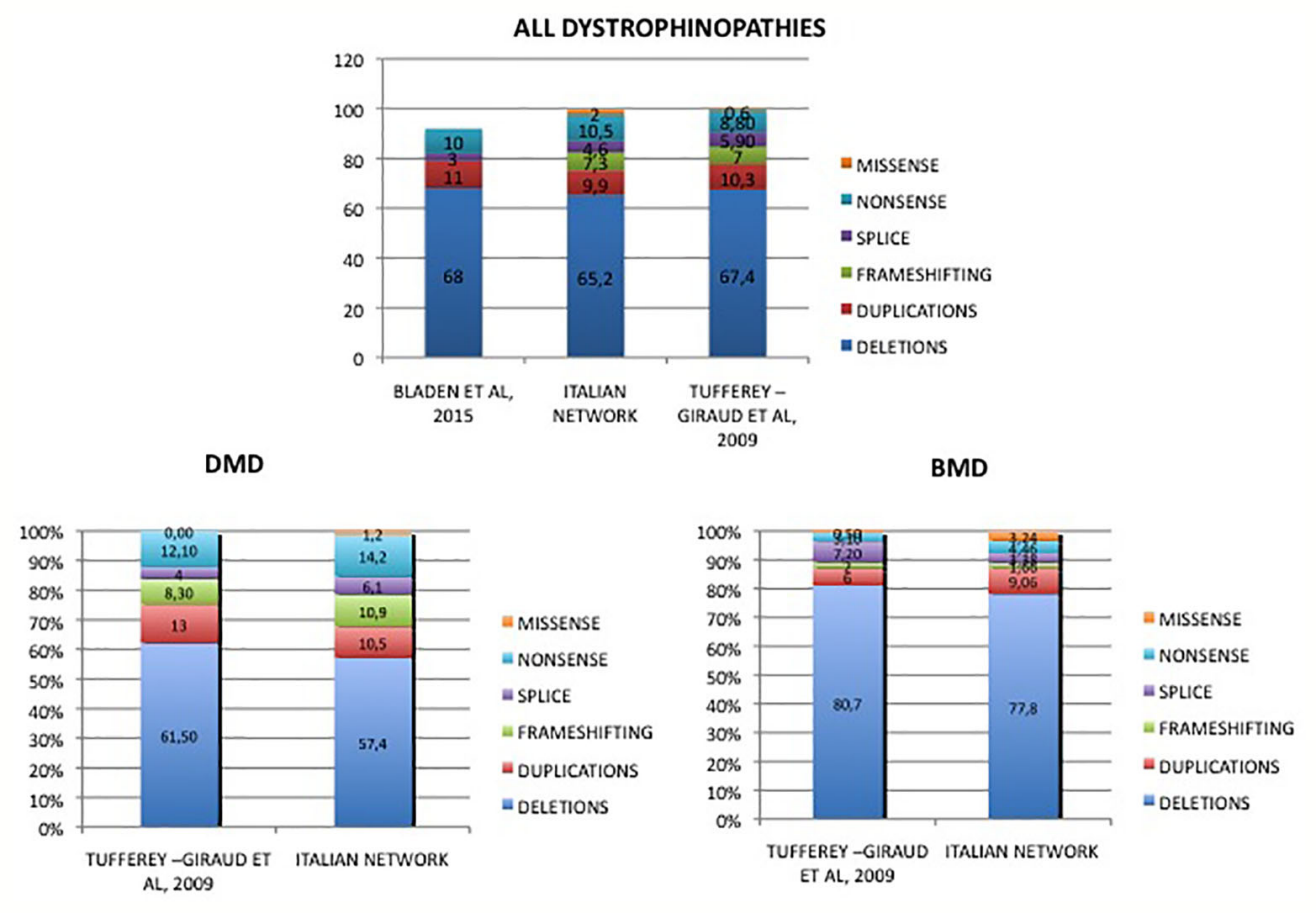
Mutations distribution in DMD and BMD patients from Italy compared to literature data. Overview of genotype data of our cohort in comparison to previously reported other nationwide studies. Tuffery-Giraud et al. (2009). Hum. Mutat. 30, 934-45; Bladen et al. (2015). Hum. Mutat. 36, 395-402.

**Table 2** and **Figure 4** show an overview of genotype data of our cohort compared to the two previously reported nationwide studies.

**Table 2:** Mutation distribution in Duchenne and Becker patients of our cohort compared to previously published cohorts.

Phenotype	DMD Mutations	Bladen et al., 2015	Tufferey-Giraud et al., 2009	Italian network	Mean value
**ALL DYSTROPHINOPATHIES** **(MALES, CARRIERS)**		7149	2084	1902	
	LARGE REARRANGMENTS	80%	77%	75,2%	77,4%
	SMALL	20%	22,3%	24,7%	22,3%
				
	DELETIONS	68%	67.4%	65,2 %	66,8%
	DUPLICATIONS	11%	10.3%	9,9 %	10,4%
	FRAMESHIFTING	(7%)	7.0%	7,3%	7,15%
	SPLICING	3%	5,9%	4,6%	4,5%
	NONSENSE	10%	8,8%	10,5%	9,7%
	MISSENSE	(1%)	0,6	2%	1,2%
					
**DUCHENNE**			1315	1162	
	LARGE REARRANGMENTS		74%	67,9%	70,9%
	SMALL		26%	32%	29%
				
	DELETIONS		61,5%	57,4%	59,4%
	DUPLICATIONS		13%	10,5%	11,7%
	INDELS		8,3%	10,9%	9,6%
	SPLICE		4%	6,1%	5%
	NONSENSE		12,1%	14,2%	13,15%
	MISSENSE		0.9%	1,2%	1%
				
	Most single exon deletion		45 (7, 4%)	45 (4%)	
	Most multiexon deletion		45–50 (5,8%)	45–52 (3,2%)	
	Most single exon duplication		2 (9,8%)	2 (13%)	
	Most multiple exon duplication		3–7 (5.1%)	3–7 (4,7%)	
**BECKER**			560	740	
	LARGE REARRANGEMENTS		86,8%	86,86	86,8%
	SMALL		13,2%	13,14	13,1%
				
	DELETIONS		80,7	77,8	79,2%
	DUPLICATIONS		6%	9,06	7,5%
	INDELS		2%	1,66	1,8%
	SPLICING		7,2%	3,38	5,29
	NONSENSE		3,1%	4,46	3,78
	MISSENSE		0,5%	3,24	1,87%
				
	Most single exon deletion		48 (3,5%)	48 (4,7%)	
	Most multiexon deletion		45–47 (29%)	45–47 (5.9%)	
	Most single exon duplication		None	3 (1%) 16 (1%)	
	Most multiple exon duplication		2–7 (2.8%)	3–16 (2.6%) 19–41 (2.6%)	

## Discussion

Rare Diseases (RDs) represent a major challenge worldwide, with many initiatives devoted to achieving an appropriate genetic and clinical diagnosis launched in many countries (http://www.udninternational.org). Early and accurate genetic diagnosis is recommended by all guidelines and is now considered compulsory for mutation identification, allowing for prevention and family planning, and applies to all dystrophinopathy mutation groups (Koeks et al., 2017).

In addition, the emergence of new therapeutic and often personalized approaches in DMD has further highlighted the need for an early genetic definition in order to identify eligible patients. Many of the new drugs are mutation-specific or gene-specific, e.g. nonsense mutations for stop-codon read-through or frame-shift deletions for exon skipping and gene therapy, which also requires a genotype definition (Cirak et al., 2011; McDonald et al., 2017).

In order to define the mutational landscape in Italian DMD and BMD patients, we retrospectively collected (January 2008-December 2017) genetic data from 11 diagnostic Italian Reference Centers, identifying a total of 1902 patients carrying a *DMD* mutation. Mutation detection methods were rather homogeneous, invariably based on CNV detection predominantly by MLPA (9/11 centers), followed by sequencing (11/11 centers) via NGS techniques (either gene-specific or based on gene panels); all centers also used Sanger sequencing for mutation validation or in patients diagnosed before the availability of NGS approaches.

### *DMD* Deletions and Duplications: Frequency, Distribution, Topography

In our Italian cohort, mutation-type frequency substantially overlaps those already published in other patient cohorts, with some peculiarities. DMD patients have 57% of deletions and 11% of duplications, which is slightly lower compared to other European studies reporting deletions of more than 60% and of duplications of more than 10% (Tuffery-Giraud et al., 2009; Bladen et al., 2015).

In a Chinese study, the percentage of rearrangements in DMD patients is similar to ours, with 60% of deletions and 9.6% of duplications (Guo et al., 2015) while in an Indian cohort (Polavarapu et al., 2019) there was a higher prevalence of deletions (80%) and only a small percentage of duplications (5%) with a complete absence of the most common exon 2 duplication. In a very large sample population of United Dystrophinopathy Project, Flanigan et al. (2009) reported a 43% percentage of deletions and 11% of duplications in all the dystrophinopathy patients (including DMD, BMD, and intermediate phenotypes); the low percentage of deletion compared to the one reported in literature is discussed as due to a selection bias. This heterogeneous mutation scenario supports the existence of population or ethnic differences, which have to be taken into account when planning country-specific genetic screening or even therapeutic approaches.

Among all dystrophinopathies, the most frequent multiple exons deletion is 45 to 52, while it was 45 to 50 in the French population (Tuffery-Giraud et al., 2009). These two major deletion events therefore share the 5′ breakpoint in intron 44 but not the 3′ breakpoint, which lies in intron 52 for the Italian and in intron 50 for the French patients. Again, these two introns (50, 45782 bp and 52, 50044 bp, quite similar in length) might have genetic differences in the two populations. Remarkably, blast analysis of the two introns revealed an anti-oriented 87% homology in 861 bps. Four (T)^n^(A)^n^, motif-rich regions can be seen, one of these being an exact TTTAAA in intron 50, but not in intron 52 (TTAAAA) (**Supplementary Table 3**). This motif is known to lead to micro-homology recombination events. It might be hypothesized that population-specific SNP(s) within this motif in intron 52 may modify its rearrangement propensity.

Distribution of deletions in our patient cohort confirms the two well-known hot spots at the 5′ end (surrounding exon 3–7) and the broader region at the 3′ end of the gene involving exons 45 to 54. Since the more frequently deleted exon is 45 (either as single exon deletion or in multiple deleted exons interval), it means the vast majority of *DMD* deletions have the 5′ breakpoint in the huge intron 44 (248401 bp), apart from very rare cases with the breakpoint within the exon 45 itself. This is quite consistent with the Tuffery (Tuffery-Giraud et al., 2009) and Bladen (Bladen et al., 2015) reports. Introns 1 (whose genomic annotation is promoter-specific depending on the brain, muscle, and Purkinje full-length isoform driving sequences), 7 and 44 are the largest *DMD* introns, a phenomenon known as intron gigantism, which is quite frequent in vertebrates (Pozzoli et al., 2003). Although it is unknown why intron gigantism occurs, large introns are thought to function as “providers” of regulatory regions or new exons (exon shuffling), which might confer evolutionary advantages to genes from one side, but cause high recombination rates via non homologous recombination, from the other (Rogozin et al., 2005). It is also known that out-of-frame exons are separated by significant longer introns compared to in-frame exons, as clearly evident in the DYS rod-domain, a large region from exon 23 to exon 42 where all exons are in-frame and separated by shorter introns (average of 22.400 bp, ranging from 309 bp of intron 35 to 31823 bp of intron 41) (Pozzoli et al., 2002). Indeed the 1, 7, and 44 giant introns are just downstream of out-of-frame exons (1, 7, and 44), which are (not surprisingly) the *DMD* deletion hot spots. These three introns are also very rich in short tandem repeats (especially TTTAAA or (T)n(A)^n^), which are known to cause aberrant firing of the replication fork, as frequent mechanisms responsible for micro-homology related recombination events (Marey et al., 2016). We would like to underline that these three exons are the upstream exons of regions encoding extremely important DYS domains: the actin binding domain (exon 1, although possibly dispensable, as suggested by Wein et al., 2014), the Hinge 1 (exon 7) and the nNOS binding domain, involved in DYS signaling functions (Rybakova et al., 2000). Also Hinge2 and Hinge3 domains are encoded by out-of-frame exons such as 17 (intron length 35.892 bp) and 50 (intron length 45.764 bp). It is quite likely that these large introns had relevant impact on the evolution of the human *DMD* gene leading to the acquisition of new functions. Recently, new *DMD* microgenes designed for gene therapy and inclusive of Hinge 1, Hinge 2 and nNOS domains resulted more functional in mice (Ramos et al., 2019). Based on this knowledge, it might be important to carefully define deletion intervals by annotating intronic breakpoints to better understand how a different genomic architecture or even locus rearrangements may impact on RNA transcription, protein translation machinery, and clinical outcome. This might also be relevant in optimizing gene therapy approaches. More generally, introns deserve more studies in order to investigate and define their functions in the *DMD* gene context. The availability of WGS will certainly facilitate this task in the very near future.

Analyzing our mutation scenario, both in DMD and BMD, 29 in-frame exons (exons 3, 4, 9, 15, 16, 23, 25, 26, 28, 29, 32, 33, 34, 35, 36, 37, 38, 39, 41,42, 49, 60, 64, 71, 72, 73, 74, 75, 79) are never the site of single exon deletion. This is interesting data, never having been investigated in other reports. Possible interpretations are that these exons do not belong to regions susceptible to mutational events or, conversely, that single deletions occurring in these in-frame exons might not be pathogenic, thus causing asymptomatic or very mildly-affected individuals only. These cases may have escaped genetic definition since diagnostic tests are generally only performed on symptomatic patients. This second hypothesis seems to be supported by the consistency of this finding in BMD as well, where we would expect in-frame deletions leading to milder phenotypes. Interestingly (but not surprisingly), the majority (85%) of “never singly deleted” exons belong to the large rod-shaped protein domain (R, where 3′-R6 to 5′-R16 or spectrin-like tandem repeats) encoded by the long sequel of in-frame exons 23 to 42. Among the “never singly deleted” in-frame exons, exons 3, 16, 29, and 41 are also never singly duplicated, supporting their possible dispensability for the *DMD* gene essential functions.

Combining this last consideration with previous observations, it might be that the central DYS rod-shaped domain is not entirely necessary for the protein anchorage function, however it may influence other protein functions not directly related to the structural DYS role at the sarcolemma, but may also be more relevant for other non-muscle compartments. Indeed, a deep clinical characterization of BMD patients carrying different deletion types might help to better understand the DYS protein domains preservation consequences and residual functions both in striated muscles and other tissues, like for instance, the brain.

Duplications occur at similar frequencies in DMD (11%) and BMD (9%) as described in other reports, and they are distributed anywhere along the gene, though 42% involve the 5′ hot spot between introns 1 and 9, according to literature data (Aartsma-Rus et al., 2017); the most common single exon duplication is exon 2, in agreement with previous literature data (Wein et al., 2014).

Single exon duplication hot spots have major 5′ breakpoints within Dp427 muscle isoform intron 1 and intron 2, while multiple exon duplications do have the major sites within intron 2 (upstream) and intron 7 (downstream). Therefore, both deletions and duplications recognize their breakpoints in giant introns 1 and 7, but not intron 44, where duplication breakpoints rarely locate. This is also an interesting finding since mechanisms underlining deletion and duplication events are different indeed. Deletions seem to be frequently related to micro-homology in breakpoint regions, mainly due to the presence of short tandem repeats, interspersed repeat elements which can predispose to deletion events (Esposito et al., 2017) or (as already mentioned above) due to an aberrant firing replication fork. Duplications are known to arise from either homologous (such as Alu-Alu) recombination or non-homologous recombination, which is mediated by topoisomerases activity. Some papers describe, in detail, *DMD* duplications mainly as intra-chromosomal and propose that unequal sister chromatid exchange might be the major mechanism (Worton, 1992). It is known that segmental duplication was found to be associated with rapid primate gene evolution and chromosomal rearrangement (Samonte and Eichler, 2002). Notably, duplications (but not deletions) occurred at a similar rate in DMD (11%) and in BMD (9%) patients, in all cohorts, supporting a common underlining mutational mechanism, irrespective of the phenotype.

Finally, deletions and duplications might be primed by preexisting small *indels* or point mutations, which may elicit the rearrangement events via attempts to correct preexisting mutations, as we speculated above for intron 50 and 52 (Oshima et al., 2009). In this context, full intron sequencing, via WGS strategy, will have a tremendous impact on the understanding of *DMD* rearrangement mechanisms, and the occurrence of the many known synonymous changes (SNPs) in the gene may find novel avenues for interpreting their functional meaning.

BMD patients show an 87% of gross rearrangements (deletion and duplication), strongly suggesting that they must be first approached by CNV detection during the diagnostic flowchart and not by sequencing. Indeed, a false perception of “negative results” may come from a negative NGS test done in BMD patients, a fact that may delay or even hamper the clinical diagnosis. Moreover, the majority of large *DMD* rearrangements involve multiple exons, implying that deletion/duplication detection should be done by exploring all exons, and therefore using fully accurate techniques.

### Small Mutations Frequency, Distribution, and Types

The rate of small mutations in the Italian DMD cohort is 32%, higher than those reported by other papers. Differently from gross rearrangements, small mutations are randomly distributed along all exons without hot spots, and the majority of them are “private,” contributing to the tremendous allelic heterogeneity of dystrophinopathies.

Small mutation-type frequency is a bit more articulated compared to other reported data. In both DMD and BMD, nonsense changes are 14% and 5% respectively, thus the most frequent small variant-type occurring in Italian patients. As a peculiarity, Italian BMD have 3% of missense mutations, which is a rather high percentage, compared to DMD or other published cohorts. Indeed, the pathogenicity of missense mutations (generally, and not only in DMD/BMD) needs robust evidence like, for instance, being previously reported in databases or confirmed by functional studies or robust *in silico* prediction, following the pathogenicity scale recommendations of the American College of Medical Genetics and the European Society of Human Genetics (Richards et al., 2015). The large number of BMDs diagnosed in our cohort may justify the high number of missense mutations identified. Indeed, the BMD phenotype may need to be clinically differentially diagnosed with many LGMDs (Vissing, 2016), or alternatively it might be very mild, even escaping a clinical diagnosis and consequently the *DMD* genetic testing.

It has been hypothesized that small changes occurring in in-frame exons may cause milder phenotypes via spontaneous exon skipping of the mutated exon, which may elide (to some extent and in a quantitative manner) the mutation consequence. Indeed, the majority of small changes (all types) in our DMD Italian patients localizes in out-of-frame exons (68.5%, N = 211 vs 31.4% N = 97 in in-frame exons), therefore predicting a general low self-reframing capability. Specifically, according to the self-framing capability rule, nonsense variants occur in 62% of in-frame exons compared to 51% in out-of-frame exons, frameshifting changes are more frequent in out-of-frame exons (43% vs 36% in-frame ) and missense are 2% in in-frame vs 6% out-of-frame.

In BMDs, small changes are slightly prevalent in in-frame exons (58%, n = 42) compared to out-of-frame (42%, n = 30) exons. Interestingly, nonsense mutations occur much more frequently in in-frame exons (57% vs 30%), frameshifting changes are equally distributed (both being 17%) and missense mutations are significantly more represented in out-of-frame exons (53% vs 19%).

The “small-in-frame” trend for small mutations in BMD partially applies for nonsense changes, which might sometimes account for the milder phenotype of BMD carrying a single premature stop codon in an in-frame exon that can be favorably skipped. The in-frame exon might indeed be able to self-reframe dystrophin by endogenous skipping, leading to an ameliorated phenotype. Nevertheless, the “small-in-frame” trend does not meaningfully apply for missense mutations, as hypothesized by some authors, and therefore it is not expected to play a crucial role in determining milder phenotypes. Of course, it is possible that self-correcting exon skipping may involve more than one exon and in this case prediction of phenotype effect based on small mutations location is not possible (Aartsma-Rus et al., 2016). This is even more true for splicing mutations for which it is not possible to predict “a priori” the precise effect on skipping and therefore on self-reframing and phenotypic outcome.

It should be also mentioned that single nucleotide changes might alter the complex process of exon recognition by the splicing machinery, via weakening the exonic splicing enhancers (ESEs), for which identification in vitro studies are needed (Tuffery-Giraud et al., 2017).

### Regional Data

The high genetic heterogeneity of the Italian population is well known and widely reported (Chiang et al., 2018). *DMD* mutation-type distribution in the North and South regions is overall similar, with some remarkable differences (**Figure 3**).

Nonsense mutations account for only 6% in the South compared to the 20% in the North, with the counterpart of frameshifting changes being 8% in the North and 16% in the South. This is a new finding in the *DMD* gene. It is very well known that mutation types vary greatly in different ethnic groups, as documented for many autosomal recessive diseases (Rohlfs et al., 2011). Indeed, the heterogeneous mutation scenario, for instance in cystic fibrosis (worldwide), has led to mutation-specific diagnostic approaches in some regions and has addressed diagnostic flowcharts with implications in mutation-driven newborn screening procedures (Bosch et al., 2017). Mutagenic mechanisms underlining point mutations and frameshifting changes are also different, since single nucleotide substitutions are more often caused by DNA replication errors or proofreading failure Ankala et al., 2012), while frameshifting changes are due to imperfect inverted repeats which cause a deletion or a tandem duplication of the flanking nucleotides (Todorova and Danieli, 1997). Therefore, it is not surprising that genetic background may play a role in small mutation occurrence diversity.

Sardinian patients show a very different mutation spectrum. Deletions are only 35% in DMD and 46% in BMD, and small mutations are very frequent (42% in DMD and 34% in BMD). Although data may suffer with the low number of residential patients diagnosed (so far 31), the concordant percentage between DMD and BMD suggests that the trend may reflect the ethnic peculiarity of Sardinia (Chiang et al., 2018).

We have also analyzed the stop codon types (TGA, TAA, TAG) in all nonsense mutations identified in our patients (N = 200) (**Supplementary Figure 2**). The three codons are almost equally represented in the mutation spectrum (national average). However, regional distribution showed that in the North and Center regions, this trend is maintained with TAG slightly more frequent (38% North, 47% Center). In the South and Sicily, the TGA codon is the most frequent nonsense occurring mutation (49%).

Sardinian DMD show only TGA and TAA nonsense codons, while in BMD only the TGA codon (100%) was found. This very peculiar behavior supports, again, a role of ethnic background in mutagenesis and *DMD* gene mutation occurrence and distribution (Tian et al., 2008), which would really deserve further studies. Alternatively (but possibly concomitantly), the known phenomenon of codon usage bias, meaning that some synonymous/stop codons are negatively selected through evolution, may play a role in the different regional nonsense mutation types occurrence since, obviously, nonsense mutation types depend on the native codon in the permutated sequence (Hanson and Coller, 2018).

### Therapeutic Implications

In our Italian cohort the percentage of DMD patients eligible for stop codon reversion therapy (such as Translarna) is 14.4%.

The other personalized therapeutic approach is based on antisense oligoribonucleotides (AONs) aimed at inducing favorable, dystrophin-reframing, exon skipping (Aartsma-Rus et al., 2017). Different backboned AONs are currently in use, either in clinical trials (see in ClinicalTrials.gov) or even designated as orphan drugs such as Eteplirsen, or Exondys51, which induces the *DMD* exon 51 skipping in amenable patients (Syed, 2016). Other molecules that induce the skipping of exon 44, 45, and 53 are also currently in clinical trials.

In our Italian cohort, the percentage of DMD patients eligible for exon skipping (all 4 amenable exons) is 63% among all out-of-frame deletions, with (in percentage order) 17.8% eligible for exon 53, 17% for exon 51, 16.6% for exon 45 and 11.4% for exon 44 skipping. Similarly to the nonsense mutation type, skipping eligibility also shows some regional differences. The most frequently skippable exon is exon 53 in the North and Center regions and exon 45 in the South and in Sicily. In Sardinian patients, only exon 44 skipping is applicable in the currently diagnosed DMD patients.

## Conclusions

Our large and nationwide *DMD* gene mutation report (so far the first published in Italy) revealed that mutation types as gross categories (rearrangements and small mutations) overlap in terms of frequency with those of other similar reports, but mutation-type frequency differences are also present, even at a regional level, where ethnicity may play a role in mutation diversity. We analyzed deletions and duplication intervals in detail and suggest that the genomic rearrangement mechanisms underlining the two events are different. This complexity suggests the need for a finer genetic characterization in DMD and BMD patients (full genome sequencing might be in the near future), especially to better address drug design and understand the outcome of gene specific therapies.

From the deletion data it has emerged that some exons might be dispensable and some “asymptomatic” cases may carry these “invisible” *DMD* deletions. A systematic search for incidental findings occurring in these exons might be essential, as it could reveal the true frequency of these “healthy” events. Knowing if some *DMD* exons are definitively dispensable for a DYS protein full function would have a tremendous impact on gene therapy design.

Small mutations are very frequent in the DMD Italian cohort patients, but rarer in BMD. This is also an important finding, addressing CNV identification as a priority within the diagnostic approach, especially in BMD. Finally, the “small-in-frame” trend does not widely occur for small mutations in BMD, apart from, although partially, nonsense changes, and therefore understanding why detrimental small mutations do cause DMD or BMD phenotype needs further investigation.

We believe that reporting data on such a large patient cohort might be extremely valuable for many aspects, from etiopathogenesis to therapy, as a large number of patients can allow for a better understanding of disease mechanisms and causative events.

## Data Availability Statement

The datasets generated for this study can be found in the LOVD1401 database, www.lovd.nl doi: 10.3389/fgene.2020.00131.

## Ethics Statement

This study has been carried out based on the RARER (Emilia Romagna Region Grant, Area 1A) project, evaluated and approved by the Local Ethical Committee of the University Hospital of Ferrara, Italy (ethical approval n. 139-2012, date of approval December, 20, 2012). Written informed consent was obtained from patients and parents either for the study participation and for the publication.

## Author Contributions

AF, MN, and EM contributed to conception and design of the study and wrote the manuscript. MN, CT, RR, and AnM organized the database and the mutations classification, tables and figures. RS, MSF, NS, AlM, PR, FF, MFa, and FG performed molecular analysis and clinical evaluation of patients. CF, CB, ATos, GA, LM, MMo, VS, TM, AB, AP, RL, MFi, SM, GiuV, ATe, GiaV, MP, SS, EP, LB, EBe, ADA, and LP clinically evaluated the patients. GC, ST, MS, MT, EG, MRP, GM, MC, MMa, CM, EBo, PB, SG, LT, ME, ATor, and VN performed the molecular analysis and the mutation interpretation. GC, CB, MM, TM, MF, ATos, EP, AB, LP, EM, and AF are Health Care Providers of the EURO-NMD reference network. All authors contributed to manuscript revision as well as read and approved the submitted version.

## Conflict of Interest

AF is a recipient of the Sarepta Therapeutics Grant “High throughput genetic diagnosis of muscular dystrophies” and of the PTC Therapeutics Grant “International DMD.” AF and EM are Principal Investigators of Sarepta Clinical Trials Essence and AVI4658-402.

The remaining authors declare that the research was conducted in the absence of any commercial or financial relationships that could be construed as a potential conflict of interest.

## Supplementary Material

Supplementary Table 1 Synonymous variations identified in the DMD Gene in our cohort of patients.

Supplementary Table 2 List of splicing mutations identified in our cohort of DMD and BMD patients.

Supplementary Table 3 Blast analysis of the introns 50 and 52 revealed an anti-oriented 87% homology in 861 bps.

Supplementary Figure 1 Exonic distribution of deletions and duplications in DMD and BMD patients DMDs have 57% of deletions and 11% of duplications. The most common single exon deletion in DMD is exon 45 while the most common multiple exon deletion is 45-52. Deletions were non-randomly distributed, occurring in the two known hot spots at the 5’ and 3’ end of the gene and very heterogeneous.

Supplementary Figure 2 Geographical distribution of nonsense codons in DMD and BMD patients The three codons are almost equally represented in the national mutation spectrum; regional distribution showed that in Northern and Central regions this trend is maintained with TAG slightly more frequent than the others (38% North, 47% Center). In Southern and Sicily the TGA codon is the more frequently occurring nonsense mutation (49%). Sardinian DMD show only TGA and TAA nonsense codons, while in BMD only the TGA codon (100%) was found.

Supplementary Figure 3 Geographical distribution of skippable deletions in DMD patients Northern and Central regions show very similar percentages of skippability, being the exon 53 the more frequently skippable exon, South and Sicily have a different pattern being exon 45 the predominant skippable exon. In Sardinian patients, only exon 44 skipping is applicable in DMD patients.
