# Supplementary material for: The Genetic Landscape of Dystrophin Mutations in Italy: A Nationwide Study
Source: Front Genet. 2020 Mar 3;11:131. doi: 10.3389/fgene.2020.00131 (PMC7063120; doi:10.3389/fgene.2020.00131)
Supplement: Supplementary Table 1 — Synonymous variations identified in the DMD Gene in our cohort of patients. [file Table_1.doc]

| **Mutation** | **Phenotype, age** | **Effect** | **Annotation** | **Exon** | **in-frame/out-of-frame** |
| --- | --- | --- | --- | --- | --- |
| **c.7341A>G**  **p.Gln2447Gln** | DMD, 8 yrs | creates a cryptic acceptor splicing site leading to a flanking intron 51 region incorporation | Not reported on lovd.dmd | 51 | out-of-frame |
| **c.4299G>T**  **p.Gly1433Gly** | BMD, 41 yrs | Located in an exonic splicing enhancer (ESE) leading to a reduced incorporation of exon 31 into the transcript | Not reported on lovd.dmd | 31 | in-frame |
| **c.4299G>T**  **p. Gly1433Gly** | BMD, 53 yrs | Located in an exonic splicing enhancer (ESE) leading to a reduced incorporation of exon 31 into the transcript | Not reported on lovd.dmd | 31 | in-frame |
| **c.3768G>T**  **p.Gly1256Gly** | BMD, 35 yrs | creates a cryptic splicing site within exon 27 leading to exon 27 reduced incorporation in the transcript | Not reported on lovd.dmd | 27 | in-frame |

Supplementary Table 1. Synonymous variations identified in the DMD Gene (Ref seq NM_004006.2)
