# Supplementary material for: The Genetic Landscape of Dystrophin Mutations in Italy: A Nationwide Study
Source: Front Genet. 2020 Mar 3;11:131. doi: 10.3389/fgene.2020.00131 (PMC7063120; doi:10.3389/fgene.2020.00131)
Supplement: Supplementary Table 2 — List of splicing mutations identified in our cohort of DMD and BMD patients. [file Table_2.doc]

| MUTATION | CANONICAL SITE INVOLVED | UPSTREAM EXON | DOWNSTREAM EXON | CLINICAL PHENOTYPE |
| --- | --- | --- | --- | --- |
| c.530+1 G>A | donor | ex5 | ex6 | DMD |
| c.358-2 A>T | acceptor | ex6 | ex7 | DMD |
| c.1331+2 T>C | donor | ex10 | ex11 | DMD |
| c.3433-1 G>A | acceptor | ex25 | ex26 | DMD |
| c.4845+2 T>A | donor | ex33 | ex34 | DMD |
| c.8668+1 G>A | donor | ex57 | ex58 | DMD |
| c. 7098+1 G>A | donor | ex47 | ex48 | DMD |
| c.9287-2 A>G | acceptor | ex63 | ex64 | DMD |
| c.10223+1 G>A | donor | ex69 | ex70 | DMD |
| c.10223 +1G>A | donor | ex69 | ex70 | DMD |
| c.2949+1G>T | donor | ex21 | ex22 | DMD |
| c.6118-1G>A | acceptor | ex42 | ex43 | DMD |
| c.8390+1G>A | donor | ex56 | ex57 | DMD |
| c.1603-2A>C | acceptor | ex13 | ex14 | DMD |
| c.9287-2 A>G | acceptor | ex63 | ex64 | DMD |
| c.9287-2 A>G | acceptor | ex63 | ex64 | DMD |
| c.94-1G>A | acceptor | ex2 | ex3 | DMD |
| c.94-2A>G | acceptor | ex2 | ex3 | DMD |
| c.649+1 G>A | donor | ex6 | ex7 | DMD |
| c.831+2T>A | donor | ex7 | ex8 | DMD |
| c.1150-1G>T | acceptor | ex10 | ex11 | DMD |
| c.1331+1 G>T | donor | ex10 | ex11 | DMD |
| c.1482+1 delG | donor | ex11 | ex12 | DMD |
| c.1812+1G>A | donor | ex14 | ex15 | DMD |
| c.1992+1G>C | donor | ex15 | ex16 | DMD |
| c.2168+1G>A | donor | ex16 | ex17 | DMD |
| c.2168+2T>G | donor | ex16 | ex17 | DMD |
| c.2169-1_2169delinsAC | acceptor | ex17 | ex18 | DMD |
| c.2949+1 G > A | donor | ex21 | ex22 | DMD |
| c.2950-2 A>G | acceptor | ex22 | ex23 | DMD |
| c.2950-2A>C | acceptor | ex22 | ex23 | DMD |
| c.3162+1G>A | donor | ex22 | ex23 | DMD |
| c.3277-1G>T | acceptor | ex24 | ex25 | DMD |
| c.3433-1 G > A | acceptor | ex25 | ex26 | DMD |
| c.3604-1G>C | acceptor | ex26 | ex27 | DMD |
| c.6614+1G>A | donor | ex44 | ex45 | DMD |
| c.6913-1G>A | acceptor | ex47 | ex48 | DMD |
| c.7543-2indsdupAGGCAAC | acceptor | ex51 | ex52 | DMD |
| c.8027+2 T > A | donor | ex53 | ex54 | DMD |
| c.8218-2A>G | acceptor | ex55 | ex56 | DMD |
| c.9085-3_9085-2delinsT | acceptor | ex60 | ex61 | DMD |
| c.9287-2A>G | acceptor | ex63 | ex64 | DMD |
| c.9563+1G>A | donor | ex64 | ex65 | DMD |
| c.9563+1G>A | donor | ex64 | ex65 | DMD |
| c.9563+1G>A | donor | ex64 | ex65 | DMD |
| c.9808-1 G > A | acceptor | ex67 | ex68 | DMD |
| c.10086+2T>C | donor | ex68 | ex69 | DMD |
| c.10087-1G>C | acceptor | ex69 | ex70 | DMD |
| c.10223+1 G>T | donor | ex69 | ex70 | DMD |
| c.10223+1G>A | donor | ex69 | ex70 | DMD |
| c.10223 + 2 T > C | donor | ex69 | ex70 | DMD |
| c.10223+2 T>G | donor | ex69 | ex70 | DMD |
| c.10223+2 T>G | donor | ex69 | ex70 | DMD |
| c.94-1 G>T | acceptor | ex2 | ex3 | BMD |
| c. 3162+1 G>A | donor | ex22 | ex23 | BMD |
| c. 3162+1 G>A | donor | ex22 | ex23 | BMD |
| c.358+1G>T | donor | ex4 | ex5 | BMD |
| c.358-2A>T | acceptor | ex5 | ex6 | BMD |
| c.650-1G>A | acceptor | ex7 | ex8 | BMD |
| c.1812+1G>A | donor | ex14 | ex15 | BMD |
| c.1603-2A>C | acceptor | ex13 | ex14 | BMD |
| c.186+2T>C | donor | ex2 | ex3 | BMD |
| c.1602_1602+1delinsAT | donor | ex12 | ex13 | BMD |
| c.1602+1G>T | donor | ex12 | ex13 | BMD |
| c.1603-2A>C | acceptor | ex13 | ex14 | BMD |
| c.1704+1G>A | donor | ex13 | ex14 | BMD |
| c.6912+1G>T | donor | ex46 | ex47 | BMD |

Supplementary table 2 DMD Gene, Ref seq NM_004006.2 Canonical splice site mutations identified in our cohort of patients, flanking exons and associated clinical phenotype
