# Supplementary material for: The Genetic Landscape of Dystrophin Mutations in Italy: A Nationwide Study
Source: Front Genet. 2020 Mar 3;11:131. doi: 10.3389/fgene.2020.00131 (PMC7063120; doi:10.3389/fgene.2020.00131)
Supplement: Supplementary Table 3 — Blast analysis of the introns 50 and 52 revealed an anti-oriented 87% homology in 861 bps. [file Table_3.doc]

Supplementary Table 3

BLSTN analysis of intron 50 and intron 52 of the DMD gene

INTRON 50 full sequence

GTAAGTATACTGGATCCC

ATTCTCTTTGGCTCTAGCTATTTGTTCAAAAGTGCAACTATGAAGTGATG

ACTGGGTGAGAGAGAAAATTTGTTTCAATTCTAAAGATAGAGATAAACCT

TTGTGTTATTGACTGTGCAAAAAGTCTTAGAGTACATTCCTTGGAAATTG

ACTCTGATTCAAAGTGTTGCATGACAACGGGATATGGGGAGTGTTCTCTG

GAGATACACCCACAAGGAAGAGAAGAGCACAAGGGAGATTGTGGGAGAGT

CTGAAATGTGATTTGTCTGCAGCAGAGGCCTAAGCCAGTCTCGCAGGAGC

CCTACATCTGGGCTGGCTGTGCAGAGCTGTCCTGAATTGCAGGCAGTGGG

CCTGGCCCTTGTATTCCTGATCCAGCCAGCCATTGGCCAGGGGCTGGCTG

CTGCCTGAGAGTGGAAGGACAACTTGGACAAGTTTTCTGAGGCCGAAGGC

AATTCTTAGTAAGGAACACCATTAACAACCAATATTCCTAGCATCCAGGG

ATGTGTGCATTGTTCCTGAAGAGGGACAAGTATGTCTACAAAAATCACAG

AAACCACAGAAACACACACAGTCCTACTAGCACCTCTCCCTGTCCCATTT

GCAAACAATTTAAGAGCTCTCCCATTTTTAGTTCAAGAAAAAGAAAAATG

GATTGGGAGGACCACAAGCTGACTTGGGGGAGGAATATTTCCTCATTTAG

CTGTAGTTTTAACTTTTGTTTTCACTGCATATTTTCAGTCTATTTTATTT

TCTTTCCTCTTCAGTTGTTGATAGAAGGTATTCATAAATTCTCATGGCAA

TGTTAATGCTGGCTTTGACTCTCAGGGGAAAGAGGCCAGAAAACTTCTTT

GCTGTACCATTCCATAATTAGGCAGAACTAAAAACATCTTTGGGTGTTGT

TTTTTGTTTTTGTTTTTTTTTTTGCCTTGTCTGCTTTTCAAAGATCAAAT

GATTGAAGCATTAAAGCATGGTGACTGGTTCTTCAGGTAAAGTTGATTTT

TATTTTATGTCAAGTAGAAAAATACTGAACTGGAAGAATCACAGCTGGGG

TAGCACAATCATAATTCATTAGAAGGCATAAATAGTGCTTGGATTAAAAG

AAGCCCTACAATCTGGGGACAGTGCATCTCATGTGCCCTCTGGGATTACT

CGGCAGTCATCAGAGTTAGATTTAACGACTTTGGAGACTTAAGCATTATG

GTTTTTTTTTTTTGTCAATCTGGGACACTGAAATTGCTGTATCAGGGTTA

TACTCAACTGTGTCAGGTTTATTTGTTTTTATGAGCTGTAATTTTTGGTT

CCCTCAGCGCATATGCATAGTTTGTTCCTATGTTATCATTTATTGGTGTC

TGTTTTCTGGCTGTCTCTGGTAGGTTCAGCCTCAGACTCTGTAACTCCAT

GAAGAGATTATGTTCCAATGATGTTTTATAAGTTTGTTAAACTCTGAACT

CATGAGTTTATGTCCCATATAAGCCACGTTACACATGGTAGGAAGGCTCC

AAAACCAGGGCGCCGAAATCCATTTAACGTGTAACTTACCTAAATGTAAC

AATGTTTATAAGAAAAATACATTGGAAGTTCCAGTTTTGACTTCCAGCAA

CATATATAATTCATCCACTTTATTTATTAACTTCCATGTGTTGAGCATCA

TACTGGTGCTGCGAGTACAGCATAGAATAAAAGTCTCTCCTTTCATAAAA

CATATATTGTAATTGAAAGAGAAAGACAATAAACTAATGAAGAAAATATA

TACTGTCTCAATAATTATAAGTGCTGTAGAGTGTAGTCTACAGATTGATG

TCAATGGGTATTTGTTAGACACACAGAATCTGAGGCCCCTATCCTAGACC

CACTGAATCACAATCTGCATTTTAATAGAATCCCCAGGTGAATCCTGTGC

ACACTGACATTTGAGAAACACCATTACAGAGAACAACTAAGCAGGGAGAT

GGGATGAGGGTATTATTGTCTATAGTGTGGTCAGGGAAGCTTGTCTGTTA

AGAGAACATCAGAAAACTGATGTAAGTGAGGAAGTGAGCCTGGTGTATTT

CTGGGAAAATTATTCCAGGCAGGGAGAGAAAAGACTGAGCAACGATACTG

AAGTAGGAACAAGATGACGGAATATTAAGGAGATCAGTGAGACTAGAGGA

GTGGGTCAGGGGAAGTGTGATGGAAGCCATGAGAGATACTCATCTTTCAT

AGCACTGCCCTACTTCCTTCTCCCCAACATGAGGGTCTCATCACCCCCCA

CCACTCTTGTCTTCTCCTATGTCCTCCACATTGCTGCCAGTATGGAGAGT

CTGGGAATGCCCTCAGCTCAAAGCTGTTTGGTGATAGCTGGCAGAGTTGT

GGTAGTAGCTAAAAAAGAATTAAGGGAAAGAGGAATTTTCTCAAAAGCAG

GTGCTTTTCATCCTCTTTAGCAAACCGAAACAGATCTGAGCATTAAGTCA

AGATGTTAAATACACAAATGTTGAATGAAAAAAAAAACAAAAGGTAGTCA

TTTAAATTCAGAGCTGCTTTTATTAAAATAAGATTTTCTTTTTTCTTTAC

TGTGGTAGTTCAAATATCAGAATAAAGAATTGTTTCTATTCCCGACTTCC

TGACTTGCAGGAAGTTAATCAGAAATAAATGCAATATAAAAAAAGAAAAT

CTAATTTGTATTATGCTTCTTGTATATGTTTATTATTTCATGTACTGTAT

TACAATGTAATAGAATTTATAATTCATTATAGCAGATTGTTTCCATTGCA

TTCCTACTATTAAATATGTAGAAGCTACACATATACTTGTAGCTTTAACA

TATATGTCTTTATCCTCAAAATAACTGCAAAGAACATATAGATAATTTTT

AAAGATTAAGGAGCCTGAGGTTTAGAGGGGAGATAGCTAGATTAAGGCCA

CACAGCTAGAAAGCAAGCAAGCAAGGGTTTCAGTCCACATGTCAAGCTCC

ACAGCCTGTGTTTTGTTTTGTGGCTGTGCTTTACACTATCTCTCTGTCCA

AGAACCTAATGGAAAATTACAGATACAGATGCAGCTGGCCAGCAGTTAAT

ATAATTTAACTCAATCTTAAATTTATCTGGAGTAAAAGTGATACAAGTTT

CCGTGTTTTTTCTTTTCTTTCTTTCTTTTTTCTTTGTGTGTGTGTGTGTG

TGTGTGTGTGTGACAGAATCTTGCTCTGTTGCCCAGGCTGGAGTGCAGTG

GTGCGATCTCGGTTTACTACAACCTCTGCCTTTCAGGTTCAAGCGATTCT

CCTGCCTCAGCCTCCCAAGTAGCTGGGATACAGGTGCGCACCACCATGCC

CAGCTAATTTTTGTAGTTTTAGTAAAGACGGGGTTGCTCCATGTTGATCA

GGCTGGTCTTAAACTCCTGACCTCAAGTGATCCGCCTGCCTCAGCCTCTC

AAAGTGCTGGGATTACAGGCATGAGCCACCTTGACTGGCCTGTTTCTGTG

TTTTTTCTACTTAAGAAGTAGAAAAAATTGGTTCACTCTATTTGAATTTC

TTAGAACCATAGAAATCCAAACTTGGAATAATTATTGCAATTATTATCTA

GTTGAGTATTCTTCTTTTATAGATGTAGAAGCTGAGGCCTAAAGTTGCTT

GTTTTGTTTTATGAACACTAAACCAAACTACCTTGTGGCTGCTTACTTAA

ATTATAAATTATAATGGGGTGGCCTTGCCTGAGCTGTATAAATTGTTTTA

ATTATCAGGACAAATCAACATACTGGAAAAAAAAAGCAAAACTTGCAATT

GTTGTTGCTTAGACACCTGTCCATATCAGTTTCTATTGTTGCATAGAAGC

ACCCCAAAACTTAACAGTTCAAAACGAGTGATTTATTGCTCATAATTCTG

TGTGTCTGAAGTTTGCTCTGTGATTAGCTGGATAGTTATTCTGATGGTCT

TGCTTGGTGTTACTTATGTGGATGCAGTTATCTAGCAAGTGAACAGGAGC

TAGATGGTCTAAAATGCACTCTCTCAGATATCTGACAGCTGGCTGTTGGT

TGCAGAACCTTGATTCTTCATAAGGCCACTCATCATCCTGTAGGCTAGAT

TAGGCTTCATTACATGGTATTCTCAGGGCAGTTTTCCAAGAGAGGGCGGG

TGGAAGCTACAAGACCTTCTGATGCCTAGGCTTTGAAACGTGTAGGTTAC

TTCTGCTAAGTTATATTGGTCAAAGCACCTCAAAAGATCAGCCCAGATTC

AAGAGATGAGGAAATTACTTCATCTCGTGAAAGGAGGAGATGCCACATCG

CATATCAAAGGGGTATGCATATTGGGGATAGAAGGTTTTATTGTCACCGT

ATTTATACACAAATCACTACATTGGGGAAAAGGAGGGAAACTGGAGATCA

AAAGTGTTGGTCTTAATCCAACTTAATTCTCAAAAATTACCATGCGTTAG

AACCACCCAGTTCTTCGCAAAGTATAGATTACTGGGCTTCAACCCCAGAG

TTTCTGATTTACTAGCTCTGGGGTGAGACCTGCATCTCTCTCTCTCTCTT

TTTTTTTGAGACTGAGTCTTGCTCTGTCACCCAGGCTGGAGTGCAGTGGT

GCAATCTCGGCTCACAGCAACCTCTGCCTCCTGGGTTGAAGCGGTTCTCC

TGCCTCAGCCTCCTGAGTAGCTAGGATTATAGGCACCCGCCACCACGCCT

GGCTAATTTTTGTATTTTTAGTAGAGACAGGGTTTCACCATGTTGGTCAG

GCTGGTCTCGAACTCCTGACCTCAGGTGATCCACCTGCCTCAGCCTCCCA

TAGTGCTGGGATTACAGGCGTGAGCCACTGTGCCCAGCCAAGACTTGCAT

CTCTGAAAAGTTCCTAGGTTATGCTGATGCTGGCCTATGCTTTGAGAACT

ACTACCACAGACATACAGTGAGTGGGGAAGAATAAATTCATCCCTTCTGC

TGTGTGCAGCAAGGAGTGGGATTCCAATGAGATCCAGTGCTGTGAATGCT

AAAGGGAAATCCATCTTATTTTAGCACCTCTACTCCCCATCTCCCCACCC

CGAGGATGTTATAGCTTAGAAGTTCAAGGAGATGGACAACACACTAAACC

AGGCAGTATTTGCCCTGCAGAGCTGTTCAGTGTTCCTGGATGAGACCTCT

GAGAAGAAAAGCCATAAGTTCCTCTAGAGACTTTCACAATCATTTAGGTA

GACAGGACTTTGCATGGGTCTGAAGGCTTGCATGGCAGATGGAGGCAAAG

AGCCAGCAAATCTGGTTGTAAATGTCAATGTGAATCCTTTCTTATCCACA

AGCTGCTGGGCCTGAGAACATTAATGTTCTACAATACCCGATTTAGCATT

TTTGAAAGAAATTGCATATAGACATGCTTAATGTGAAGACTCCAAATCAG

GATATTTGATTCAAATGTCTCTTGGTAATAACTATGGAATGAATAACCCA

TTGTATATGGACATATAGAAGAGCCAGTTAACAGAGTTTTCTTTTTTTTT

TTTTTTTTTTTTTTTTTTTTTTTTGAGACGGAGTCTCACTCTGTCGCCCA

GGCTGGAGTGCAGTGGCGCGATCTCGGCTCACTGCAAGCTCCGCCTCCCG

GGTTCACGCCATTCTCCTGCCTCAGCCTCCCCCGTAGCTGGGACTACAGG

CGCCCGCCACCACGCCCGGCTAATTTTTTTGTGTTTTTTAGTAGAGACGG

GGTTTCACTGTGTTAGCCAGGATGGTCTCGATCTCCTGACCTCGTGATCC

GCCCGCCTCGGCCTTCCAAAGTGCTGGGATTACAGGCGTGAGCCACCGCG

CCCGGCCCAGAGTTTTCTAGTTGATTAAACACTGGTAAAATCATCTCTTC

CTGTAATTAAGTTTGGAGAGAGCAAGTCTCAAGTGTAATTAGGAAAGCAC

AGTATTGGAGGTCAAGAAGCCTAAATTCTGATATCTCAGTTGTGCTACCA

ACTATATAAGGGATCAACAGACTGTTTCTGCAAAGGACCGATACTAGATA

TTTTCTGCTTTATGGGTCATGTGGTCAACTCTGTCATTGTATACTAAAAG

CCACAGACAATACCTAAATAAAGGGACATGGCTGTGCTCCAGTGACACTT

TATTTACAGAAACGAGGCAGGTAAGATAGATTTGACCCTTAGTGGGCTAC

CCATTTCCCCTTAGCACTCAGCCTTCCTATAAACCATGAAGCGTCTAACT

ACAAGTACCTGTGAGTCTTCCTGAGTCCTTTCTCTGCCTGTAGGAGTAGC

TCTCCCACTTGCAGAGCAGGCTGGAAGCTGGGGAGGAGATTATCTCTGGA

ATAGCACTTAACCAATGGACAAAAGCTGGGGGATAAAGGTGTTGGTCTTC

ATAGTTTTCATACTGCTATGAAGAAATACCCGAGACTGGATAATTTATAC

AGGAAAAAGAAGTTTAATGGACTCACAGTTCCACATGGCTGGAGAGGCCT

CACAATCATGGCAGAAGGCGAAGGAAGAGCAGAGGCACTTCTTACATGAC

AGCAGGCAAGAGAGCATGTGCAGGGAAACTGCTCTTTATAAAACCATCAG

ATCTCATGAGACTTATTCACTATCAGGAGAACAGCACAGGAAAACCCCAC

CCCCATGATTCAGTTACCTCCCACATGGTCCCTCCCACAACACGTGGGGA

TTATTGGAGTTACAATTCAAGATGAGATTTGGGTGAGAACACAGCCAAAC

CATATCAATAAATATCCCACCTTCTTCTCCCCTTGGGTAGGACACCTCCA

ATGCATGTTCCACACTAAAAATCTCCAGTGAATTGGGCAGTTGACCACAA

TGATAAGCATACTTATTAGCATGCCTCGTATAGGCTTCCTTCCGATGTGT

AGGCTTTCTTCCCATTTTGGCAGAATCTGGACCCAACCCCACATCTCCTG

AGTTTGATTTCAGTCCTCTTCAGTATTCTCATATATCTCAACTTTGTTCC

CTGCTGAGATACAACTGACAGATATATATGTATCTTCAGCTCTGCAGCTC

TATCTTCTCTTCTACCATGGCTTTCTGGGATCAGCTCCCAATTGAATTAT

TTGTATTTAAATCCTTGTCTCAGGGTCTACTTCTGAGCAAACCTAAATTA

ATACAAGTCTTAGAATTAGTGCCACAGTTATACCTTTTGAGTAAGTTATG

TTGCAATTCCCATTTTATAGATGAGATAACCAAGGGTCAGAAAGGTTAAA

TAATTTGTCCAAGGTTACAGAGCTAGTCAGTGACAGAGTGGGTATTCAAA

AATAAGTCTCTATGATTCCAGAGCTTATGCTATTAATCAGTGCACCATGT

ATCAAGCAGTGATTTAGTTATCTTCATTTGAATTTTTTGAAGTCTCTATT

TAAATTGAATATCAAATCCTCACATATAAGGTTATTTATACCTTTTTATT

TGTTTTATTTATTTATTTATTTTTGCTTAACTTTTTTTTATTATACTTTA

AGTTTTAGGGTACATGTGCACAATGTGCAGGTCTGTTACATATATATACA

TGTGCCATGTTGGTGTGCTGCACCCATTAACTCGTCATTGAACATTAGGT

ATATCTCCTAATGCTATCCCTCCCCCCTCCCCCCACCCCACAGCAGGCCC

CAGTGTGTGATGTTCCCCTTCCTGTGACCATGTGTTCTCATTGTTCAATT

CCCACCTGTGAGTGAGAATATGCGGTGTTTGGTTTTTTGTCCTTGCGATA

GTTTGCTGAGAATGATGGTTTACAGCTTCATCCATATCCCTACAAAGGAC

ATGAACTCATCATTTTTTATGGCTGCATAGTATTCCATGGTGATTTATAC

ATTTTAATTTGAACTTCACTCACCTCTACAAAGTTAAATAAAGCATTCCA

TTTCCATTTCAATAATACTTTAAATATCTAGGCAGTTAAAGCATGAGGTT

ACTTGGCACTTAAATGTACTCTTGTCATACTACTTTTGTTGGCTTAAATT

GCAAAAAAAAAAAATGGTTAATTTATTGTGACAATACCCTACATTTATCT

GCAGTGATCATTTTTTTTGTGAAAATGGCCTTCGTTTATCTGCAGCAAAG

GAAAAAGAGGATGGCAATTAGTTCTTGCATTCTTATTCCTCTCTTGGGTC

CTGATCCTTCTCATTAATAGAAACATGGCAGGGGAGGGGTATATAACCCA

CACCCTTTCCTGTTGTGGTTATGTTTCCACTGTTGATTCTGCTTCAGGTG

AACCTTTAGGATTAGGCAAATAAATTTCCGTGAGGCCAAATCTTTTTCTT

CCTCATTAACAGATGATTTCTCTGCTAAAAACACTTACGACATGGCTATA

CTATTGCCGGTTTTATAGTTACAGGCTCTAAACCTTGAAAACTTCCTCAA

AGTCTAATACGTCAGGAGCAAGCTTTTGTACAAAAAATGTGAAGACCCTT

AATCAGTTCCAATAACAAAATAAATCCATTTTAAACCCTATCCCAAGATA

CTGCAAGGCCTTGGAGCAGCTGGAGAGACTCCTTAACTCTTGACATTAAT

TAATTAATTTAAAAATTCATATTTGTATGTATCAGTGAGGTAAGAGTGCT

TGAAATATAATGAGATGTGTCACACTGTAGAAAGGGGAGTGACAACAGAA

AGCCCTGGCTGGTGAGGCCCCAGCACTTCTCACACTCATCAGAAGGAAGT

CTTTCCATGAAGGCAGTAGGGGGTCCCTGGTCCCAGGCCAGGTCCTTCAA

CAATGCTCACTGGTTAACAGGAAAGGCACTACAGTGGCATCATTGTTAAC

ATCCAAGACAGTGATAGAATGTGAAACTTCTACTGTTTAGTATTTAGTAT

TCAGCATTTAGATGTTAATTATCCATTTTGTGATGAAGTTCCCTTTCTTC

TCCCTCTCTTAACCTTTTGGTAGTTTTATTGCATGGTTACCATTTCCAGT

TAGGGTTGTGCTTTGGGGTCTGAACTGATGAAGGAAGAGAAACTCTAGTT

CATCATTTCTAGGAAAAAGAGAAGGCTAACATCAATTCTGATGATTAGAG

ATTTTTTGATTACCTATGTCCTGCATTTTAACAGAAATAAAATGTTAATT

ACAGTAAACTTACTTTAACCTTCTTATGCTATTTAACACTCTTCAAGAAA

GGACTTTTGCTTGAAATCACTACAGAGTATACCATAATCTTGACTGTCAT

ACTCTGACCAAGAAACCAGAACTATTTAGGCATATTTATGAGAGTAAGTA

CCCTACCGCTCAAAATGGAAGGCTTCAACATGTATTCCTAATATTTCCTA

AAACTTCACTGAATAGTTTTAAAATTGAATATAACTTTACCTTCAGAGAG

AAACAAGATTTCGAAAGGAAATTGTAGCAGTTTTGTACTTCAATTGTCGA

TTTTAAGATTGTGGTCTGAAAAGTATTTGAACAGTCATTCTTTCTTTTCC

ACTATCTCAGGAAAGGCTCTCATTCTATTAGAAGCAATCTTAGAAGCGTA

ACTGCCAACTCTCTTCTTAAAAAGTGAAGAGCAGATGGAGTTCTACCAAT

CTGCTATACTTGACAGTTTTTCAACTACCTAACCTAAGTTCCAGTTACTG

TCTAAATTATTTTTATTCGAATAGGAAAAATACATTTATGCTTACTGTAC

ATTTCGACAGTGTTCATTCTTTGAGGTCTGTTATTTAATCTCCTTCCCAC

TTTTCTTTTCTATCTTAAATCAGATGAGTCCCCATGGTCTGGCAACAAAA

CAGCTGTTTCTCAAATTTTTAACGTCTCTGTGGCCCTCCTCCTACCCTTC

CAAGTGTTTTCCATACTATTTTTCTGTTGTGGGCTTCAGAATTGGATGCT

GTTCCATAATTACTGGTCTTCCCATGACTCTGTCACATCATAAAGTCTAT

ATATACCCTTTTCCTACGTTTACATGCCAAATACCAGGTGTGTCTAGCTT

TAATTCAATGACTAGCATATGGTAGGTATTGCACTAATATTTTTTGGTCA

AGTGAACATATATTTTACAGATAGATCAAATACATCAACCTTCCCATGTA

ATAATAATAATACCAAGAAGATTAAATGATCGCGTGCTTGACCAGCTGTT

TAGTGGCAGAATCTGGACCCAACCCCACATCTCCTGAGTTTAATTTCAGT

GCTCTTCAGCATTCTTATATATCTCAACTTTGTTCCCTGCTGAGACACAG

CTGACAGATATATATATATATCTTCACAATCTCAGTAGTAAGCCCTATTT

TTTTAAGATTACAGTGCTGATCAAAGAGGGATATTCTATGGCATTGATGC

AAATCTTCCAGGCCAGATATAACCCCTATCTCCTTAAACCCTCATTCTTG

ACTGTGTCTTGAAATCAAATTTGAGAACCTCTGCTTTACATGCTACCTTC

CTCTTAAAATTCATAAGGTTTATTCCTTCTCTCATCTTCAGTATTTTTAT

GAAAATATGTTTTACTCTTATTTCCAAAGTGCTTGCCAGCCCATGCTGAG

TTCATTAGACAAACAATCTAGGTATCCTAATATTTAGGTAAAATTGCTAG

CAGCACTAACTATACCAGTACCCACTTAAATTGCAATATAAACCTACAAT

AGGAAAAAAAAAGTCAAAATTATACTACTTTCAATTCCTACTTCTGGAAT

AATTATCACACCTTCAAAAAAACTCATAATTGTTCTCCAATATTAAAAAC

CAGGAACTAAATTACATCACTATATATATATAGCTATATAGACATATAAC

TGTATATAGTTTATATATATATTACATGTCACTATATATGGTTATATATA

CAGATAGCTATATCTCTATATAGTTTTTATATAGGAAACTATATATATAA

ACTCATATATATTTATATATAAACTCATATATATATAAACTCATATATAT

TTATATATAAACTCATATATATTTATATATAAACTCATATATATATAAGA

GATTTTATATGTATATATGAGAGACAATATTGAAATAAAACAAACTAGCC

AATCTCCAATGTCCCTTCATTTTCCCAGGACTCTTCTTTATTCTCAAAGA

AATGTATGAATACACAATATAAATAAATGTTCTATAATGTTCATTATGGA

TTATAATAGTTTTCTTGTCATACTGAAGTGAATGGGAATTGTTTCTTCGA

TTATTAGCATACTTTTCATAACTATGTGATTTGGTGATAGGCACTTTTGC

TTACTAAGTTCAGCATGATCAAAACAGAACTCCTATTATTTTTATTGTTG

ATTCTTATTTCTTTAAGGTAAAATATGCCCTTTTCCTTTGAACCTGCACA

CTGTTTAACAAATAAGCACATAACTCAGGATTGAATTGTACACTTCGATT

TGAGCTTTTTTTTCAAGGTCAGGACCCAGTTTCACAAGAAGTTTTATTTT

TTCCAATACAACTGACATCCACTCCCACCAGCTGAAAGACAAGAAAAACT

TGTCTAATAAAGCTTTCAGATTCAATTTGCTGCCTGCATACAGCTTGAGG

AATCTCTGGAGGTCACTCACAGCATGTGTTGCAACCCCAACAGGGAGAAG

TAATGAAAAGATTCTAGTTAAAAAGCTGACACTGCCCCTTCCAACCTCTT

TGAATGTGAATATAATAAGCCAGTTTACAGACGCAAATCTCTATGATTCT

GGGGATTTCCATCTTGATCTCTGACTCCAAGGAACATTTGAATGCATGGA

TTTGTATCCATTATCTGGGTGAATAAATGCTTCATATTGAAAAAAGGGGT

GCTTTAACAACATAAGTCTGATGTAAATCAGGCAAAACAACATTGTCACT

TCATGTTTAACTCTCCTGGAGGGTCTCTAAGGTCTCACAGTTTGGTTCTA

TTCCAGTAATATATAGGCCTATCATAGCCATTTTCAAAAATAATACCTGC

TTTCATTTCGATTATTCCCCCTAGCTTTTGCATTGACCCGAACATACCTA

ATATTTATCTTAGGGCTAACACGCATTAATGCCTTGCTCTGTACCAGGCA

TTTTGCCAAGTATTCTTTGTGCATTTTTCTGTTTAATCTTACAGCAGCCT

TATGAAATAGGTACTACATTATTATTATTTTTCACCATGAGAGGAAATGA

AAGCCTAGAGAGAATGGTTATCCAAAAACACCCAGCTACTAAGTGGGCAC

AGCATGGCCTTGAACCTGAGTCTTTATAAAGTTCATGCCTGTCTTTTACC

TTTATGTTAAACATACTGAATCTTGGTCATGCAGTCTATGAATGAAGACT

CCATATACTCTAGGACCAATTCTACCATATTGTGCATGCTTTTGTACATA

TTTTCCAAATGAAATTAAACAACAATACTCTCTCTTCCCCTTTCTTTTCC

GTTTTGCATGACATCTAAATCTTTTATTAAATCTCCGTGGGTGGAATTGG

GCCTTAAGTAGTAGTACCTTTGAAGCTTAATACTATAACCTCAGAGTTAC

GGAAGTGGTTTCAATATGAAGAAATATATACGTTCTTTTCTTTTCTTTTC

TTTTCTTTTTTTGGCTCCTCTAGAATAGAAGGCAATCAGGAGAGAAAAAG

ACATTAAGATGATAGGCTTGATTCTCCCACAGTGTTACTACTTAGCTTTA

TTTATTCTCCCACATCTGTAACTGTCAATTCAAGGTCAAAAAGCCAGTAC

AGCAGCAGAATTACAAAAAGTAGATCTGGAAAATCTATAGGGTACCATAG

TCCAGCACCCTGCTGCAAGTCAAAATCAATTAAATAAATATGTTTAAACA

GCACTTCATTTAATCTTAAGGGCACCACGACTCCCTTGGAAAATTCTTTT

TTGTGTTCAGACCTGATTCTCTGAAAGTATTTTCTAGTGTTTTCTTTGTT

CGGTGGCTGTGGTGAATAAGTGGACCCAATTGCCTACAGCATGAAATCGT

AGAAATGAATATGGGCTGGAACTTCAATCAATCACACAAACCAGAAACAT

AGAGTTCATCACCTCTTCTGGAAAGCGTCCCCTTCGGCCGTCAAAAGGGT

AGAGTTTCCTCCAACATATGTTCATACTTCCCTTTGCCTCCCATCTGCAA

TTTCAACCAGTCTGTTGCTCTCGCCCTTTTTAGGCTTCACATACCGGATT

TCTTCTTAGGAGCTCACTTGAAAAAAGGGTTGTATGCTTTAAAAATACTA

AAAGTCACTGGACTAGATGATAATAAAATTTCTGTAAAATAAAAAGGGAG

TTAACAGAGGTGTTACCACTTATCATAATGGATATTGTTGACCTTTCCTC

TAGCCAGTGTCCCACTTAAGCATGCTTCTTGGCTGGAATTTATTTTTCTC

ATCTGGATAAATCTGAAGCATTATAGTAAAACCCAGTGTGAGCAAGGCCA

TGGATGCAAGTGGATTGAGATGAATGAGTGGATAGACCTGCGTGGGCAGA

AATAATGAGGTCAGCAATAAGCCATACCAAGGGATGCACACTCAAGGAAT

AAATTGGAGGTAGCAGAATAACTGAACAAATGATACATTTGATCAGACTG

TCACATGAATATCAACTGAAACAAGTTATGACTGTAGTTAGTGAACCCTA

GAGTGAAGAAAACAAAATATTGAAATCCATCTGATCAAAAAATAATAAAA

TTGTTTACATGTATATATTATCTAAAATTTCCCTTAAGAATGAAGATTAT

AAATTCCCGCAGTTGAGGAAACTGAATAAGAGGAGAATCAGGACAGAATC

TTTTCTCTTCGTATTCCTAGTCCATTTTTCTTCTAACTACAGTAGTGAGA

ACAAGAATTTATTCACCAAATCATAATACATTGCAATTAGGGGATGCCAT

TTGAATCTTGGAAGACACGATTTGAGGCAAATAAAAAATCCATATTTATA

AAGTCAATTATTGGTTTATATAATTTACTGTTTCAGCGGGAGGTATAGGT

TAGTAATGTAAGTCACTTCAAGAAGGTTTTGGTCGAAGTTTTGCCAATTT

TTTCTGTAAAAAGCCAGATAGTAAATATTTTGGGTTTTACAAGCCAGAAG

ATCTTTGTTGAAGTTACTCAACTCTGCTAATGAGGTGCAAAGCAGCCATA

GGCAGTTTGTAAATGCATAAGTGTGGCTGTGTTCCAATAAAAGTGTGTTT

GCAAGAACAGGCAGTTGGTTGGATTTGGCCCACAAGCCGTAGTTCACTGA

CCCCTGTTTTAGATAAATACTAGTCAATAGATTCCTTGTAGCTAATAAAG

GGAATATAGATTGTTTAATGTGTTTCCCTAATCTTCTTAAGGCTTCCTCC

AGGGGAAAAAATATTTCTTATCATCAGCAGTGTTATTAATGCTTACTCAG

AACAAAGAATACCACTTACTTAGCATAGAATGGACATTTATAAAGCCACT

CATGGAAAGAATAAAGAGATGTGAAATACCTGGTACAACTCTTAAGAGTG

TTCCACTATCATTTGAAAAAATGATGAGTAGTAGTTATATAAAAGGACTC

GAGCCTAAAACATTTGTTCTCACTTTGGATAACGTTTTCCTATCTCAGCA

TCAAAAAATACAAAGAAAGGGGATAACTAGGTCTTAGATTTTCTAAATTC

TCATGACTATAAGTCCCATATATTTAGATATTGAAGTACCCTTAAATATA

CCTAAGTAGAAGTAATAATTTCGATATTATGGGAAATCTAATTACTGCTC

ATAGTTCTCGAGATTAAAATGTTATACACCACATCTCAGCTTTCTTTGAA

ACTTGAAATGGCATACTTGTACAAAGGTAGAATGGTCTCCCAAAGCAGCG

TCATAGATTAGCAGAAAAGAGCTATGATAATGCCACTTCAACTGACTATC

ATTTAAATAATGAATTAGTTAGGAAGAGTTAAATTATATAGTTACAGGCA

GCAGGAGATGTCACCATCAGGGGGATAAGAAGCCATTTTCCAGTACACTA

TTTGACTTAACTGGTGCAGTTCCTTCTTTAACTGTAGGTTACTCATTTGT

ATCCTTAATTTATCAATATACTAATCTGACAAATAATTTATTAAGGATTA

CTGTGTGCCCTCCTGTGTGTTAGATTCTAGGGATACAATATGGATCAAGA

TAAGCGTGATTCCTAGACTCCTAGAAAGTAAGAAAACTAAATAACTGTAC

GTGACAAATACTAGGATGTAGAATACTAGGGAAGAATCAGGAAGAATCAC

CTCACCCAGTCTGGAGTATTGCAACAGAGCGATGTCAGAGGAAAAATTCT

GGAAAAAAAAAAAAAAAAGGAAATTTGAAGATGAGACTTGAAGGATAAGT

AGGTGTACCTAGGCAAACAAGTAGGGGAGAGTGTTCCAGGCAAGAGAAAC

AAGTAACAGTTGGAGGCAAAATGGAGCCTAGCACGTTTTAAAAATATCAG

TATGGCTGAAGCACTGAGTTGGGGTTGGGAATGGGTGAGGAGCGATGTGT

CAGGGAGGACATTCCAGATGATGCTGGAAATGCATGCATGTTCGCAAACT

GCATTTTAAGACATGTTAGAGAAATCAAACACATTTTATTTGAAGGGTAA

TTGAAAGCCACTGAAGCATTGTAGAGAGACTAGTGACATGAGCATATTTG

GGTTTTAGAGGGATTACTCTGGCTGAAGAATAGGTAACTGATTGGATAAG

AGCAACTTTGGAGGCTATTCCAGTGGTTCAGGTGATAGATAATAATGACC

TAAACTAAAGAAGTAATAAAGGGAATGGAGACAAAGTAGACAGATTCAAG

TGATACTTGGCACGTGGAAACAACAGGCCCTGGTAACTGATTGGACATAA

AATGGAGAGATACAGAGAACGCGAATAGAACACCCAGGCATTTGGCTTGA

TAAACCGAGTAGACCGTGGTAGAAATTACTAAACTATGGAGTATTTTAGG

GTTCAGGGGAACTGTGAGTTCAATTTTAGACATCTAGTTTTGCAAGGCCA

CTGAGGTAACTGAGCATATGAGTCAGGAGAGAAGTTTAGGCTAAAAATAT

GGATGCCAGAGTTAACCAGCGTTATGATAAAACAAAGCTGAAGTCTCGAG

GGTAAGAACACCTAAACATCTAGGCTGGAGAAGGCAGCAGGTAAAAAGTA

AAAGGAGAGGCCAGGCACGGTGGCTCACGCCTGTAATCCCAACACTTTGG

GGGGCCGAGGTGGGTGGATCACCTGAGGTCGGGAGTTTGAGACCAGCCTG

ACCAACATGGAGAAACCGTGTCTCTACTAAAAATACAAAATCAGCCAGGC

ATGGTGGCGCATGCCTGTAATCCCAGCTACTCGGGAGGCTGAGGCAGGAG

AATCGCTTGAACCCAGGAGGTGGAGGTTGCGGTGAGCCAAGATTGTGCCA

TTGCGCTCCAGCCTGGGCAACAAGAGTGAAACTCCGTCTCAAAAAATAAA

AAGAAAGAGAGAGAGAGAGAGAGAGAGAGAAACAGAGAAAGAGAGAGAGG

AAGACAAAGAAAGGAAGGAAGGGAGGGAGGGAGGGAGGGAGGGAGGGAAA

GAAAAGAAAAGGAGAAAAATTCAGGAGAATGGTTACTTCCAGGGAGATGG

AGGCGATTGTGCTGTGGGGAACACAAGGGTGGGGTCAAGGTATTAGCAGT

ATTCTATTTCTTGATTGGGGTTGTATTTACATAAAGTGTTGCTTTATAAT

TATTCTTCACACTTTATGTGTACGTTCTATGTAATCATCTATAGATAAGA

CAGATTTCACTGTAAAAGAAAATAAAAGCTTCCAAAAGATTATCATCACA

ATTGTAACAGATTCCCCTGGTGCCTGGAGTCACACGCCATTTTCCTGCAC

TGCAGTTGCAGCTGCAGTGGACAGCCCTGTGTGAGTTCAGACTTGCCTTT

AGCTGACAGCATCCCATGTCAAGGGAATGGCTCCCATTTTTCTACTTTCT

ATCTAAGGGACTTCTCTGACATCCCAGGAGCCCACAGATTTTGTGAGCTT

TCTCACCCTTGAAGTTTTAGTGAGTGAGCAACCTTCAACCAATGGAGATG

GGAGCCCATGGATATATTTTTAACCACTATTCCTTCCGGGGGCAAGGGGA

ATTCTCTGTGATTCTCAGGAACATACAAAAGTTCTGTCAAAATACAGTCC

CCATGGTCCATAAGCATTACCTTGATGATAATACATTTGATTGGCATTTC

CTCCCCCTCTGTCTCACTCTTTTGGTTTTTCATTCTTGCTTCCTAGGGAT

CAGCTTCCAAATGAGCTATCTGTACCCAAGTCCTCATCCAGGCCCTGTTT

TCAGGGGACCCAAAGACAACAATAAGAAAAATGGAACTGAAAGAGGAGAA

GACTTTAAGGCATGAAAAAAGTTCCTCTGTATTCCATACTGCATATTTAA

CTGCCTACTCAACAGTTCCACTTAGATGTCTCAAAAATAATCTCATGATC

TTTTACTGCTATGAAATGCATGACCTTCCCCTGATATATTCCTTTCTTCA

GGTTTTGTAGCACCACTTAGCTATCCAGTAACAAAATCTTGGGGGTCATT

CTTAAAACCTTCCCACCTCACCCCTGCTGGACATCCACCACTAAGTTCAG

TTGATTTTTTTGCTCCTAAATATTTCTTGGTTCAGCTTTCATTTTTAGAT

TTATCCTGCATACCCCTGTACAATCTCATCATCTCTTTCCTGGACTGTTA

CAGTAGTCTTATATTAGGATGATCATAGTCTTGATTTGCCTAGAGAAAAC

ATGCTTTATGTCATTGCCTCAGAGTGACTAATAGTCCCACCTTTCACTTG

TAACACTATGCAGGTTATTGGGTTAAATAATACTGTCATCCAAACCTAAT

CTCTCCTCATTCATTTCTTCTCTCCTATGCATCTTAGCTGTGTGAAAATT

CAAAATATAAATTTGATGACAGACAGAACTCTGTTTAAAATGCTCCAGTA

CCTTACCATTTATCTCAAAATAAAAATTTCAAAAAAAGAAATGATATCCC

ACAGGATCTTGTATAGTGTGACCCTTTCCATTTCATCATCCTTATACCAG

ATACTGTGAACTACTTACTCTCTTATCTCCACATTGACCTCCCTCCTAGT

TTTGTTTTGCTTATGAAAGTGCTTATTTTCTGTTTTTTAAAGCCATTGCA

CATATTGGTCTCTCTGATTGAGACACTATCCTTTTGAATTTTTGACCTCA

TACCTACTCACTTTTCAGGTCTCAGCTCAAATGTTATACTCAGGAAAGAC

GCTCCTTACCTCCCAAACTAGGTTAGTGTAAATGGCACTATTTATACTCC

TCTTCCAGAGCACACACCAAATCTTATATTTATTTGTGGGACAATTTGAA

TTCATGTTGTTCCCCCTACTCAATTGTGAGCTTCTTGAGGACATACTCTG

CCTCCTCCTTACCTAGCTTTATTGAAGTATAATTGAGAAATAAAAACTGT

ATGTATTCAAGGTATACAACATGATGATTTTATATGACTATATTGTGAGA

TGATTACCACAATCAAATTAATTAATACATCTAGCACAAGAAATAGTTAC

TATTGTGTGTGTGTTGGGGGGGGGGGATGAGGACACTTAAGATCCAGTCT

TGTAGCAAATTTCAAGTAAACAGTAAAGTATTATTAACTATAGTAACCAT

AATGTACATTAGATCCCCAGACATCTTATAACTGAAAGTTTGTGCCCTTT

GACCAATGATATGGTTTGGCTGTGTCCCCACCCAAATCTCACCTTGAATT

GTAATCCCTGTAATTCCCATGTGTTGTGGGAGGGACCCAGGGGGAAGTAA

TTGAATCATGGGTTTGTTTCCCCCCATGCTGTTGTCGTGATAGTGAATGA

GTTCTCATGAGATCTGATGGTTTTATAAGCATCTGGCATTTCCCTTGCTG

GCACTCATTCTCTCTCCTATCACCCTGTGAAAAGGTGCCTTCCTCCATGA

TTGTAAGTTTCCGGAGGCCTCTGAAGCCATGCGGAACTGTGAGTCTATTA

AACCTCTTTTCTTTATAAATTACCCGGTCTTGGGTATTTCTTCATAGCAG

CATGAGAACGGACTAATACAGTAAATTGGTACTGCAGAGAGTGGGGTATT

GCTGTAAATATACCTGAAAATGTGGAAGTGACTTGGAACTGGGTAAGAGG

CACAGGTTGGAACAGTTTGGAGGGCTAGAAGATGACAGGAAAATGTGGGA

AAATTTGAAACTTCCTAGAGACTTGTTGAATGGTTTTGACCAAAATGCCG

ATGGTGATGTGGACGATGAAGTCCAGGCTGAGGTGGTCTCAGATGGAGAT

TAGGAACTTCTTGGGAACTGGAGCAAAGGACACTGTTGCTAAGCTTTAGC

AAAGAGACTGGCAGCATTTTCCCCTGGCCTAGAGATCTGTGGAAATTTGA

ACTTGAGAGAGATGATCTGAAATTGGAACTTTGTTTTAAAGGGAAGCAGA

GCATCAAAGTTTGGAAAATCTGCAGCCTAACAATGTGATAGAAAAGAAAA

ACCCATTTTCTGAGAAATACAAGCTGGCTGCAGAACTTTGCTTAAGTAAA

GGAGCCAAATGTTAAGCGCCAAGACAATGGGGAAGATGTCTCCAGGGCAT

GTCAGAGGTCTTAATGGCAGCCCCTCCCATCACAAGCCCAGAGGCCTAAA

AGGAAAACATGGTTTCACGGGCCAGGCACAGGGCCGTGCTGCTTTGTGGA

GTCTCAGGACTTCGTGCCCTGCATACCAGCTGTGGCTCAAAGAAGCCAAA

GTACAGCTCAGGCTGTTGCTTCAGAGGGTGCAGGCCTTAGTGGCTTACAT

GTGGTGTTGGGCCTGGGGTTGGACAGAAGTCAAGAATTGAGGTTTGCAAC

CTCTGCCTAGAATTCAGAGGATGTATGGAAAAGCCTAGGTGTCCAGGCAG

AAGTTTGCTGCAGGGGCAGGGCCCTCATGGAAAACCTCTCTGCTGGGACA

GTGCAGAAGGAAAATGTGGGGTCGGAGCTCCACACAGAGTCCCCACTGGG

GCACTGCCTAGTGGAGCTGTGAGAAGAGGACCACCATCATCCAAACCCCA

GAATAGTCAGAAATGCTGATAGTTTGAACCATGCATCTAGAAAAGCTGCA

GATACTCAGTGCTAGCCATGATAGCAGCTGGGAGGGGGCTGTACCCTGCA

AAGCCACAAGGGCAGAGCTGCCCAAGGCCATGGGAGCCCACCTCTTGCAT

CAGCATGACCTGGATGTGAGACATGTAGTCAAAGGAGATCATTTGGGCAG

TTCAAAGTGTAATGACTGCCCTATTGGATTTAAGACTTGCATGTGGCCTG

TAACCCCTTTATTTTGGCCAATTTCTCCCATTCGGAACAGGTGTATTTAC

CCAATGCCTGTACCCCCCATTGTATCCTGGAAGTAACTAACTTGTTTTGG

ATTTTCAGGCTCATAGGCGGATGGGACTTGCTTTGCCTTAGATGAAACTT

TGGACTTGGACTTTTGGGTTAATGTTGGAATGAGTTAAGACTTTGGGTGA

CTGTTGGGAAGGCATTATTGTGTTTTGAAATGTGATGACATGAGATTTGG

GAGGGGCCAGGAGCAGAATGATATGTTTTGGCTGTGTCCCCACCCAAATC

TCACCTTGAATTGTAATCTCCAAAATCCCCAGGTGTCATGGGAGGGACCC

AGTGAGAGGTAATTGAATCATGGAGGCAGTTTCCCCCATGCTATTCTCAT

GATAGTGGGTGATTTCACATGAGATCTGATGGTTTTATAAGTGTCTGGCG

TTTCCCCTGCTGGCACTCATTCTCCCTCCTGCCACCCTGTGAAGAGGTGC

CTTCTGCCATGATTGTAAGTTTCCTGAGGCCTCCCCAGCCATGCAGAATG

GTGAATCAATTAAAACTGTTTTCTTCATAAATTACCCAGTCTCGGGTATT

TCTTCATAGCAGCATGAGAACAGACTAATACAACCAACATCCCCTCTTGT

CGATTTTCATTGAATCCCCATTACCTGGGAATGAATGAGGAAGGCAGAAA

TAGAAATCAGCGTATTGTTCAATATAGAAATCCTGAAGTTACACAAGATA

ATTACGAGCAAGACTCAGTACAGAGAAGGAACCCTTGAGCTGGGGATCAG

AGTCTTCTGGAAACAAGGAGTACCAAAGGGGAGTGAAAAAGAGAAGTGGA

GAATGAGTAGTAGCAGTAAAAGAGGAAGTATGTAAAGAAATGGAAATAAA

GATGAAATAAACTGTCATCTTGTTCCAGAGACAAAGCTAGCTAGGGGAGC

TAAGGAAATTTAGAGGGATAAAAAGTCTCATCCAAACCTTCATAATGAGC

TAAGGGCTAGTTTTTCATAGACCCTAGCCCAACTTGCAACCCTCTCACTC

TCTTACTTTTTTCCTGGCTCTGTTTTGCTTTACTGCCTTTCATATAATCT

AAAATGATGTTAAATGTATATTTCCTGTCTCTTTTCTATATCACAATATA

AATTCCATGAGGCCATTGCCTGTGCCACATTTACCACTGTACTCCCAGTG

CCTAGAATGGTGCCCAGATCAAGTAGACTCCCAGAGTGTCTGTTGTCAAA

TGATTCTCAGTCCAGTGATGTTTGCACTACATGGTGCTGCCACTTTTTTT

TTTTTTTTTACTTCATTCCATTTGGAATTTGGATCATTTTGCCAACTTTA

GGGTTTATTCCTCTTTATTTCCCCTGTGTTTGCTGAATATCTGACTATCT

ATTTTTCTCAGCTCTCTTCTCCTCACACTTTGCCTCCAACTTCCTCTCTG

CTACAAATTATACTTAGCACAAGACTGGAGCCACTTTTCATTGATTTTGA

TGTCAGTGCTTGAGTGACAACTCCATGTGGAGAACTTTCAACCTCCTGCT

GTCTCGTTGTTTGCTTTGAAAAAAAAAAATACTCATCTTCTTCCTGAACC

CACTGGGTAAATTTATGCTCACAAAAAAGACAAAACTTCTAGAACAAAAA

CTGTATGAGCTTCAAAAGTATTAGTTTTCCCTTGATTTTGAGACTCAAAA

GTACCATAGAAATTTTAAAAGGGGGATGAAGTAATTTTGTCATTCAACCC

GATATATTTTACAATTTGAATGTCAGAACCATGAAATGTCTTTTCAGTGT

ACTATCATCCTCACTATTTCCACCTATAAAAGAAAAGCAAAAATATTGTT

TGAGAACAAAAAAATATTGCAGTAGCGGAAAAATTCACTTTAAGTCTTTT

TTTGGAGGTTGCCACATCACGCCTGTCTTCAGCTCCTCTCACTGAAGATT

CTTTGTTTTCTTGAAACCAGTTGGTCACTTCCAATTCTTACCACCTATAA

ATCTTCCTGGTTCTTTATATTTCACTTTATATGTCCCTTATCTCCTCTGA

TTTTTGTTCCAGTCTTTCATTTTTTACATTCTTTGTCATCCTATATGTCA

GAAAGAATCTGCCACTCCAAATTCCTTAATATATTTATCTACCAGTGTTT

ATTTGCCTGTCACATGTACTCTTCAGTAATCCATTGACTTGAAAACTTTC

TACTCTGTCTTTCTTACTATAGTTTGCTTCTTGAGTTCAAATTATTAATA

TATTAGGTCAAGTAATTCTATTTTTATGCACATTTTACCTAACATTCTTG

GTAAATATAATTGTTTTTTAAATTGCATCACATGGTGAGTATTAATTTAA

GACGTAATTAGTATTAATCTACTGGACAAGATTTTATTTATTAACTGTTT

TGCCCTTTGCTAGGGTTCTGTAATCTCACCCTCAGTGCCTTTAAATAGTC

TTACCCAAGGATCACCTATCCCTCTTCAATTTAAATTCTCTATTGTGTTA

GTTTCTGAGATTGCACATTCTCTGGTTTGGTTTGGTTTTTCAGTGATCTC

TGTGGTCACTTCCAACCATTTCTACTTAGTTTACTTTTTTCCCTGTTAGC

CCTCTTCAGTGTTAATATCACCTGGAAGTTAACCCATTCCTAGTCTGCAT

TGCTCTATGGCCAGCATCTTGGCTGATTGGCCATCACCTATCCTGTACTT

ATTGTCAATTTTTTTAAATTTTTTATTGTGGCAAACAGCACATAACGGAA

AATTTACCATCTGTCTTTGTTCATGTATGTTGCTATAAAGGAATGCCAGA

GACTGGGCAATTTATAAAGAATGGAAGGTTTATTTGGCTCATGGTTCTGC

AGGCTGTACAAAAAAGCATGGCACCACTACCAACTTCTCATGAGGGCCTC

AGGCTTCTTCCATTCATGGTGAAGGGCAAAGGGGAGCTGGTGTGTAGAGA

TCACATGGTTAGAGAGCATAAACAAGAGAGAGAGGGGAAGTGCCAGGCTG

TTATTTGGCAACTAGCTCTTGCAGAACCTAACAGAATGAGCAGTCACTCA

ACCTGCCCCCAGGAAGGGCATTAAGCCATTCAAGAAGGATCCACCCCCAT

GACCCCACACTTCCCGTTAAGCCCCACCTCCAATATTGAGGATCAAATTT

CAACATGAGATTTGGAGGGGACAACATCTGAACTATAGCATTATCTTAAT

TCAAATAATTTTACTACCAATTCCATTTATTAGAGAATCTAAGATTAAGA

CTACTGAAACTATGCCAATGATTATCTCTAGTTCAGGCTTCTCTCTTCAA

TTCCAAACTCATATTTTCACCTGCTGACAAGCTACCACGTAGATGTTCCA

CAGGTATTTCTAACTCAGGATATGTAAAATTGATGTTATCATTATTTTCT

GAAAATCTATTCCTCTTACTATATTCCCTATTTTTGTGAATGACACAATC

TAATGGACTGGCCATGTTAAAAATCAGGAAGTTTTTCTGGCACTTTCTTT

ATTCCTTAATATTAAGCCCCACGATAAATTACCAATCCTTTCGATCTTGT

TAAGACAAATAAGCTTGAAATTTCTTATCTCTTATTTAGCACTGCAATTT

TGTTTTCTCATTCATTCAGATAGCATCTTCTACAAAACCTCTTCACTAGT

TTTTCTGTCTCTCAGCTCATCTTGTACACTGCAAATCCATTAAAACTACA

CTTTTTAACATGCCAATCATAATGTATTAGTAAAGGTTGTATAATAGTTC

TCTTTCTCTTCTAGGATAAAAGCTAAATTTTTCAGCTTGGTACACAAGGC

CGTTCATGGATTTGGTACCTACTAGTCTATCTAGGGTATTTCCTACATCT

TCCCTCCTCTCTCTTGAAAAATCAAGTAATACTAAACTATTTGTAACTCC

CTAATCACCAATTGCTGCCTTTATACTTTAACATATATTCTTCCTTTTGC

TAGAAATACATTTGTCATAATAGGCTCTTTGGTAAACTTACCATTACATT

CTCAGTTTATAATATTATTTGTGTAAGTACCTTTTTTCCTACTTAGTCCT

CCAGGGGAATGCATGTGCTTCTCTCTCCATATTATCATTTTAAAATTTAC

TCCTATTTCAGCAAGTATCATAATATTTTACTGAGATTAATTGTTGGCAT

ATTTATGTTACCCTGGTGACATATTTAATAGTTAAGATGCTTTGGGCTGC

AAGAAAGAGAATGCACTTTCAAAGTGGCTTCAAAAATAAGAATAATTCAA

CTCACATAACTGGAAGTTCAGAGGTTAAAGCTGGCTCCAGATGAAGTACA

AGCAGAGCTCTGTCACTCTTTCTCTATGACTTTCTTTGTCCTGATTTTTT

TCTCTGCGTCAGCTTTGACCTCCTCACTAGTTGCCCTCCAGGTTCCAAGA

TGACTGCCAGCAACAAATGGGTAACATAATTTCTTGTTACAGTGAGAGAC

ATAAACACTTACCTCACAGATTCAGAGATTTCACCCTCAGATAAGAACAA

TCAAAATTTTAATTGTGTATCTACTGTATCATTAGTATATGTATTAGGTA

TTCATATATATCTACTATTGTACTAAAGATGATTCAGAATGGTTTCATAA

GCTGGTTGATTTAAACTTTTCTTCCTGAACCAAGAGGATGAGATTATCTT

GACTGGCTTAGAATCGAAGATTTAGGTGAATCTAAATCCATCTCCAGAAC

TGAGGATGGATTCCGTAGAAACTTGAAGACAATTGGAATTCTCCTAGAAA

GCAGAATTTCAGAGTGGGTGTAGGGATATCTTTGAATGGAAGTTGTTTAA

GCCACCAACAATATTCACTCCATTGGGGTGACACTGGGCCCAAAGGTACC

CAGTTAATATTTGTTCAACGAACCTATAGGAAGACAATATTATCTGCAAG

CTATTTATAAACTAAATGACAACGAGAAGTATTTTTAGAAAAGTTAATCA

TATACAATTTAGTCATTCACATTGGGAGGCTCTTCCTAAATTTCGTTTCT

GATCCTTATCTTGATCTTTGACGCAATCATATCTTGGCCTCCATCTAAAA

GCCAGTAGAAGCCTCTGGCCATTTTTTAAACAATTGGTTTAACAACATTA

ATTGGAATCTATATAGCTCAACTTAAAAAAAAAAAAAAGAGGAACTAGCT

TATCATAATACAATATTGTCAAGGCAAGTGATACTTCCATGCAGCTATTC

AGCATTTGGTGTATTCTTGGTTTCTGCAGACCTAATAAAAAGAGATTATC

TCAGCGTCCTCTAGGTTTAATTTTAAAAAACAAGAAACCATGAACAATGA

AAATGATTTCAAAACATCTTCACTCACATCTAAAATATGAAATAGCTAGA

TCAGAGGTTCTTAATAATTTAATCGGTGAAGCATAACTGAAGGATTTAGA

AAAAGTGCCTAAACTATTAAAAGATTCTTCAGCTGCTGCTTAATGTTGAT

ATCAAGTTATTTTTTACACTGGCAAACAGCTAAGCCATTCCCTGGTCACA

TCCACTTTTCAGCATTTAGCGCTCCTCTCAATCATCCCGATCACAGCCCC

AGGAATGTTTCATGGCATCTCCGCAATAATAGTATATTACTATTGGGTCC

TGAATTTTGAGGTAGTTTATCTTTCAGAAAGGATATGAATAGAGCAATAA

GTCCAAGTAGAGTGGGCTTTATTAATGATGCCCAAGATTTTAGACGTATA

CTATAATCTTCAATCAATTTTAACACCACTTCCCTTACCCTAGTTTTAGT

CTAAACCAGTCCAACTGTGCTGTAGTCTCAGATAATACAATGTTAGATTT

TTTTTTTCAAGATATAGAATTGTCGGGAAAACTTGTATGTAACCTATACC

AATCTAAATTTCTTAGCATTTAACTCTAAAGAGTAAAGCTTTTAGCACTT

CCTGTGTAATACAAGGGCAAGTGCTGATTTCTCTGAATATAATTTTCCTT

TGTTAGCACATATGTTCACTGTTTAATAAAAATAAGAATGCTTCAATTAT

CTCCTTTTGTCAAGGGCCTGAGAAATAAAGAAATATACAGAGGGTCCCAA

TCTAAAAATGTTTTGATTTACAATGGTTCGGCTTAGGATATTTTTATTTT

ACAATGGTGTGAAAGTAATATGCACTCAGTAGAAATCATACTTCAAGTAC

TCATACAACTGTTCTGCTTTTCATGTTCAATACAGTATTCAATAAATTAC

ATGAGATGTTCAACACCTTTAATAAAATAGGCTTTGTGTTAGATGATTTT

GCCCAACTGTAGGCTAATGTAAGTGTTCTGACCATGTTGAAGGTAGGCTA

GGCTAAGCAATGATGTTCAGTAGGTTAGGTTTATTAAATGCATTTTTCAA

TTTATGATGTTTTCAACTTATGATGGGTTTTTCAATTTATGATGTTTTCA

ACGTATGATGGGTTTATCAGGAAATAATCCCATTGTACGTTGAGGAGCAC

CTGTATCAATATAGGCATTTACACAACTCTCGTACTGATAGCGGCAGGAG

GCAGAGAAGCTCTAGGCAGAAAAGGGATGGTCCCCAGCGAAAACCCCACC

CTCAAGCCAAAAAGCCTGAAACCGCAGCTCAAAGTGGGAACTTATATCCC

AGTTTTCCTGCTCGAATGTTGCCTTTTTCTAAACCACCCATGGCCCCACC

CCACCCCATCCTGTGCCTATAAAAACCCCAGACTCAGCTGGTAGACAGGA

CTACAGCTGGACATCAGAGAGAAGCAGCTTGACTTCAGAGGGACAACTTG

ATGGCATAACTTTAGAGAAGAATCCGGCTGGACTTCAGGGGAAGATTACT

TGCCACCCCCATCCCCTTTTCAGCTCCCCTTCCCACTGAGAGCCACTTTC

ATCGGCAGTACAATCCCTCACATTTACAATCCTTCAATTTGTTCATGTGA

CCTCATTTTCCCTAGATGCTGGACAAGAGCTCAGGAGCCACAAGTGTGAA

TACAAAAGGCCCTTTGCCCTTGCTGGTGGAGGGCAGCTGCCTCCTGTGAA

AAGACAAATGGCCCACTGAGCTGTTAACGCCTAAGCTGTCCGTGGATGGC

AGAGCTAACAGAGCACTGTAACACACCCTCTGGGGCTTCAGGGGTCGCAG

ACGCCTCCACCTAGATGCTGCTCCAGTGCTCATGCACTCCAGTTCCCACC

TCGTTTGCTTGCACACTCCCTCCAGTGAGGAGTTGAGAGCAGTGGGCTAA

GTAAATAAGGCACCCCTGTTGCGAGTTCCACAAAGGGGTCAGGGAAATAT

CCTGCTTCGTTACTAAGTAGGTATAAACCCTTTACTAAGTTAGTAATTAT

TCAGTAATAATACTTAAATGAAAGCTATTCTGTTAGACTAAATTCAGATT

ATCAAAGACACGACAGAAAACAGCTTTCTCATTCATGAACCTATTATTTC

CTTTTGTAAAATATGTTAAATGAAAATACAGCCGTCATGCTAATTTCTAA

TGGTAGAACATATTTTGAAAACTCCTTTATGTTTGGAAGATTTTGCTTTA

GTGCAGATAATCAGAATGATGTGATATACTAAGAAATAATTTTTAAAATG

AGATGTGACATTTCTCATAATCTAAATAAGAAATGGCAAAACATTGTCCT

AAGCTAAATAACTCATATGAAGTGATAAAATATTGCTTTCTAAAGGTCCA

TGATATGTAGTGATTTCTAATGTGTTTAATAGCATTGATCCTATTGGGTA

ATTGGGTGGTTCTAACATGTAAGGAAGGCCTCCAGTACTAATTTCATGTA

CTGGGAAACTACTGGGCAGGGATGAATCCCTTAACTCCTAAGTAAGACTA

TCAGATCATATAAATCTGCTTTTTGATTTGCAAAGACTCTTAGGCATACC

TCTTGAGAATATTAAACATCTACTAAATTATATGTAAAGCATTTCAGTCT

AAGATTTACAATGCTCAAAGGAGAAAGATTTTAAAGTTCAGCATTGGAAT

TTCCATAATTTCCTTCCAATTGTAGAATTTTACAATTGAGATAGCAAATA

ATAATATAAATAAATATGTAAGACTGAAATCAACATAGGAGTTTGGAAAG

AAGACTGTATGGGTTAACTAGAGTTGTCAACAAAGACTTCACAGAAGATT

GTGATCGTAGACATTAGTAAGGAATAAAATGGGTGCAAAAAAAAGTCTAA

GAAACAACAAGGATAAATTTATTTTGTAAAGAGTGAAAGCATATTGGGTA

TTACATAAAGAGACTGGCTAGTTTGACACAATGTGTACTTTTGGGGTAAG

CAGTGGGAAATCACTTTCTGTAAGTAAAGTGGAAGAGAATTCAGGTATTG

GATATGGTCATATAGGATTTTTCAACCTTTGTTTTGCCCCCATCCCTATC

CTACCAGTATCATCACAGATGTTGGCAATATTCACTCACTTGCCTGAAGA

TAGATATTCATGTCAGGGGCTACAGATATGTAAAGGTGAATGAAGTTAAA

TAGCAAAATTCAAGCACACTAGTTCTGACTGTGCACCCTCTCTCACTCAG

AAAAGCACTTTGGAATGCGAGAGTACTCTTATATTGACAACCTTTAGTTG

GTTCTAATTTATAAAAAAATATAAAAATATTTAGACAATTTGGATATTTT

ATATCTATGCCACAACCCATTGTCAAATGATTGAGATATCATTGTTTGGC

AACAATGAAACTGAAATCAACTCTAAGAGTAAAGAGTACGACTTTTAAAG

ATATTTGGTTAAGAGTAATGTTGCAAGGCAGAGACCAACATTATAGAAGT

TCTCAACCTGTGTGCTGGATATAGCTAAAATTTCCCAGGACCATTGAAAG

AAATTCTCAAATCCACAACCACCTCAAGTGTTGTCTGTGAATCAAAGGAG

TGATGCATTACCCACAAACATGTAGTAGACATTTTTTAAGTGATGTAGAT

GCTACTGCAATTAATAAATGCAAAATTATATTACAATATTACTGAATTCA

GAATAAGTTGATTTTGTCACTATTTTTCTCAATCTACGCATGCTAGATAT

AAAGTTATTATATAGAGACGTAACATTTTTAATATTTCAGAAGCATGCTC

ATGATATTGTAAACCAGGCACTGTTAAAAGATCACTGGATTGGGAATTAA

TAAACCTGGGATCTAGTCCTCATTTGATCTCTTGTTGGCTATGGTTTGGG

GGTTTTGGGCAAACTATTCAACCTTTTCTGCCTTGGTTTAATTGATAAAT

GATGGTGTTTTCCCAGATAAATCCCTTCCCAACTTAATATTACCAGATGT

AGCATCTATGGTTTAGAATGTACAGTATAAATTAACCTTCCTGAAGATCT

TCACAAGTTACTATAACCTATATTTTCATGGCACTGAAAACTAAGTTTTT

GATAGCTTACGTTTTTATAAATAATTTACTTACTCATTTTTATCATAATA

ATAAATCTGATTCACTAATCACCAAAATATCATTTTTGAAAATAGATGCA

TGAAAGGATCCGAACTTGTTATGGTTTTATCTGTTCAAGTCACCTAATTT

TGGTAGCCACAGGCCCCCATTGTCAATAGGGGAAGATTATCCATTTTAGC

AATACAGTCTCTGATAACTTCAGCCCATATGCCCTTCAATTCCACTTTTA

CGTCAGATTGAATGGGAATGTGAGGCCCCCAGTAGTAAACTAACCCTACT

TTCTCTTTGGAAATTGGTCTTTCTACCTAGCTCTTTGCCTCTGTTCACGT

TCTTGTATTCCACAGAAAATATATACATTAGGTGTTAAAATCACAATGAT

TAACAATTTTTAAGTAGAAATAGTTATTAAGTATAGCATAATCATGCCTT

TGAATTAGTACAAAAGTAGGAAACAGAGCTTTAGTGACTTTTTTCATTCT

TTCCACCATTTACAGGGCAAAAATGAAGAATTTTACCAATTCAAAACTAT

GCACATGTATAGTTTCCACCAGTATTTAGTAGTTATGTTTCTCAAGATGT

ATAATTCCTTTCCTTTCTGTTTTCTGTAGTTTGATAAACCCTAGATAGGA

GTTAATGTTGTTTCAGTTAGGTTTATTATTTCTTTTATGTGGTTTAATTT

CATGCAATAAGCTAGAGATTTTGTAACATAACTTGATAAAAATTTTCTCC

CTTCGTATCTTTTTGTTTTAAAATAATGGATTAATATAGATTGTAATTTT

ACAGTAGAGGAAATACATTTTACTTTTAGTTCTTATCTAGATATCTTAGG

AAAAAAGAAACATCATTTTTAAGGATTATTATTTTTCTACTAATGAAAAA

AATAGCATGATTTTCCATTCTGGACTTTGTAATTAACTTTACCTTGGAAT

AATTGAATCTTAAATATAACTTCATTGAAAATTTTATTTTCAAGTAACAT

TTTAAAATGCAAAATTTGTGTGATCTCTACTAAAAAGAACTCTCACATCC

CAATGTGCTTATAGCTACAATAATTGGTTAAAAGAGACAAATTATAAAGA

AGATATAGGTTGTGACATTGAAATTACAAAATGTGGATTAGGGAAGTAAG

CGTGTAGAGTTTTGTATGCAATCAAAGTTAAGTTATTACCAGCTTAAAAA

ACCCTTACGTAAGATGTAAAGAAAAGCAAAAACCTATAGTAGCAAAAGCA

AAAACCTATAGTAGATATACAAAAGGTAAAAAGTAAGGAATCAAAGTATA

CTACTGAGCAAAACAGTCAAACCATAAAAGAAGACAACAAAAAAAGGCAT

ATAGAACAAAGGATCTACAAAACAATTAGAAAACAACTTTTTAAATGGCA

GTAGTAAATTCCTACCTATCAATATTTACTTTGAATGTAAATGGATTAAA

TTCACCAGTCTAAAGATAGAGTGGCCAAATGGATTAAAACAACAAGACTC

AACTACATGCTGCCCATAAGAGACTCACGTCATCTTTTAGGACACATGTT

GACTGAAATAGAAGAGATAGAAAAAGATATTTTGGTTTCCATGCAAATGG

AAACCAAAAGAGAATGGGGATAGCCATACTTCTATTAGACAAAATAGGCT

TTAAAAATCAAAAACTAAAAAGACACAAAGAAGGTCATTAAATAATGATA

AAAGGATCAATTCATCAAGAAGGTATAACAATTGTAAATATATATGCACC

CAACATTGGAGTACCTAAATATATAAAGCAAATATAAAGTGATAAAAAGA

AAGAGACAAACTACAGTACAATAATAGTAGGGGACTTCTACCCCAAATTC

AACAATAGACAGAAAATCCACATTAAAAAATCAATAAGAATGCATTGGAC

TTTATGCTTTAGATCAAATAGACCTAGCAGACATATACAGTACATCTCAT

CTAACAGCAGGAGAATATACATTCTTATCAAGTGCACAAGAAACAATTCT

TCAGGGTAGATCATATGTTAGGCCACAAAATGAGTCCTAACAAATTTAAC

AAGATTGAAAGCATGTATTTTATGTAAAACAACGCCATCATGGAAAAAGT

ACTAGTGTAAACAAGGTTTAAATATGATTTACTGATTGTTTAAAAAGGAA

TTATCTTAGCCCTGATCTGATGGGATTTCCCCTTTGTAAGCAGCAAAAAT

AAGTTCATAATGAAGCAACTGTAATAATACAGCTTCACAGATCTTTCTGA

ATAAACAGAGTTGGATATGTTTCTACTTCAGAAACCATTTACTGTGGGCT

CACAGCTTTTCCATACACTCTTTACACTCTTAATTTTAAACCCATTCATC

AAAAGGATTAAGACAATGAGATTCAAGTCCAAGACAATAGGAAGTATGTG

CATCAAAACTGTCATGCTAATGCTCTGAGGAACATTGTTATTTCAATAGC

ATAATTTAAAACCACTGAAACCATGTTTTATTTATGATTACCTTTCATAC

GTTCAAAAAGAATTTGAGATGGTTTGGCAGGGATGTCTTAAAGAAACAAA

CACCAAATTTTTATTTGTTGTGCTTCTAACAAGCAATTTTTCCTACGTAA

GTGTTACCTGTTTTCTCCCCTTGATTTTGATCTCTTTTGTATGGTTGGTA

GTTGTCGCACTTCTGGGCATAGTATGGTTTTCTACAAGTTAAATAAAAAC

ATAAATTGATTTTGATGTAGCTAGTTCCACTATTTCAGTTAGTGTGTTTT

CCATACCTGCAATTCATAACATGTTTACTGACCCAGAACGTTAAAACTCA

AGTTTAGTTCAATACAGTCACTTCAAACCAACTTAAGATAGCAGTTACAT

TTCCAAGGTCATTATGAAGACGGAGTTCAAGACTTCAGCAGTTGAATTTG

TCAAGCTCATGGGTCTTTTAGTTACAGGAAGTGTGCATATTTCACATAAG

AAACAGCAACTCTGATGATACATTGAAACTCAAATATACCCAAGGAGTGT

AAAACTACTTTATAAGCCCTTAAACAATAAATATGCCAACAATCCTCTGC

ATACTTTTTGTCATTTTTTAGAGCATTCAATTGAATTATATAACATGTGA

TACCAATAAATAATTAACTTTTTATTTATTTATTTAGAGAATGATTCTTG

CTTTGTCTCCCAGGCTGGAGTGCAATGGCATGATCTCAGCTCACTGCAAC

CTCCACCTCTTAGTTTCAAGTGATTCTCCTGCCTCAGCCTCCCAAGTAGC

TGGGATTACAGCCATGCACCACCACGCCTGGCTAATTTTGTACTTTTAGT

AGAGACAGGGTTTCACCATAATGATCAGGCTGGCCTTGAACTCCGGACCT

CAGGTGTTCTACCACCTCAGCCTCCCAAAGTGCTGGTATTACAGGCATGA

GCCACAGCGCCTGGCTTATAATTAACTTTAAAAAATATTCTACTATCGAA

TGCCTGAAAAAATCATATTACTTCTATGTATTAAAAACAAACTATTACGT

AAATAGACCAGAGATACGGTGTCAGAGATGAGATTCCTTGGCAACAGTCT

CTGAGACAAAAAGTTGCACACAGAAAGTATTTTGAGAAGTATTGTTGCTA

TTTATAAGGAAGTGAAGGAGGCAGTATTGGGCAGAGAGAAAAGCTCATCC

ACACTGCAGTTGCTACTGAGGCTTCAGCCCATGTGACAGGGACACTGGAG

CTGGGATGGTCTTTCAGAGTTCTACCAAATTGAGGCAAATGGGCGAGACT

TTTGTATCTTTGCACTAGCCAAGAGCAGGCACCAGGGAGAAATGCAGCTG

TGATCCTTTTGTAGATATTATTATTCTGGAGTCAATGCAACCACACCACA

AATACTAGGAATAATATTGGTAGTGTGGGTGCATCGACTCCAGAAGAGGA

TCTTGATGAAGCATTACAGTATCCACTACAGAGAGGCACTGGCATATTCT

GCTTACCCCACAAATATTGCAATGAGGATGCAAGTAGGAGTAGTGAGGAC

ATAAAATAAATCGATTATTTTCCACTGGGCCTTAATATATCAGAACCATT

GGAATTTACAGGATGATATTTATTATAGTACTCAAAAAAATCTCTTTTAA

ATCTCCTTAACTCAGAAGGGAATTTTAAAAAGTCCACAATTCACCTGGTC

TATGAATTCCCTTTTAAAATAAAATGTGGTTTAATCAAATTTACAAGAAA

AAAACAAACAACCCCATCAAAAAGTGGGGGAAGGATATAAACAGACTCTT

CTCAAAAGAAGACATTTATGTGGCCAAAAGACACATGAAAAAAAGCTCAT

CATCACTGGTCATTAGAGAAATGCAAATCAAAACCGCAATGAGATACCAT

CTCACATCAGTTAGAAAGGCGATCATTAAAAAGTCAGGAAACAACAGATG

CTGGAGAGGATGTGGAGAAATAGGAACGCTTTTACACTGTTGGTGGGAGT

GTGAATTACTTCAACCATTGTGGAAGACCGTGTGGCGATTCCTCAAGGAT

CTACAACCAGAAATACCATTTAACTCAGCCATCCCATTACTGGGTATATA

CCCAAAGGATTATAAATCATTCTACTGTAAAGACACATGCACACGTACGT

TTGTTGCAGCACTGTTTACAATAGCAAAGACTTGGAACCAACCCAAATGT

CCATCAATAATAGACTGGATACAGAAAATGTGGCACATATACACCATAGA

ATACTATGCAGCCATAAAAAAGGATGCGTTCGTGTCCTTTGTAAGGACAT

GGATGAAGCTGGAAACCATCATTCTCAGCAAACTAACACAGGAACAGAAA

ACCAAACACCGCATGTTCTCACTCATAAGTGGGAGTTGAACGATGAGAAC

ACATGGAGATGGGGGAGGGGAACATCACACACCAGTTGGGGGATTGGGGG

GCAGAGGGAGGGATAACGTTAGGAGAAATACCTAATGTAGATGAAGGGTT

GATGGGTACAGCAAACCACCATGGCACGTGTATACCTACGTAGCAAACCT

GTACATTCTGCACATGTATCCCAGAACTTAAAGTATAATTTAAAAAATGT

GGTTGAAAAACAAAACCCACATAATACAAACTTTGCCAGCTTAACTGTTT

TATGTGTACAAACCAGTAGTGTTAACTATACATACATTGTTATTCAACAG

ATCTCTAGAATGTTTTCATCTCTCAAATCCGAAACTCCAAACCCACTGAA

GAGCTCCCATTGCTCCCTGTACCCCAGCACTGGCAATATGACCACTCTAC

TTTCTGTCTCTAAAGAGTTTAACTACTTTAGATACATCATATAAAAGGAA

GCATGCAGTATTTGTCATTTTATGACTGGCTTATTTCACTTAGCATAATG

TCTTCAAGGTACACCCATGTTGTAGCATAGGAAAGGATTTCCTTCTTTTT

TTGTGGCCGAGTAATAATATTCCGTTGTGTCCTATACCACATTTTTTAAT

CCGTTTATCAATCAATGGACATTTTAGTTACTTCAATTTTTGGCTATTGT

GAATTATGCCGCAGTTAATATGAGTGTGCAAATATCTCTTTAAGATCCTG

TTTTTAATTCTTTTGGATATACAGATGCTCATTGATTTACACTGGGATTA

CATCCCAATCAGCCCATCATAAGTTGAAAATATCATGTCAAAAATTAATT

TAATATGCCTAAGCTACCAAACATCATTGCTTAGTCTAGCATACTTTAAA

TGTTATCAGAACATGTAGATTACAGTACATCTGGGCAAAATCATCTTGCA

ACACAGTACATAGTAGAGTATCAATTATTTATCCTCATGATAAATATCAT

GCTGCAACCCAATATGTCAGAAGAGAGTATCATACTGCATATTTACTAGC

CCTAGAAAAGATCAAAATTCAAAATTTGAAGTATGGTTTCTTAATGAATG

CATATCGCTTTCACACCATTGTAAAGTCCAAAAATTACAAGTTAGACCTT

AGTAAGTTGGGGACTAACCGTACACCCAGAAATGGGATTGCTGGATTATA

TGATAATTCTATTTTTATTTTTTGAGGACTGGATTATATGATAATTCTAT

TTTTATGTTTTGAGGAACTTCCATACGGTTTTCCATAGTGGCTACACCTT

TTCACATTCCCACCAACAATGCAGAAGTGTTTCAGTTTCTCCACATCCTT

GCCAGCACATGTTATTTTCTGTTTATGATCGTGGTCATCCTCATGGTTGT

GAGGTAATATCTCATTGTGGTTTTCGTTCTTCATTTGCATTTCCCCGATG

ATTGACGATGTTGAGTATGTTTTCAGATGCTTGTTGGCTGTGTATATATT

TTCTTTGGAGAAATGTCTATTTAAATCCTTGCCCATTTTAAAATCAGGTT

AATTGTTTTTGGTGAATTCCTTATTAATTACTCACCTCAGAGCCTTTCTT

CTTAAAATACAGATTTCTCAGAACCTTTCATCTAATTTACTAAATGTTGA

TCTTGAAGAAGTATTTTAATTTATCTGAATTTAGTTTCCCGTTCTATAAA

TTGATAGTAATCATGTTTTCCCTATCCACACCAGAGTGGTATGATGAGGA

ACCAATGCAGAAAATGACTAAAAAGGCATTTTTATGCTGTCCAGATCTTG

TGCAAATATATTATAATTGATTTGAACCAAAAGAATCTACATTTTAAGAC

TATTTAATATTGTCGCATTTACATTGCACTAATGGTTCCTCTTTTTTCTC

ACTGATGAAGTTCTAGATTGAAACTCTGAGGAAACTGTAAATCACAGTTA

CATAACTCTGTTATATTAATTTATATAACTTCTCTGTCTCTCTCTAAATA

TATATATTTAGACTATATATACATATGCATATATATATTTAGAGAGAGAA

CGAGATTACACTTAAGGACTCTGCTTAGTACATTGCAACTTGCACCTCAT

TCTAATTGTGAAAACAACAACAAATTTTGATTGAGACCCTGTTATTTTTC

AGACCTTTGGTCTTGTGAGGAAGGAAAAGATGATTCTGATACTTATCTCA

AATAGCTTATCATCTCCTCTGGTGGATGAGGCTTGTGAGGGCCTGACACA

TGGTAGAGGATCTTATTCATTATATAACAACTTTCAAAACACATCACTAA

TTATTTTTCTGCTTAAGGTGAAAACGTAGCTCTCCCAAACAAATAAGAGA

TTTAGACTGAATTCTGTAGAAAACACTGCTTACCTCATTTTGCTTAGTTT

TCACTGCAGAGTCTTGTCTCTAACATTCAGGTAGGAGGAACTCTATTTTA

AAAAATTGAATAAACAAACCCAAATCAGAATAAATTATTTAATTCAGGAG

TTTTTTAAGGTGCTCAGAATTTTGGTAGAATAGCAGTGTATTTTCTGTAA

TAAGAACTACACAATGTTCTCTAGCTGGTTATACTCAAATGGTTATATGG

ATTTTATTCTGATATCAGTTTTGCAATTGATGACCACCCTTCATCAATAG

CGTGGTCTTTATTATGGTTTCATCTGGCTTTTTCTTCCCTTGTCTTCTGC

ACTGGGCTGCCTACTTCTTGGCTCTTATTATTACATACTCTACTTTTTTC

ATATACAAAGGTCTTAGTGCATTAATAGTGAGTTTCTGCTAAAAGTGTTA

CTTTCTTCAAATATCCTGTCAAATGCTGGCTGCCTGATTTATTGACCTCA

TAGAGTGACTATGTGAATCTTCCATTCTCTGTGGAATTCCATTCCACATT

TTAACTTAAAGACTCCGTCTTTTCCAGCTGTGTTCTCTCTGCTGGATAAG

CATTTTTCTTTTAATTTCTGTGGTAATGGGAAGGGAATTTTAAATCCTGT

TTTGCGTACAAGTGTTTAACAGGTAAAAATGGATGCTGTGTCAGCTATCT

ATTGCTGTGTAACAAGCTACCCCAAATGTAGAGACTTAAAATAAGAATCA

TTTTTTTTTTTTAGCTCATGATTCTTGATTCTGCTTGGGGCTTGGTTCAT

CGAGGCGATTCTTTTGCTAGACTCAGCTGTGCTCATTCATGCACTCGTGG

TCAGCTGGTAGTTGATGACCACATGTGGCATTCATATGTCTGGTATAACT

TTTTGGTTATAAGTTGAGACGGTGTGGGTGACAGGATCACTTGCCTGTAA

TTATTCATCAGGCTAGCATAGTTCCATAGCGGTGGAAGAGTTCCAAGCAT

AGCCCAACACCCAAGCATTGTTAAGCCTCTGCTTATGTCATGTTTGCTGA

TACCTCATTAGCCGAAACAAGTGACTTGACCAATCCATATTCAAGGAGTG

GAGAAACAGACTATGGCTCTTAATGGGAGAATCTGGAATATCTCTTGACA

AAAGTGTAGATGCAAGAGGAGAATCTGTGGACATTTACTCTCCAGCAGAG

ATGTTGTCATTTTTGTCACTAAGACAGTTCCTGTGTAATATTATTTATTA

TTCCTTGGCAGCCATAAGTTTGTGGTTTACTTATGGATACTTCAGTGCCT

GCCCAATATCATGTTGGAAAGAGAAGCCCTTAACAATTTCTCTTAATTTC

TACTTCTGAGGAATGAGAAAAAAAAAATTGGATAGGCATAAATGATTTCC

AGCACAAATTCTAAGCAGCTATTTGAGAGGTGGGTGGGTAGGGAGAGATG

GAAAATCCTTTTAATTGAGACATGCTCAACCATAAGCAATTTTTCTTTCC

TCAGGAGACATTTGGTAATGTCAGGAGACATTTTTGGTTGTACATCTAGG

AGGGAGCTCTGGCCAGAGATACTGACAAGCAATTCTGATCCTACAATGCA

CAGGACAGCCACTCACGACAAAAAATTATCTGGGACAAAAATGTCAATAG

TGCTGAGATTGAGAAACCCTGCTTTTCACTGAGCGCATGTAGATGAATTC

ATATAATGTTAGTTATAGAAGGGCACTTAGATGATGCCAACTAAAACAGG

AAAAGGCAATGATTATTTCTCTATTCAAGTTAGTGAAGGAAGGATATGTT

TAAAGTCAGGTTGGAGATATTTCTCAAGGGTAATATTTAAGTTGTGAGTG

GATGCCAGATGCGCTTGATTGTTACAATCCATATTTTTATAGCTATATCA

AATGATCTTCTCTCCCAAATTAAAATGGATATTAGATATATTTCATGGAT

ACCATATATTATAAGCTATAGCTCTTAGAAGTATTTGAGTGATTAATAAT

TCTTGCATATTGAATGAGACATGTAAGAAAGATACCTGATGTCCTATGAT

TCTTAGAAATATGTGCAGTGGTTAAGCACATTGTCTTGGAAACATCACTT

GTTAATGTGAGATCCTGAACAAGTTTCGTTACCTTGGTAAAAGGGGGATG

ATAACATCTACCCTGAAGATGGTTTGGTAAAGGTGAAATAAAAGAGGGTT

TATGAAGAACCTTGCCTAGCACATAAGAATCACTGAGTAAATGATGGGGG

TGATGGCAGTGTTGGTGGCAATTATCTTACTTGTAAGTAGTAGCAAATAT

TTATTTGGATTATATTTTTAATAGCAAGAAAGAATCTATTAAAATGTAAG

CAAATAGCACCATTAAGCTTTAAAAATCCATAGCTCCCAAACTTAACATT

TTTTTTTCTTAAATCCAGAAATACAAATGGTTATCATGAATCCTTGAGTT

CCAGTACTCCGGCCTTGCCTATGTAGAGTACAAGCATTTATTTTTCTAAA

ATGAGAATTATTGATGTTAGGTGTGTCATATATTTTATTTACTCTTTAGA

GTGACAGAAAAGAAAGCAGACAAAAATAAGTGTTTATGTGTTCATAGTGT

TTGTTTCTAATTTTCCAACTTGTATGTGCAGACTGTAAATATTTAGAGGG

AAGACATGAAATTCTTCCTTACCTACACAATAGATTGTCTTTCTTAGAAT

CTAGACATGATTTCAAATTTTCTTCAGAAGTTCTCTTGAATTCTTACGGA

ATTCATTAATCCAAATGCCATCCTTTCAAATATTCAAAAAGAAAATATTC

TCCAATGTTGCTTACATGGAGGAGAGTTATTTTATCTACTAGTGAGATGG

AAGTCGCTAACAATCTTTCTTATACCCCATAGTATTAAATAGTAATGCAA

TTTGATCACATATAACATTGCTCTCCTGTTGACATGGAATGATAAGGAGG

AAGGGAATATCTAACAGCACTTTTTAGATATTCTCTCATAGAATTGTTAA

TGTGAGCCCAATAACTTATTTTGTAAGTTGAGTGATTTTTCAAATATAAA

AGTGAAGCAAACAAAAAAAGTTACCCACATTCATTCAACAAAGCTAGAAA

TAGAGTATGTGCTTCTTGCTTTCTTATCAACCAAAGGAGACCTGTGAATT

ACAGGAAAACCATGTGTGAATACTTCAAAGGAAATATGCCAAACACATTG

TGTGTGTGTTTTAAAAGATTTAGTAGCTCCTTTAAAGCTCTAAGTAAAGC

TGTTAGTCTGACTAATCATGTACCCCTGAAACCAATTAAATTTTCAGACT

AGAGAAGTGTTCTTCAAACACTTGAAAAAAATAAGAGTTCCTTCCTAAAG

CCAACACCTTTTAATTAACAAAATACTCAGAGCAAAGCTGTGGTTTGCAA

TAGTATATTACACCTACTGTTATTCATGCTACCATTCTGGTGTGCCAAAC

TCAATTCATTTCAACTATTTTGAGCTTGATTTTCTGTCTGCTTAACTATG

GAGGGAAAACCACAATTTTAGTAGAGTCATTTTAATTTTCTAATGAATGC

ATGAGAAAGGTATTATGCTACATAGTATGTCCTCAGACTATTCAGATGCC

CTCTCTCTCTCTTGCTCTGTCTCTGTCTGTCTCTCTTTACACACACACAC

ACACACACACACACACACACAATGTGTGTATCTATCTGTCTACCTAGATA

TACAGTTTAATTTTGTACATTGTAATACATCTGGTTCTGGCATAAGGTGA

TTGAGCAAAAAACAAACAAACAAAACCCACCCAAATCTGTTACTGGAGGA

ACCTTTAATAGCATAACATCAGAAAGACTATTTTCAGTAAGGACCCTAGG

CAGGAAAAGACAATTCCTCCAATGGACTTGAGAAGAATACCCTTAGTCTC

TGAGGTGGTTAGAGAGGCTGAGATCTGAACCAGCTAATACCAGAGCCAGC

TTCTTGTAACCCAAGATAGGGGCATAAGAAACGGGGTGAATGCCCAGGCA

TGAACAGACTTGGGGTTAAGATTAAATAGGGTACACGGGAAAGGATTTCC

GCCAGCTGGAGGGGAAGCCATTTCCCATGCTCCTTAGGGATGACAACAGG

AGACCCAGCCCAGAACATCATTGAAAATTCTATGTAATAATTTATATATC

CTAACTCTTTACCCGTTCGTTCTAGATTATGGATTCAGGCACTCTTAGCA

AAACAAATCCTCTTTCAGTCTTTTAAAATATTGACTTAACAGGAAATATC

CTTGGCACAACAATACGACCCTGCCTGACCCTTAGAACTGTGAACAGGTT

GCTCAGACTGTACAAAACCACTAACAAATCTGACCAGCCTACACTTCACT

GAAAAAGGTGATTTGCATATATTTCTAGTACTATGACATGGTGGAAAAGA

AAGAACCGCATATGTGTTAGATAAAGGTAAACGCATCTGAAAGTGCAGTA

AGAATCAATGTGATATATACAATAAAATAAGATAAATAGGCCGGGCATGG

TGGCTCATGCCTGTAATCCCAGCACTTTGGGAGGCCGAGGCAGGCAGATC

ACGAGGTCAGGAGATCGAGACCATCCTGGCCAACATGGTGAAACCCCGTC

TCCACTAAAGTACAAAAAATTAGTTGGGCATGGTGGCAAATGCCTGTAGT

CCCAGATACTCAGGAGGCTGAGGCAGGGGAATCGCTTGAACCCGCGAGGC

AGAGATTGCAGTGAGCTGAGATCACGCCACTGCACTCCAGCCTGGCAACA

GAGCAACACTCTGTCTCAAAAAAAAAAAAAAAAAAAAAAAATTTCAGGAC

TTCAAAACTTCTGTCCTTTGAAACACTTTGATTAAAAAATGAAAAAAATA

TATACATGCCTGAATCCAGAGGCAAGTCTTTTTAGAAAACATAAAATATT

TTAAGTAATGTTTTTCTGAACAGAAATGGCTCTAATGTGAGAGGTCGATG

AAGATTAGCCTCTTGCTTCTTTATGTTTTAAAGTATATTCCTTGAGACCC

AAAGTTTCTGACTTTAGAATCCAACTCTCAACTACTATCAGCTATCCTCT

AGACACTTTCAAAATCCTCCAAAACCTAATAGTGGTGCAAGATTTTGTCC

TTCCTAGTGATAACTAGATTTAAGTAAGAAAACGTTAAAGACTAAAAAGT

GTTTAGGACCCACAAGAAAGGTGTAATTCTGTTCCTCATGGAGATATTTT

AGTTACACACTTCTTATTCTGGTCCTCCCCAAAACACAGAAATCTAGAGT

TGACATTTAAAGAGAAAATGAGATTTTTGTCTGGAAACAGGAGAGAAAAA

GTTTTCAAGAAGTATAGAAAGCGTATGAAAGGACACAGGTTTGAAATGGC

CTGTGCTCATGAGAGAATGCACAATAGACATGGGTGTGGAGGAGCCTGGA

AAGAATGGAGAGAGGTAAGTCTGGAGGGTCAAATCTGGTTCAGAGTACAG

AGCATAAAATCCCATACGAATGTAGAACATGAGGAGCCAATAACCATTGT

GTGAGAAATGCAGGAAAAAGAGGAAGAAAAAAATCCACATAGTGTACTTA

CAGTCAGAACAATCCTAGGAGGTGACTTACATGTATGATGTCATAAAATC

CGTAACACAATTCCATTAGATTGGTACTGTTTTCATAGTTACACAGGTGA

AAACTTTTGAGGGTCAAGATGTTCATTGTAGCAGGCATAGTATAATTCCT

TACCTCAAGATGCCTCCAATGTCAGTGAACATTACTTTTCCAAGATTGTC

TATTTAGGAAAAAGTAAGGCCAGTTGTCTATGAAGGAAGTACTATTATAA

TCGCCACTTTACAGAGGAGTAAATGGAGATTCAGAGAGGTTAAGTCATCT

GACTATAGTTACACAGCTAAAAAAAAAAAAAATTATAGAGCAAGGATTCA

AATACAACAATCTGGTCCTAGAAAGCCTAAATATTCTACACCCTGCTTTC

TGATTCAGAGCTATTTCCTGCTTTTCTCCCTTCGATAGGAAAAAAAATGT

ATGTGTGTGGGGTTGGGGGCAAATAAATTGACTGGCTGTATTTTCTCTCT

TTAATATTTGATCTTTCATTGTCTGCTCCAAGCAGTGGGTCTTCCTCCAT

TATCTTCTCGTGAAAATAGCTAAAATCACCTAACTAGTAAAAACCTAGGC

ATGTTTTGTGTTCTAGCCTCCATTCTTCCATTCTAATGGGTGGCTGTTAA

ATTTTAGTACTCATTGTATGTAGAAGACCTCTAAGTTCACTAAAGAGCTT

ACTGCACATTGACTTTTCTTTAGATTCAAAGGGAATCAATTGAGCACCTC

CTATACTCCAGGACATGTGCAAGATGCAAGAGATAGAGCAGTGAGAAAGT

CTGATAAGGTTTCCCCTCCCTTTCCCAGCAGTGTACAGATAGATGTTCTT

CAGGTGGTGCATGCTGGAGAAGAGAAGACCAGAAATACTGGCCTGACGGG

TCTATAGATGAGATTATCTGCCCATGACTGGCGCAGGGATGGCCGCATTG

CTAGACCTCTCCATCATCCTCTGAATATGCTTCAGCTAATTTATCATCCC

AATGGCATATTTAGTCATCATGAACCATTCTCTCTTTTTGAGTCTCAGGC

CCTGGCCTTGCTTTTCAATAGACTTCCAATAGATTCCTTCCTCCTTCCTT

CCCTTACTGCCTGGCTTCCTGACTTCCTTCCTTCCTGCCTTCCTGCCTTG

TCTTGCCTTGCTTTTCAATAGATTCCCTCCCTCCCTCCCTCCCTCCTTCC

TCCCTCCCTCCCTCCCTGACTTTCTTCTTTCCTTCTTCCTTTCTGTCTTT

TTCCAAACGTGCTTTTCAGGAAACAGTGGTCTGCTTGTTGAAGTCTGATA

ATTCTCTAGTTCCTCATCCTTCACTTTATTGAAATTTAGTGTGACATGAT

CATTTCCACCTCATTAGGTACTTCTTTCATGGTCAGAAGTAAGTCTGGAG

AAAAAAAAAAAATCTCTTGTCTCTGCTCTTATTTGAGGGTCAAATTTTCA

ATAAGTCACTTTTTAAAAAAGCATTTTCTGACACTTCCCATAAGCTTGAA

ATCCTCCTAAATTGCTGTATATTGCCTCAGTATACCTTGTGTATCTATTT

TAGGAACACTCCATCCACATTTGCCAGTCAGCCTGGTGTTCTGCAGTTAG

TTCCTACAGTGATATTTTAATTTAGCTCTCTTTTCATCCTCACACATGCA

TCCTCTCTGGATATTTAGCTCCTTTCCTGGAATCCCTTTTAAGATTTCTA

GATCTTTTTGCTTCCATGATTTCTTCTCCTGGACTGTGACACAAAATGCT

ATTTCTTCTTTACATTACATTTAATTCTTTCTAGAAAGAGCCTCAGAGTA

GTCAGATAGCTTTGAGAAACAAAACTTTTTCTTTATTGCCTCACTGTTAC

TGCCTTTCAATCATTGTTTCGTGACACAAATTTTTTTATTCTCTCTGACA

ATTAAAACACTATTTTTTTCTGTCTGCATTGATCAAAATTAGTTCCTTCA

TTCATAGAAAACTCTTGGTGTCCCTGAGAAGCTTGAGAGACAAGAAACAT

TCTTCCATTCTACTCATCTTCTTCTCTAATGAGGAGACAACCTTAAAAGC

ACAGTTACATAGCCATAAAAATTAATGATTGGCTACCTCAGAATGAAAAT

TCAATGTCTCATTTTTTTTTAATATTCTTAGAATCGTTCACTGGTTGTCC

AGTGTGAGTCTCCTGTTGAGATGTCTTTTGCAGCTTTCCTTGAAACCTTT

CATTCCAAACTACATAGTCCAATAATTTTGCCACCAATCTTCTGGTTATA

TTATGCTCTTGAGTCTGTTGTCTATAAACTTGATTAGGCATTCCTTCCCC

TCACCACTCACCTCTGATAACCCAGCTGTGTGTTGGTATTTAGTATCAAT

TCACACCAGCAAGTTCAGCCCTCTTCAATCAATATAGGGCCACACACGGA

CTTTTGACTGACTACTCCCCAAGTATTTCACATTTTGGGGCCTTATCTCC

AGTTTCTCACCACAGTTGTTCATCACTGTGTTTCTTACTAGCCAGGCGTT

TATAAAAACAGTAATACCTAACACTATTGATCACCTACTATAGTGTCAGG

CGCTGTAATAATATTATTGTGATGATGATGATTATGCTGCTCTTTCTGGC

ATTGTCATACGTGTATTGCTTGTACTACTCACTGAATCTACACAACTGCC

CTTATGACATTTACCCTGTTATTATTCCTCTTTTAAGGTAAATACATGAA

AAATGCTTCCCACTTTGCCTTGCTTACTGCTTATTGCTAGTACTGAACAA

ATGTTAGAACTGAAACTTAGAGAGGTTATGTGGCTTTACCAAGGTCCCAG

AGTTCCTAGGGCAGAGAACAGGATTGTCTACCAGACATTTTAATTCTAGT

ACTATGCATCTTAACCATTACCATAGGCTGACTTACTCTACAGTGTCCAA

CACTATTCATATTAAGATTTATTTAATGACTTTGAAACAGTATTTCATGT

CTAAATAGAAAAACTACTAACTCGCATTTTTAAGAAAATATTGTATCTTG

GTTTTTCTTCACTGCTGGCCAGTTTACTAACAATCTGAAATAAAAAGAAA

AAAATATGATAAACTGCTCCCAGTATAAAATACAGAGCTAAGACAAGAAC

GTTTCATTGGCTTTGATTTCCCTAGGGTCCAGCTTCAAATTAATTTACTT

CCTATTCAAGGGAATTTTAAATCAGAAAGAAGATCTTATCCCATCTTGTT

TTGCCTTTGTTTTTTCTTGAATAAAAAAAAAATAAGTAAAATTTATTTCC

CTGGCAAGGTCTGAAAACTTTTGTTTTCTTTACCACTTCCACAATGTATA

TGATTGTTACTGAGAAGGCTTATTTAACTTAAGTTACTTGTCCAGGCATG

AGAATGAGCAAAATCGTTTTTTAAAAAATTGTTAAATGTATATTAATGAA

AAGGTTGAATCTTTTCATTTTCTACCATGTATTGCTAAACAAAGTATCCA

CATTGTTAGAAAAAGATATATAATGTCATGAATAAGAGTTTGGCTCAAAT

TGTTACTCTTCAATTAAATTTGACTTATTGTTATTGAAATTGGCTCTTTA

GCTTGTGTTTCTAATTTTTCTTTTTCTTCTTTTTTCCTTTTTGCAAAAAC

CCAAAATATTTTAG

45782 BP

INTRON 52 full sequence

GTAAGTTTTTTAACAAGCATGGGA

CACACAAAGCAAGATGCATGACAAGTTTCAATAAAAACTTAAGTTCATAT

ATCCCCCTCACATTTATAAAAATAATGTGAAATAATTGTAAATGATAACA

ATTGTGCTGAGATTTTCAGTCCATAATGTTACCTTTTAATAAATGAATGT

AATTCCATTGAATAGAAGAAATACATTTTTAAATCAATTCAGGGCTTATA

TAGTTGCAAAGCATGCATTGATGGGTGTGGTGACCACAGTGTGGCAGAAC

ATTTGTGGCAGAACATTTGTTCTTTAGTTGTCATCTGGGCTGGCATCCAT

GGAGATGCCAGTCTCTCCCTCATATCCTTGGCTGTTGGTCCAAGCAGGCA

GTGGCTTCTTCCTGGGCCATCTTTCATTCCCATGTGCAGTGACTTTCAGA

TCTGGATATCTCTCCGCTACTTTGATGCCCCCATTTTGTAATATCAAAAA

TCATCGTACTGTACCTTATGCCGTAGTAGGGTGGGCAGGAACTTTGGTAA

GACCCATCTGACTAGACGCTGTGCATATTCTTTTCTTCTGACATACACTC

CTATCCATTTAATGGGGAGAGTGATTCGCAGTGATTGTGTGTTGTGTCAG

TGAGTTTCCATGGGGTCAGGAAGAGTGACAGACGAAGGAGTAGGGGAAAC

TCGCCACCCGGTTTCCCTCAGAGATTCTCCTCAGAAATGAGGTCCAAGTC

AGCTCTGCTTTCAGGTTCTCTCAGATCTCTCTCGGTTTCTTCACTTCTCT

CTACCTTCCTTCCCTCCAGGGACACACACTGGTATTGAATTTTCTTGCTT

CCTCTGCAATATCCCCTCATTTTCCTTCCCACAACCCGAAGAATCCTTTG

TAGTGCAGGAAGGAGGAAAACCTTTCAGCCATCTTTTTTTTTCTCTTTGA

AATCTTTTGTCTTTTACCAGGCTTAGACTTTTCAAACTCGGAAACCATGA

GAGTCTATATCTTCATAATTTATATTCTGCTATGTTAACCCTTCCCTAAG

GAAATGACTAGTTGTCAATATGTTGGGGAAAGTGAAAGAGTAACCAGAGT

AAAAGGTTAATATTTTAAAATATTATTAGTCATACTTCCACATATTGGGT

AAGTACTTATTGATAATAGCTAGTATTTATTCAGTACCTCATAAGCATTA

GATGGTGTGTGCACATGTGTGTGTTTCTGTGTGTTTCTGTGTGTGTGTAC

AAAATCTTTACAGAATCTTGTGAGCTATATTTTATAATCCCCATTTTATA

GACGAGAAAACAGGTTCCAAGAACAGTTACTCGGCCGGGCGCGGTGGCTC

ACGCCTGTAATCCCAGCACTTGGGGAGGCCGAGGCGGGCGGATCACGAGG

TCAGGAGATCGAGACCATCCTGGCTAACACGGTGAAACCCCGTCTCTACT

AAAAATACAAAAAAAAATTAGCCGGGCGTGGTGGCGGGCGCCTGTAGTCC

CAGCTACTTGGGAGGCTGAGGCAGGAGAATGGCGTGAACCCGGGAGGCGG

AGCTTGCAGTGAGCCGAGATTGCGCCACTGCACTCCAGCCTGGGCGACAG

AGCGAGACTGCGTCGTTTACAAACAAGCAAAGAACAGAGCTTGGAATTTG

TAAATGTCTCTTTCTGACTTGAAAATCTGCGCTCTTTCTAGTATGTTTAC

CATTTCCCATCTTGTTTTGTTGCTTTTGTTAATGACCTTAATCATTGTAC

TAAGACTAAATACTTCTTTTGTCTGAAATTATGTATGTTTTGATTCACTT

CCTAAAGACATGTCTTCTTTCAGTTGTAGTGATTGCTAATTAAAATAGGC

TGTTCTTGGTTTTGAAAGTTTAACTCTTTATTGTTGCTTAAACAATGAAT

GTGGATGTATGCTAATGTATTATTTCAGTAACACTAGCCACTATAACAGG

TAATCTCCCAAATCTTGGAGGCTTACTACAGCAGAAATTTACTTCTTATT

TTAGTGCAGTCCAAAATAAGCAGCCCTCTATGAAGTCATTCAGGAACCCA

AGCTCCTTCCATCTTTTGTGTCCAGAGCATAAGACTCATATGCATTTAAT

GGGCAGATGAAGAATGAGTATTACTCATGAAAATTTGTCATGAGCCACGC

CTGGAAATGGTGTGTGTTAATTTTGTTCATATTCTGTTGGATGGATCTTG

TCACATGGTCACACCTAACTGCGAGGGAATTAGGGAAATAGTGTACACTG

TTGAGCCTAGGAAGAGGAACAGATTTGGTAAGATAGCCATACAATTGATT

TAGCAGAGCATTCCTCCACCATACTGGAACCTTGAGGGTTCTCCAACAAG

TTGCAACACACTGACCCAAAAGAGTGAATCTTAGTGAGTGATTTACTCAA

TATGAGACTGAATTCCCATTACACAGGTAAGTGATCAGTTTCTTGCCTGA

GAAATATGGAAATTTGGCAGTGAGGTTTATGCAAATCTGAACATACTATA

CAGAGAGTCATTTGTTATTTTACTAATGAAAAATCACTTCAATTTTTTCC

CTAAGAGGAAGACAATATGAATGTATTATACAGTATTTGTTCAACATTGT

CTGAACATTTTCCATTATCCCCTGCATTTTTTTTTCATATTGCATTGACT

TTTTCATGATAGAGATTAAAATTGAATGACCGGAGGGCAAGTTTGTATCT

CTCTGCCAATATTCAGTGATTTGAATAGTTCTTCTTTTCTAAGCTTTTGT

CTTTTAGGAATGAATACCTTTATGAATATTTGCAGCCTAGTGGAAAGGCT

GTAATCCAAATGGTCCAAGAAAGCATTTTCTTAAAAGCAAGTGTCTGTCG

AGATGTGTCATAGCGCTTATTAAGAGTCTAAGCTGGAATCTTAGTTCCAA

ATATGCCTGGAGCCTCTAAATGGTACCAGGATAATTCTGACAAGATATGT

TTATCTAAACAATGTCATTTGCAGCCCTGCTACACTTCAGTTTATCTCTC

CCTTTGAAATCTATAAAATGGGATGGAAATTCAATACTCATAAACTTTTC

GATCGTGTTCAAAATAGAATTTTTCTTAGTAAAGATTTGGTTTTCAGGAA

AAGGACAGAAATAAAATACTTGCTCATAAAATTGTATTTTCTCATTTGAT

GATTTTGGTCTTCCTTTTTATTGCCATGAAACTTCTAGAAATGCTCAAAA

AGAAATCAGCTAAATAAAGAAAAAATAGTTAATATATGTATGTAATACTA

TATTGAAACATTTTTCTTTCTCTGGTAAATCCCATTTCATAACTTTGAAC

AGTTGGGAAAATCTATACATAGTTATTGCAGTCTATCAAGAGAAAAGTTC

AGTACAAAGCTATTTATGTCTACTAGAAATATTCATGTTAAACTTCAAGT

AATTGGGTGTGCAAGCCACCACCATGTTTTACTATATGAAACTATTACCG

TGGTATCTGTTGTATTCAGGTAATTATATTGATGGAAATCATGCAGTAAT

AATCTAGGTAAGAGAGTAAATTTTGTCTAAATCAGATCAAATGAAAAATT

CTCCCTCTTTCTAATATTCGAATTGCTCATTTTTCTTTAACTCTTTGGTG

TCTGAATTTGTCAATCATTCCTGGCCATTTTCTTCTGCAAAAGGGCTGGG

TCAGGGGACCAAAAGCAGATAAGATTAGAAGAATTTAAATTTTCTTCCTT

GGAGGCGTCTGAATTACATGAAACTCTTGTTCGTGTCTGTTAATACTGCA

AGGCATAATACCATAATACCTTGCATAGCAGTGAAGAGGATTTGGAAAGA

TAAAACTGCTTCCTTTTATCATTCTGTTTATTTCACAAACAATATTGGTG

AATGTCGTTCCTGTAACATTTGGATTTAAGAGCCTTGTTTCTGTAGCTTC

TCCCTCCGTAACCCCCACCACTACCATTTCGGGGCTATACAGCAATAGCA

TGCATTACTTTAAAAGGCAGGCTGCCTAGACTGGCCACTTGTTAGCTTTG

TGGCCTTGAGCAAATGACTAATCTCAGTAAACTATCTGCTCTTAGTTTCC

TTCTCTGTAAAATAGGCTCACTTATAACTATCTCATGGGTTGGGAGGATT

AGATGAAATAATTAATGTAGAGCCCTTAGATCAGGGCCATAGTAAAAGCT

GAATGAATGTTAGCATTTGTTATTTTAATTATAATCTATTGGGGTGCTTT

GAAGGCTTAATGCAAAATACTTAATGAGCTTTTTGGTAGCTGTTTAGTTA

TTTCGCCCCCCACCACCACCCCAAAAGGAGAGATTTAAAAGACCGACAGG

AGAAGGTTGCTTGGAAAAGATGGAATAAGATCTATAAATAGAATTAAACA

AATATTCAGGAAAGCCTTTTGTGGGAAATACTGCAAAATTTTTATTATCT

ATAAATTTAATAGGTAGATAAAATTACTACTCCCATTTTAGAGACAGAAA

ACCGAGACTCGGAGAGCTAACGTAACTTGTCTAGGGTCTTAGGAAGATGA

CAAGTGAGGAAGTAGAATTCAAGCCCACGTCTATATGATTTTAAAGCCCG

AGGCACATCAAATGGAAAAGGCTGGTTAGTCAGAAAAATAGGAAGGTATA

TTTATCTGACAACTTAAAATATTAGGACTAACCTCAGGTAATTATAGTCT

GGATATACATTTTTGCTGCTCCTGTTTATACTTTTGACTTCTGTGTATTT

GAGTGTCTAATCAAAGGATTGTCTTTTACATGTGTTGGAGATGTACAGAC

TAGTGGACCCCAATGATCTATTAGCTGTGTAACCTTAGCCAAGTTAATTC

TCTTTCCTAAACTGTGGTTCTCTCATCTGTCATGTGGGGCTAATAATAGT

ACTTATGCTGGTAGGGTGATTAAGAAAGTAAAATAATTGGTGTTTCTAAA

GTAAATATGTGGCACATATTAGATGCTCATTAAATGGTACATATTGTTAT

GGTGAGATGGATTTGGTACAGAGAGAACTGGAGATGGGAGAATATGAAGG

GTGTATAATGTGGCCTTTTATTAGCTAAACCAAGGGAAGGACTTCTGAAA

CAGAATTCCAAGTTTTAAGAGGGAGTCGTTTATTTTGGAATTATTTTTTC

AGCTAAGGATTTTTCAACCCAGTCCAGAATTCTTAGAGAAATTTAGTGAT

AGCTTATAAATTTTAAGAAAAGGAATTCACATTATATTGCATAAAGAACT

GGTATACAGGGCCATAGAAGGGGAGAATGTTCTTCTGTATGAGAATAAAA

AAAAACATCTCTCACACGATTTTTGAATTAACTGACAGTTTTATAGCAGC

TTTGTCAACCCATCATTCATTGCTGCAACCAAATCTATGAAATCCTTCAT

GGCGAAATAAAAAGGCTCTGTTGTTCTCCACATTTGTATGAAATCTCTGT

TGCTAATGAAATGCCAGCCAGTATCTTCCTCTCAGGTATTGTCTATTAGA

TGGTTGCTTATTTTAGAAGAAGTGGAGTCAACCATATAAATTTCCTTCTT

TTGACATCTAGCACCTGCTGTCAACCTGTTATAGCTACAAGCAGCTCTCA

AAATTCACATCCACTAGGATGCCGCTGGCAACCAAAGAGTTCAGTTCAGT

TCAGCGAACGTTTGAATGCCTACTCTGTGCTATCTAATATCAGAGATGGT

AGAGGGGATACAGGAAAAAAGTAAGATTCAGCCTTTGTCTTTAAAGAGCT

CACAATCAAATGTGGGTATTTGGACAAGTATATTTAGGCAAGGCAGTTTA

GGATAGGTGCTTCAGTAGAGCAGTATTACAAAATGTTGAGAGAAAACTAG

AGGAGGAGTTTTAAATGAGGGCTTAGTGGAATGCTTCCAGGAGGAGGCTG

AATTTGACCTGGTCATGAATAAGACTTTGAAAAGCAGAAGGAAGCTGGAG

AGGGAAGGGTATTTCAGGAATTGATGACAGGAGAATACATAATTAGACCT

GTTAACAGTGGGGTGGAAGACGAATATCAGCATGGGAGTGTGTGTGTGTG

TGTGTGTGTGTGTGTGTGTGTGTGTGTGTGTGTTGTGGAGGATAAGGGAG

TAAATGAAGTCAGTAAGACCAGTTAGAAGGCAAGAGAGCCCAAGCTGGAG

CAGTGACCATGGAGTAAAAAGAAAGAAGTGAAATTGAGACATAAGGTGGA

GCTAGAATTAACAGGATTTTGACATTGATTGCATATATAAAACTGTCAAT

CTAAGAGAAGGCCTGCCTCTAAGAGTTGGAGATTGAATTCTTTGGAAGAT

TAATGTCATAACCAGAGACAAACTCAGGATAAGGAGTTGATTATGGGAGA

CAAAGTAATGTGCTTAACATGAAGCTAGATGACCATACTGAGATTTTAAA

GAAATGGTTGAAAACATAGAGATTGCAGGAGTGATGTTACTTTTGGACAA

GAATTCAAAAGTTACCTGGAATGTCTGAGCTTGCACAGAGAGAGGGTACC

TGGTGCAAAGAAAAGTGAACCTAGGAAAAAGCCTAGAAGTTGGTTTACAT

TTAAGGAATTTGAGAAGAGGTAGCACCACCCACCCCCCACACAACCCCCA

ACCCTGCCAACTTACAATATAGAAGCATTTAGACACACACTGAATAATAA

TTTTTTTTTTGAGACAGAGTCCTGCTCCATTGCCCAGGCTGGAGTGCAGT

GGTGCCATCTTTGCTCACTGCAACTCCGCCTCCCAGATTCAAGCTATTCT

CTTGCCTCAGCCTACCGAGTAGCTGGCATTACAGGCTCCCACCATCATGC

CCAGCTAATTTTTTTGTATTTTTAGTAGGGACGGGGTTTCGTCATGTTGG

CCAGGCTGGTCTCAAACTCTTGACTTCAGGTGATCCACCCGCCTCGGCCT

CCCAAAGTGCTGGGATTACAGACCTGAGCCACCGCGCCCAGCCTAAATAA

TGAATTTATAGATGCTACACTGTATGGTTTCCTTTTTCTGCTGCTGTACA

ACCATTCAAGTAACATAAGTTTCATCCTGGTTCTTAATGATACCATGAAT

AAAGTATAGAAACTCTTTAGCTGAGGATTAAAGATTGTTGTGTTTAGGTA

GACCAGGTTTCTGATGCAGCCCTCAGTATACCAGGGAAATTAGCCATTAC

TGTTTGCCTTGTAGGTCTAATGAACCCTTTAGCTTTTTATTTTGTATATG

TCTAAGTTACTCTACAAATTCTTATGGAAGTATAAAGAGATAGTGAAAGA

CAATCTTATGAGAAATTTTAATAAGAATTGAAATACAGGCTGGGCACAGT

GGCTCACTCCTGTAATTCCAGCACTTTGGGAGGCTGAAGCGATGGATCAC

CTGAGGTCAGGAGTTCGAAACCAGCCTGGCTAACATGGTGAAGCCCCATC

TCTACTAAAAATACAAAAAAATTAGCCAGGCATGGTGGCGGGCACCTGTA

ATCCCAGCTACTCGGGAGGCTGAGGCAGGAGAATTGCTTGAACCCGGGAG

GCGGAGGTTGCACTGAGCCGAGATCACGCCATTGCACTCTAACCTGGGCA

ACAAGAGTGAAACTCCATCGTAAATAAATAAATAAATAAATAATAAAAAA

GAAATACAACTTACTTTTTGTATCAAATAAATTTTGGTGCACAAACATCA

TAAATGACATAATATCTGTCACACATCCTTTAGCAAAACAGGATTGTTAA

AATTCTTACAGCTAATATTATGTGGTGAAGTTCTTCCTGTTGAATAATTA

AGTGGATTAAAATATCATATTTCCTTTCTGTCATTAGAAATATATTGTGC

ATTAATCAGTCCTTGGGCCTGAGAACATTTATCTAGGTTTTCATCATCTG

AAAACTCACAGTCCAATATTTCACCTGTATTATCAATTCACCATTATCAT

TAGAGTTTAGAATGCTGCTGTCTCTTTCCATCTATCTTCTGTTGTATCTA

ATAATTAGGAGATATATATATATATATATATATATATAGAGAGAGAGAGA

GAGAGAGAGAGAGAGAGAGAGTGAGAGAGAGAGAGAGATTGGTAATAATA

CTAATATATATAATAATATTATATATATACACACATACAGAGAGAGAGAG

AGAGAGAGAGAGAGAGATTGATTTTAAGCCATTAACTCATATGATTGTGG

GAGCTGAAAAGTCAAAAATCTGTAAAATCCAAAAGCCTGGCAGGATGGAG

ACCAAGGGAAGAGTTGATGTTGCAATCTTGAGTCCAAAGGTAATCCGATA

GCAGAATTCCTTCCTTTTCTGGGGACTTCAGTCTTTTCTCTTAAGGACTT

CAAAATTGATTGGAGAAGGCCCACCCACATTGTGGAGGGTAATCTGCTTT

ACTCAAAGTCTGCTGATTGAAATGTTAATCACATCTAAAAAATACCTTCA

CAGCAACATCTAGATTGGTGTTTGACCAAACAACTGGGAACCATAGCCCA

GCCAAGTTAACACATAAAATTTACCATCATCAGCCATCATCATGAGGTTC

ATAATGTCATGTGTTTCTTCACATATAAATCTCGTGGTTCTTTTATTTTT

TTAGTAGTACCTAGTTAATAAGGTACCTGGAAATATACGTTAAATAATAG

AAATGGTCTTTAATATCTTAATGAATTTCAGAGTTCTATCAGAGTAATTG

TAACTTGTATATAAAAACTGATATATAAAAAACAAGTATGTTTTTTCTTC

CTATACGTTGTGTTAAATTAATACTAATTACCTATCATGAAATGAATGTT

TTCCATTTTTCATAAATGTATTTCATTTTTAAATTAAGAGGATTAATATC

AAAACATATTTGAATAGGATTTAAAATCCAAATTACCTGTTGCGATTATA

TTTAATTATAAATCTTTCAAGTGAGTTTATAATAGGAAAAATATTTTTTA

TTAAAGTGATGTACCATAATTATGAACATAAATAGTGTTTTCTACTAATT

GTTTTTGTCTTCATTATGTTTCTTTTTAAGGGGAAAGATCTGGGTTAACA

AATGTTTTATTCATCCACCTTGAAAATTAAACATAATAAACAAAACTATT

AAATAAATAAACCAATTAAAGAAAATAGTTTTTCTTCCTTTTTTGTGATA

AAGATGATTATGAGTAAATATATTAAAGAATTTTATACTCAGACAAAGTG

AGCTAATAAGACAAATACAAACAGAGATGAGCCCAGAAAGAGATACTCTA

CATGTTTATAGGCTAGCATTTTTATCTTATGACCTACAGGGTCATTGTAA

CTAAAGTCTTGAATTTCTGTTATATTTTGGTACCTGTGGGCATAAATAGG

AATATTACTCCCTGATGGCATCATGCATTTTAGATAAAACTAAGCCAAGG

GTTAAACACGTGCCATGGACTGTTCCTAAAAAATGCTCTCAGCTCTCAAG

TTATAGAAAATTGTAGGGATTATTATTTTGTACCTACTACTTCTTTCTTT

ACCAAAATTTACTTATACATGATTTGAAAATTGCTTGCCACCTGTCTTTA

TTCTGTTGTACTTGTCTTAAGTAAAACATTTATAAAGTAAGTAGGAAGGA

TTAATAGATTGTGACCTTTCTACATGAAAAGGGGAAAGCCGAGCTTGTTC

TACTTTTGTCAGGAAAGAGTTTGAATAGTTACCTGTTACTGTAAAACAAT

CACCCCAAACTTAGTGTCCTAAAGTAGCAATAATCATTTATTATCACTCA

TAGGTTCTGTAGGTCAGGATTCATGCATTGCTTGGTTGAGGGGTTCTGTC

TCACGATCACTCATGATGTTGCACTCAGATGTCAATTAGGCTGCAGTCAT

ATGATGGCTTGACTGGGGCTGAAGGATTCACTTTCACAGTGAGTTACTTG

TATAGCTTGTGAGTTGGTGCTGTCTAGTTGGTTTCCCTCTAAACGAGTTC

CTCCATGGGACTACTTGAGTGTTCTTATGATGTGGTGGCTGGCTTACCCC

AGGACAAGCAACCTATGAGTTTTGCCCTGAGTTTTCTGAAACATGATCCC

ATGTTGCTTTGAATATCCCCATTAGACACTCCCACAAATGTCAATCATAC

TCTTTGCCCTAAAAGTTGCAGACTACTTGTACATGTCCTTTATCCTACAT

GTGTACAGGAAACACGTGCAGAATAACATGTGTTTTTGTTTCTTGAAAGA

AACAAGAACCCACCAAAACTTTATAGCTCCGGGACATGATGATAATTATC

TCAGGTCCACTCAGCCTGGCTCCACATGGAAAATATGGGTATTGTAAAGA

AAGCTTTTGAATTTTGGAGGCCTAACAAAAAGACCTAGGAAGCTAATAGT

TGGTTATGAGATAATTATAACAATGGTGATGAAAGCAATTGCGATTATTA

CTTTTATGTTAGTGAGATAAATACATAAGAATGTCCTTCTTTAATTTCAT

TCCTTAATTTCCTTAATCCCCATTCTTGATTTCTGGATTAGAGGAAAGCT

ATACTATCTACCTGATTACACAAAAAGACTGGACTGAGGGGTAACCATTA

GGACTCTTGTTAGTTGAAGGTAATAAACTATATTACTTCAGTGGCTAAAA

CAAACAGGAGCTTTTTTCTCATATAAAACTAGAAGTCAGGAAACAAATGG

TTGCTGATGTTGGTTTATCCGTTTGACAGGTTCTACCTTTATCCTGGAGA

TTCTTTGGCCTTTATTTCTTTAGTAATATAGTAGTAACAGCACTGAATCT

AAAATAAGAGCACTTGGTCCAAGCACTGGCTCTGCCCCCTTAGTGACAGT

GGGACCCATGGCAAATTATTTAATCTCTCCAACAGTTAATTTTCTCATCT

GTAAAACTGGGATCCTGATAACTTCCATACTAGTTTGGTGTTAGGATTCA

ATGAAATGACGCATGGAAAAGTCCTTTTCAAATGGCTACTCACTATACAA

ATATTAATGTCTTAGTAAGCTCAGGCTGCTATAAAATAATGCCATAGACT

GGGTGGCTTAAACAACAGGCAGTTGTTTTTCACGGTTCTGGAGGTTGAGA

AGTACAAGATCATGGTGCTGACAGATGTGGTTCCTGGTGAGAACCTTCTT

CTTGCTGTATCCTCGTATGTTGGGGAGAGAGGTGGTGCAGAGAGGTACTC

TAGTCTCTCTTCTTGCTCTTCTTAAAAGGACACTAATCCAATTATGGAGG

CCTCAACCTATGACCTCATCTAACCCTTATTATTTCTCAAAGGCCCCACC

TCCAAATATCCATTGGGGGTTAGCACTTCAACATACAAATTTTAGCCGGC

GGGGGGGATGCCAGCATTCAGTCCATAACAATCAACTATCCTCATTATTG

AGAGTTCCACTAGGCCTTCTCAAGGGTTGACAATGATAGTGCCCTGCTAG

CTATAAGAGTAAAAAAATTTTCACATCTAGAAAATGGGTGCACGAATATT

GGTAACTGTTCTAATGTCTATGCACAGAATTTTTAGAACTTGCTCGAGAA

ATTTGTCTTTCCTGACAGTGTTGCTTCTCTTAAATTCCAGTCGAGGGCCT

TGAAAAAAATAGGTTTTCCATCAAGATTCTCTGAATGAATAAATGAAATG

CTCTGATATTTTCTTTCAATATTAAGATAAAGCAAAATGTATACAGAATT

TTCTATTTTCAGTGTTTCCAATTACTGCATGGTTTGGCTTATTACCATCT

CTAAGTCTAACCCTGGGTCATAGGAGTAAAGCCATTGGGGGTCCCCTACA

AAGGATACAAGGCAGTGGTAGATACAATAAGCCTGACAATTGGAGTCAGA

TTTTTCTCTTTCATACAGGAACCATGGAACTGTTGCCCTGGGCCACAGCT

GGCTCTCAGAAAAACGTAGAACTGGTCGAGGAAAGGAAAGGCAGGGAGTC

CTTGATTCACTTGTTATCAGAAAATTGTCTGTTCAAACAACAGAATGTTT

GAAAAGATAAAATCAAGTCTCAAATGCACAGAATAAGAATGAGGGGAAAA

CACCTTTCTCTGTAATTAAACAGAAAAGTGTGCCATGAAGTGATAAAACC

CAGTCAACCTGAAAATGTGAACATCAAGTGTAATTGTGCAGAACGATAAG

GTAATATGAACAGCACAGGAATATATAGGCTCACGTTGGCATTTTAAAGC

AATGGCACTTTACCTGTTCCTCATGAAGGATTTGGTTTAGTCTAATCCCT

TGTAGGAGCCACTAAGAGGAGAAGGCACAGTCCTTTTGGGATGTAAAATG

GGGAATTCTTTCTATGATTACAAGAATTTGTAAATTGCTCAGAGTTTTAG

GACAGCAGGTTATGTGTCTCTCAATGTTGGGTACCATGCCAAACGTACTT

TAAGAGACATAGCAATAAATAGAATGGGATTCATTTTTTTCTTAATGTCT

GGCAGGGCAACCAAAATGCCCACGTTTCCCTTCAGTAGCTTGGTATTTTG

GTAACTAAAAACATGTTCCAGGGAACTCCAGAATATGAAACATTTCAGAC

AATTTGAAACTGTCAAAATTTTCACTTCTTTATGGGACAAATAAAATCTA

ACTTTATTCAGATTTTAAAGTATCTCATAAAAGAGTAATACTTTAGATTT

GTGCTGTGCTTTATACGAATTGGATGAGGAACTCTTATACATATATAAGT

GGATTTTATTTTCACAACAGTCCCTATGGTAGATATTGTCCTTATTAAGT

AAATGAGAGAACCAACTATCAAAGATATTAATATTTTGCATAAGATCACA

CAGATAGCGGAACCAGGATTTAATCCAACTCCTCTGATTCTAAAAATAGG

TGTTAGATGGGTATTCTTTCCCCAAACCTATGCTGAAAGGAGGCTACATT

TGGAGTCAGTTATTGAAAGTTTAAATATATGTGTATATATATATATATAT

GTGTGTGTGTGTGTGTGTGTGTGTGTGTGTATTCTTATATACTCATGCAT

GTAGATAGTTATAAAGTACATGCTTATATGTCAATTTTATATATACGACT

GATATATAAACTATCAATTTGTGATAGTTTATTTCAATTAAGTTCAAACA

TATTGGGTATTTATTGACTCATGGGACTTAAAATTTCTCAGAGGCATTAA

TTTCTATGCCTTTAAGCAAAATAATGTTTAGCCCGTTTTAGAAAGATAAA

AGGCTAACATATGTTTTGTTTTTGTTTGTTTTGTTTTGTTTTGTTTTGTT

TTGTTGAGATGGAGTTTTGCTCTTGTTGCCCAGGCTGGAGTGCAATGGTG

CGATCTTGGCTCACAGCAACCTCCGCCTCCTGGGTTCAAGCGATTCTCCT

GCCTCAGCCATCTGAGTAGCTGGGATTACAGACACGTGCCACCATGCCCG

GCTAATTTTGTATTTTTAGTAGAGACAGAGTTTCTCCATGTTGGTCAGGC

TGGTCTCGAGCTCCCAACCTCAGGTGATCCACCCGCCTCGGCCTCCCAAA

GTGCTGGGATTACAGGCGTGAGCCACGGCTCCTGGCCGCTAACACATGTT

TTTAAAAAATAAACACCATTCGAGTAGGAAAAGTGGGAGAACTGGTGGTT

CAGCTAGAGATACGGAGATGGGAGGGCAAGTTGCTTTCTCATGTTGCCAC

ATCTGTAAGTTACAAATGCCATAGATTAGGAATTTCAACGTTGTTGTGTC

TCTCTGAGGACATTCTCCATGTAGTATATGTTTCGCCTCCCTGATGGACA

GTAAGCATCAAACGGTTACTCCTTTATATTTGAATTATTTTAATTCTAAC

ATCTTGTTCTGAAAATAACGGGAATGCCATAAATACTTACTGATTGATTG

GACTGTCTCGCAGGGGAGCTGGCAGCTAGTGTAGGCTACAAGCTGCCATC

ATCATCTCCATGACCATGAGTCATCACTGGAATTACCCTAAAGAGGTTAA

TAAAACGCCTTGTTTGAAAAGCAAACAAAAAGCCAGAAGAAACCCAAAGC

AAACAGACCCAGACTTTGCAATGGGTAGTGAATTTTCAGTCTTAAGGGAT

TGAGAGTCCTTTTAGTACAATCTCTCATAAACATATAACAGATTTTAAAA

GCACCTTGGTCTGATTCCAGTTCACATACCTCATCCTTGCAGAATACAAC

ATACTAGGCCATTTGAAATGTTCCTCAGTTTACTTTTGGAGGATCTGCTG

ATTTGAGCAAAATGGAAATGAAATGTATATAGAAAGCTCTTAATTTTTTT

TTTTTTTTTTTTTTGAGACGGAGTTTCGCTCTGTCGCCCAGGCTGGAGTG

CAGTGGCGGGATCTCGGCTCACTGCAAGCTCCGCCTCCCGGGTTCACGCC

ATTCTCCTGCCTCAGCCTCCCGTGTAGCTGGGACTACAGGCGCGCGCCAC

CATGCCCGGCTAATTTTTGTATTTTTAGTAGAGACGGGGTTTCACCGTGT

TAGCCAGGATGGTCTCGATCTCCTGACCTCGTGATCCGCCCGTCTCGGCC

TCCCAAAGTGCTGGGATTACAGGCGTGAGCCACCGCGCCCGGCCAGCTCT

TAATCTTAAGAAGTTCAGTTTCGCAGCTCCCCCCCCACCAACCCCCAATC

TTGAAAGCAGGAATTTGGATTAGGGTTTTTTAATAAAACTTTCTTTTTCT

TATTACCATGATGTTTTTTGAGAGGGTACAGAGAACTCATTAAAATGTTA

TGTTATAACTTAAGACAAAATGGGAATGAGAATTTGCATTATATAAACAA

AGTGTATGACTAAGTAACCTGTTACATATAAAGTGTTTGACTAAGTAACC

TGTTGTCCCTAGGAGAAGTAGAATAAATATCAACATGTGGCAACTAACAG

AATGTGTTGTGATCACTAAAGCAGCACATTCATTGACTTTACACTTCATT

AAGTAGGTAACGAATTTTACAAATTTTAGGACTTAACCAGGCTGTCCATA

ATACTTTGCATCAGTAAGTACCAAAATCTACGATAGGGCACTTCGGAGTT

CCTTAATTTAATAATATTGATAATATTTTGTTTACATTTTGATTTAATTG

TATCATTTCATTATTTTTGTGTCCTGATTCTAAATATATACGTCAACAAG

CTATATGGCCATTCAAAACAGGTAAACTTTAAATATGTTTTTGACATAGA

AATTATGTAACAGTCTTCATCATGAGTATAGGAGTCAGATTTCCAATTGA

AAATTTCATCCTCTAAAATACTTGGCATATGTATACAGTTGTCTAGTTCA

GACAAAAAAGTATTGAATATAAAGTTGATTTTGTAAAACCAGAATTGGCA

TCCTTAAGGAATACAAATAAAGGGAATCTTAAGATAATATAATAATGCTG

ATTTGAAATGAAAATTAATAAAATTGTTATTCTATTTTAGTATGCTAAAT

TGGACAGATCCTTATTTCATTGAAAAATTAGATTCTTCAGTATATCAGTG

CACTGTGCTAATAACACAAGAAGATTAAATTATGTCCATATGTGATTTGG

TAGAATTTAATTATAAATATAATAAATCCTTCAATATGTACAGCAGTGAA

TATTCCAAATTTATCTCTCATATAAGTTGTGAGCAACTTAAGGATAGGCA

CCATCCCTTAACATACCTAGATTAAGAACAGTACCTAGAAAACAGTCATC

AAATGCTTCCTAAATAAATTTTCACCTTCAAGCTTATGGCAGCAAATTAA

ACATAGATTGACCCTTTTCTAATAGAGACAGAATAGATGAGACTGATGAC

TTTTTTTTATAAATGTGTAAGTCTGCAAATATTTAGTAAGGATCTAATTT

ACACAAAGAAATCAACCTAGAGTGAGATTATTTTATTCATTTCCCGGAGG

CTGTGACTGAATTATATTTGCAACTTGCCCTAATTTTTATACTTAATAAT

TTGCATTAGTATTTAAATTATGTGAAGGAAAAATATAAGTTTAAAATACT

GAAAAGTATATTCACACATTCATCAAATGTTGATTATGTGCTTACAGTGT

GCAAGGTATTGGGGCTAGGAATTATGTGGTGTTCCAAGGAAACCATGATA

CAGCCTCTTTCATTACAGTGTTTATTATTAGGTAGAAAAAGTAAAATTAG

TATAATATTAGATAGGATATATGTGTCATAGGAGTAATATAAACAATATT

TTATGAACAATGTATGAAGGTATAATTTTGTTCATTTATCCATTTATCGC

TCTGTACATCCATCCGTTTATTCATTGTTCATTACTTCATTTAATAATAC

TTTAGTGACCATTGGAAAAGGGATTTATAAAAGTGAGTAAAATGTTAATC

ATTTTCTAAAGAAGTTGATAATCTAGGGAAAGACAAATGTGTACCAACAA

TAACTTGGATATAAAGCATAAATTAGAGGTACAAGCAAAGAAAAATGAAA

GTACAAAGGAGAAAAATATTTGGCTGCATGAGAGCAGGGACTGTGTCTTG

TTTCATCATAGATTTTCCAGAACCTAGATCAGTGTTTGGCACAAAGTAGG

TTCAGATGAGTTTGTGTTGACTGACTGTCTACTGAAGTAATTGTGGAACA

TATCATAGAAGAAGTAGATCAGAGGCTGGATCTTAAAACTGGGAGAAATA

CATTTCAGCTCCGTGAAACCCCAGAAGTGATATTTGACATTAGAAAGCAC

AAAGTCACACTCTAACAGTGACAGTACTGAGTGAAGTAAGAATTTTCTGC

ACCCCTTCTTCTTCTTCCACTCCCATTAACCCTTGTTAGGGTGAGAAATA

GCAGCTGGTGAATGGTAGAGAAGACTAGCAAGACAGAAAGAGGAAGCCCA

CCATGCCCACTACCCCAGGTAGTAAAGGACCTGTCCACTTCCATCCTCTT

CTAATTGAGGAAGAAATCATATATGTGAGTTCAGATTGAAGTATTGATTA

ATATATTGAGCTGGATATTCTAATTTTAATGTCTCGATATGGAGCAGGAC

TTTGTTTCCTTAATGATGACCAGAAAAGACATGAAACTTACCCATATTTT

CACCCAAGAACAGAACAGAACAACCTACTTGAACAGATTTAAAGGGAGAA

CCAAAGGCAGATAAAGTTGTCTTTATTCTTACACAGCATAAGTCCTGATT

ATTCATCAATTTATACATTTTATGAATTTAAGAAAAAAGGAAAGGAAGAC

TATAAAGGGATTCATATCAGGATACACATGAAGGTAATGTAGTCAGTTTT

TTAGTGGAACCAAAATATTACAGTCATCACTGAACGAAAATATTACAATC

ATTACATCATTCACTAGATTAAGTGAATAAATTAAAAATATATTAAATAA

AATTAACAAAGACATGAGCTTTTCAAAGTGTGTGGTAACCTGGAGCAATA

AGCAGTGTAGTTGGAATGTTACCTCTTAAGGATTATCAAAGAGGCTGGCT

GTTCAGTAGGACAGTGGTATGGTAAAGCAAGCTTGCTACATTGCAGAACT

CCAGAGGGCAACATTCTAATTATCTTTAGCTATGGGGTCTGTTCCCATAG

GATAACTATAACTTAGTAGGTTGACAGAGCCTCCCAAGAAACCACAGCAG

TGCTCAATTGTGGCGAATTACTTCTCTATCCCATCAAAATGTCGTGATTG

GACCCACAACTGTGCATACATTTTTTTGGCATTTTCCTGCCAGAAGCATA

TTTACATTTCTTTAATATCCAGTCATATCCTTCCCTTATTTAAAAATAAT

TATTCAGAGAATTCCAGTTTTTTGTAATCCTCTCTTCTATGAATTTTAGC

AACACTTATATACAGATACTTGATATTCTGGAGAAATATGCTTCAAGACC

ATGTGGATTAACAGATTTTCAAATGCCGTTATAGTATATGTATATACTAT

ATATGTATAAGTATGTGTAGTCCCCAAAATGCGTACTTTAAAATCCTCAT

AATAGATCTCTTACAGATGAGGAAAAGAAGATGTAGAAAGGTTACATAAA

GTAGCTAACAAGTATTCCAAATAATATTGAACTTCAGTCTGACTATTCCA

AAATTGCATTCTTAACTCTGATTTCTATATTTGTTTTCCATTCTAAACTG

TGTATTGTGATATAAGTTCCTCACTAAGGCTCTTTTTCAGGGTCTTCCTA

ATACTAAAGTCACTCTTACAATGAGTATTTTCTTTACGTGTGAAATCCAA

TAGGCAAAAAAAAAAACTTGGCAATAAATTTTAGGCATTAACCTCATGCC

AAGTAATTATCAGAAGGCTGTAATGCTTTGAAACTTCACAAGTCTGATTT

TAAGATAATGGAATGAGGCTTGCATTGTGAACTTTCTTGATGCTTTACAT

TGCAATATGCTGTATAGATTGACTTCCTAAAAATAAAAAATAAAAAAAAA

GATTTGTAGAGCATACTGGGAAGGTCTTGCCAATTAAAAACCGGAGATTG

GCTGAAGGCTTGCAGCAATTGAATTTTGAATACAGATGGTGTCAAATCGA

GTGTTTCCATAGCAACAGACTCTTCCTTAATAACTTTTAGATGGGAGGGG

GTACATAAAAGAGAAAACCATCTTTTAGCAGATGTATGTTTTCAGCATGT

TTTCAGATTGATTTTGGAAGCTAATTTGTACTTAACTAGTGATTGTTTTA

AGTGGATCTAAATATTACTAAATTCTCCTGAGGAAACATTTTGAGAATAA

CAGAAATAAACTCTAGGAACTTTATAAAGACATGAAAAGGGCACAATTTT

ACAAAACCTTTTTTTTTTTGGTCTGGAGCCAATCAAACAGTATTTTATAT

TGAGTATGACCTATCAATAGTCAAAGAGTTCTTGATTCTTAATGCCTGTT

AATATTGAATGTTTAGAATATGGGTAAAATCAAGGAAAAAGTGCTATGTA

TCTCATGGATTTGGAATATCTTAATATACTCTTTCTTCAGGTTAATGATT

ATTTTTAAAAAATGATGATATAAATCATTTCAGGAAGGACTGCATAAAGA

GGTCATTGATAGAGTTCATATCATATAGCAGAGTTGTCGTAGATTTAATG

TCCAATCCTCAGTTTTGCATAGCCAACAACTAAATTTTAGTAGTTTACCT

AGAGATGATCAGGCTCTGGGAGACTGATTCATGCTACACAAACTCACAAG

GCACTGGTTTATAAACCATTTGAAAGAAGAAATAATAATTCAAACTATTA

TTTTACAACAAGACTGGCAGGAAAATCAATAATGGTAATGTGTTCGGGGT

GTCCTCCCCAGTGAGGACTATCCAGAAGACCAAATGATAGATTATTGTTC

AAAGTGATAGAATTGGGAGAAGGGTAATAAAGCATTATGAAAGGATGCTT

CCTTAAGTGAGAAAAGCTACATAAACTACCCATCTTTACTATTCTAGATG

ACCTTAAAAATATTAATAGAAACAGAGAAACAGACAATTCATCTCAGATG

GTACAAAAAACATAATAGCACATGTTTGCTCCCTGGCTTCCTTTTAGATT

CCCTGCAAGAGGTTTTCCCCCAACCACCACCATATACAGATATACAGCTT

TACCTTCACTCTCCTATTATCTGTCACAGAACAATATAAAACTCAGATAT

GTTAGTATAAGTTAATAGTTGGTAGCTTTATGTTAATGACATTGCTACCT

GTTACCCCCAGATTCATCTGGGTTACTTCATCCCCAAAACCCTTTCAAAA

CGACATTTCCCACAAAGATTTCTAGTATTACTGGCCATTCCCGGACATCT

TGCTTCCAACATAAGAAATTCTGACATAATAGGGATATAAGAAGGCCTTG

GGAATCTGTATTTATAACAAGCTCCCCAGGCTATCTGAGGCTCAGCCAGT

TTATTTTTGCAGATAGCTATTACTATCCACAATCTCTTTTATCCTATTGT

AATTGCAGAAGAAAATCTTTCTTTCTAGTTTCTTTGAAATTTCTGGAATG

TTAGGACTTAGAGCCTCTAGGAACTTGTACATAAAAAGAGAGACTTAAAG

AGATATCAAAGTAGAGATAGAGATAGATACAGATAAGTGCATAGACATAG

AAGTAGACATATATAATAGAAACATATATGTATACATAGAAATAGAAATA

TGCCATTTAGAACTCTTAAGAAATATGTGCACTCTTAAAATATAGTTTAA

AAATGTAAAGAACTTGCTATTAATTGAAAATAGCATAATAAACTTAAATG

AAATAAGGACCCCAAACGCAAATATTTCTCTCTGACACACCACACACACA

CACACACACACACACACACACAGACAGAGAGAGAGAGAGAGAGAGAGAGA

GCTGTACAATAACAACCAATTCCTGACCCAGATAAAGAAAAAATTTTTAT

TTATCTTCTTTCTCATTCTATTATTATTTTTTCTAGATTAAATTAAAATG

GTAATTATGTGTAAGAACAAGCATTATTGCCATATAAACAATGCAACAAA

GGAATCAATTGAAAATTAAGTGAAAATAAAAGGTACAGCTTCGTTAAAGA

CCCAGTTCTTAGAGCTTAGTCTCAAACTCTTTGTACTCTGCACTCTTTTC

TTGATTTGACTCGATTGATTGATTGATTGATTGATTGAGTCAGAGTCTCA

CTCTGTCACCCAGGCTAGAGTGTAGTGGCTCAATCTCGGCTCACTGCAAC

CTCCACCTCCTGTTTCGAGTGATTCTCCTGCCTCAGCCTCCTGAGTAGCT

GAGACTACAGACATGTACCACCATGCCCAGCTAATTTTTGTATTTTTAGT

AGAGATGGGACTTCACCATGTTGGTCAGGCTGGTCCTGAACTCCTGAATT

CAAGTGATCTGTCTGCCTTGTTCCCCCAAAGAGCTGGGATTACAGGTGTG

AGCCACCACGCCCAGCCGATTTTACTCTCTTTAGAACTGCAAAAGTAGGA

ATCTAGCTCATATGCAGACATTCTAGAAAGTTTGATTTCAAAAGTCTTCT

CAAAAGAAAGAGAGCAAGAGCAAGAAAGAAAGCAGAGAGAGAAAGCAGAA

GATAAAATGGCATTGTTTGAACAGGGATGGAAACTGAGTAAGAAATTTGG

TCACTAAACACTTTAGTGTCTATCATTTAAGATTGTAATTTGGTTATTTA

TCACTGGAAAGTGATTAATAATCTAAAATGCATTTTATAATACTAATACT

ATTAAAACATTAATTTTTGGAGAAAGTTTATTATAGATTGATTTATACTT

ACCACTGAATATTAAAATGTTTAATGGAAGTAGTTTCAAATAGTATTTAA

TGATATAGGGAATTATTACAACATTAATGCTCAGGAAAAAAAGTAGGATA

TGGAATTATTTATGCCATAGGATCCTAATTTTGTAAAAAACAGAGAAAAC

CAGCAAAAGAAATTATAACTGGAAGGAAATACATCAAAGTGGTTTGCAGT

TATCACTCATGAAATTGAGGTGAACTTGATTTTTTTCCTTTTATATCCAT

CCGTGTTTTAATACCACAGACATGCTTTAGATCTGCCCGTATATTGTGGC

TTTCAGAGGGTAATAAACCATTGTGACTCAGAAATAGCTAAGAATTTTTA

TCTCTCAAGAACAAATTTTCACTCCCTTGGGGTGTCATTATCTGTTGAGA

ATGCATGCAATAGTTCAAGAGCCAAAAGACTCTGATTCAGTAAGTTTAGG

GTAGAAAAAAATTAAGTACATTTTTAAAATAGAGCTCTGGTGTTTCAAAG

GCAATGTGCAAATATACCTACTTAATATTATTCTAATTTTTTCAGGATAG

CTGAAATATAAACATCTATTTTTAGTAATAACACAAATGATGGAATGCTT

TTACATATTTATAAATCATTAAGGATTTGCTTTTTGTTTCTACTGTCTGG

AACATATAAATTTGAACACAATTTAGAACAACATTCAGGAAATATGATTT

ATTTATTACCTTCTTGACCTTTATTTTATTTTTACCATCTACTTTTATGT

AAGTCTTTTTTTTCAATCATTGTTTATTTCTTTACTTTTCCTTCCACATA

GAATTAAAGGGAGATTCGGGCTTAGTGTCCTTAATTCATAGTTCCATTGT

GGCTATTAAAAGGTGAACTGAAAGCTTGCAAACACGTGGTACCTTGTAGA

TAATTTTCTCCAGTCAGACAGTTAGATAAAGGCCTCTGAGGTTTCTGGGG

TCATTGTTGAAGCTATGTTTTAAAATCCTATATCCTTCTCTCATTGTTGG

TTCCTTTTCACAACACAGAAGTTTTCTTTTTTTTAATTTCAACTTTTAGA

TACAGAAGGTGCATGTGCAGATTTGTCACGTGGGAGTATTGCATGATGCT

GAGGTTTGGAGTACGGATCCCATCGCCATGTTAGTGAGCATAGTAACTGA

TAGGTCGTTTTTTTAACCCACTCCCCTCCCTCCTCCCTCTAGTAGTCCCC

AGGGTCTATTGTTCCCGTATTTATGTCCATGTGTGCTCAGTGCTTAGCTC

CCACTTATAAGTAAGTGAGAACATGTGATATTTGGTAGAACTTTTCATTT

TTAAGTTAAAAAACAAAACAAAATGAGGATGAGTGGAAGAACTATTCAGC

ACAGCAGGTTATAAACCAATTAGGATGATGACGCCCTGAATGGAGATTTT

CATGAACATCTCATATTAGCTATTTCAGCTTTGGTTTTTTTAATGTTCAA

AGTAAATAGAATAATGAAGAGGCTATTTAGGAAGGTTTAGACTGAGGGAA

AAAAATCCTTTCATTAGGTTCCAAATAACGGTTAGCTTATTAAACAGCAA

GAGGCAGAGATTTAGCAGAGAAAAAATAAAAAGATTTAAAAAAAAACAAC

GAGATTAAAAGGTCAGTAATACCATTGGAACTGGCAGCATGGCAAGTTTA

TATCAGCTATCTTTTGTTTTGGAACACAACTATGCTAATTCTGTTCTGAA

CCTCTTGCTAATGCCTGTCTCAAGAAAATTTAACATACTTTATCTGTGTG

TACAAAAATACCTAAGGACAAAGCTATTACCCAAACTGTATTCAGATTGA

AAGAATCCATATAGAAATTTGCAGCTAACGTATTAGTCAGTGTATGTAAT

TTCTACTGCTTCACAGCACAACTCTTTCTAATTTTCAGGAGCAATATAGC

AACTGCTTGCCAGCCAAGAGAAAACCATAGGAGCATTCTTATCATTGGAG

CCAACATTAGTTCTGCCTACAGTGACTAACATAGATGCGTTTATTGCTAG

CTGGAATTTTCCATTGGCACTAGTTACATGTAATAAGTTAGTGCTTTCAA

ATGGACCGTGGAATATAGGAAAACTAGAGTCTGACGTAACCAAAAAAAAA

TGTTGATAAACTCAGAGATTATGAAAGAGAGGAGAGGAGTGGTTTTTGTG

GAAATGATAGAAAAGCAAAAAAAGATGGCAGGATTTGGAAAAAAGAAAAT

CAGGTTAGAGATTTAATTAGTAAAGGAGCTCCTTTTAATAATTATATAAG

AGTATGAGTTTAGAGTAACTGCCTGGCTAATATGTACAATCTTCAAGTTC

AGTCGTTTTCCAAAATTCTAACTTTTAGCATTTTTTTTTTGTAAATTTTA

AGTGGAAATCTTCCACTTTTGTTGACTAACTTGGCTACCTGATATTTTAC

TCAACCTCCTACTTTCTTGTTCTCTACTTCTTCTGAGTCTTTGCTTCCAC

AATGAGGTAGCTCATATTCCTTAAGTTTCCTGTCTGGTTTTGCTTTTTTT

TTTTTTTTTAACAGCTAAAATAAACTCTACAAGCATTTCTATCATTTTCT

TTACATCCAATCTCTTGTCAGCCATGTCCTAATCACTTTGGTAAAGTATC

AGTGACCACCCAAGAGGTTCACTTTGCTTCGTCAAAGGACCACCTGTCAT

GCTTTATCTCCAGAGCTTCTGCCTTGAAAGTAAGAATAATATAACCTTTC

CTGAATGTCTTATTTCAATTTCATGCATTTGATCTGCCTCTTCAGACCAT

TCCCAAGGCTTTGCCCTTGTTCTTTACCCTCATGGCTTCAGTTATTCATC

ACTATATGAATGACAGCTCTCAAACTGACAGCTCTGGCCTCTCACCTATT

TCATCTATTCCATATGGCAAACTGTCTATATTACATTTCATCATGAGGGA

TAATTATAATTCCACATTCAACAAGAAATTGTGGGTCCGTGTTGATTTCC

AACAGCAACCTTTATTTTTTAGACTATGATAGAGAAAGGGTCAACTTTCT

CTCTTACTTATTTATCTCAAATAACTCAAAGTCAAGCTAGAAAAGCCAAG

CAAGTAATATTTCCCAACAAAGCAGCTTTAAGCAAAATGATTTTCAGACA

CGACATGCAGCCTGTATTGTGGAAGAAGCACAGTGACCTAGGTGTACTTT

GCTGAAAAGCAGTGGTCACAGTCCATTAGTTTCATCTTTTTCCTGCAAGA

GAGAAAAGTATGGCAGGGCCAAGACTCCTAAGACCCCTTAAAAAATTGTG

AGGTTTTTATAACCCTGTTCATTTCCCATCAAAAGCATTCATGAAGCACC

TGCCATGTACCAGATGATGCATTCTAGGCAGAGGGAAGAACTTGGGCAAA

GTCCTGTACATTTTATCTTCTAAATCTTACTAATCTTTTCTTATCTTTCC

ATCTACCACCCACCATCAGGCTGTGCCTGGACTCCTATGCTAACATCTTA

CTCTGTTTTTCTGCATCTTTTTTGCCCTCCCCTCCAATCTACTCTCCAAT

TAGCAACATGACTAATGTTTTCAAGCCCAGATTAGACGGTGTCACTTCCC

TGCTTTAAACCTTTCAGTGGATTCCCATTGCACTGAGGTTGAAGACCAAA

ATCTTTACCATAACTCACAAGGCCACTGGGAGCTATGAAAATTTTCTTTT

TTAGTTTTTCCTTTTAATTAGTGATGCATTCAATAAGTGGAGTCTATCGT

GAAAGCATAGATTCCAAAGGCATGTGAATACTCAATTATGTAATTTACTT

ACAGAATGTTTATTACTTCTTTTTTAGCTTTGATTTTGTATACATCCTAA

CTCAATCTCAGTTATATAAGAGATTATAAAAAAGTTTGTGATGAATGCAT

ACATAAAGGAACAAATAATTAACAAATATATAAAGACAAATCAGTATTGA

ATGACCAAATGGCTCTTTATTTATTAAAGAACTTAGAAAAATATAGTCTT

GCTTGAGACCAAGTTCTGGCAACGTATGTGTGTCTTTTTGTTTGGGTTAA

AAAGCATTAGTTTTATATGTTTTAAAAGGATAGGATCTTCCCTTGGCTGC

AAATAGTATAGGCAAAATGCTATTCTGTATCTTATTTTAAAGAAAATAAC

AATCATACCTTATTATTTGCTGACAGTTTTGCAGTTTACAAAGCACTGTT

CACATGGATTACCTTATTTTAAATTCCTCAATTTTTTTTCCTTTTACTAT

GGGAATTCTGTTTCAGGAGAATTTCCCTTTAAAATTAAGGTGATTATACT

ATTCTGTTCCTGTATTTCGTAGCCATATGTTTCACCAAGGTATTGTCTCT

CTCCAGGTCTTTCTTCTCTGGATGTATAGTTATGATTTTCCAGGAGGTAA

ATGAAAACAGTGGGAGCAATTGACAGAGTGTTTTTTGTTTCCCATAAACC

CAAGCATTGCATTTAAGGAGCTGCTGTACTATTGTAAGAGTTCCTATACT

AATTTTAAAAGTTCATTTACATATTTTGTAGATTTTTAGGATAAGTCATC

AGAAAAAAATTAAAAATAAATTTACATATTTTGTATTAAGCTGTTTTGTT

ACTTGAATGGGGATTATTGGAACGAGGAAAGAATTACTTTTATCTCCCCA

TTTTTCAAATCATTTACTATAACATTCATGAATTGCTGAAGTTTAAACAA

TCAAAAATATCTGAAAATGGAGCCTAGGAAGGGTAACTTATGTCTTTCAT

ACTCCTTCCTTTTTGGTTTTTCTACCAGCATTTTAGTCTAAAATATATTT

TATTTTCACTGATCCTGTGTTTGTCTTAATAATATAGTATGTATGTTAAA

TGAACCAGAATTCATTGATCTTTTTTTTTATTATTCCTCTTTTTGCTTTT

CTGGAGAAGCATTTGAAGAAATCAGTTCAGGCTACACTTCATCAATTCAT

TTCCTCATCTTCCCCTTAATACAAAAGCCCCATTTTCACTCTTCAAAACA

TTTGATCACATACTTAATTTTCATAGCATTGACTAAATGTATGTGTTTAT

TTTGTTTATATATTGCATATTTGTGATGATTTTCACTTTTACAGTTTAGG

CATGGGTAGGAAGGACACTTACATGAGTACTTAAGTTCTTGGTTATGACT

TGAGAAACTCACAAGGTAGCATGGAATGGCACGTAAACAAGGCTTTGATA

GGGTGAAAGGGAATTATCAGGACAAAACTGACTAGTGGCAGTGACTTTTG

AGCAGAGTGCTGCAAACTGAAAAGGCAGAGAGCATTGTAGGCAGAGAGAA

AGCATAGACACATGTGAGATACATGTGAAATACTTAGACAAATGTGAGGT

GTGTGGTAACATTGTACATACCTAGGGCTCCTTCCTGTGTAGCTGTAAAG

CTGGAGTATAGGCATCACAACAGGCAGCAGAAGCCAAGAAACTAGAAAAG

TAGACAAAGGCCACGTTATAAAAGATCTTGTAGGATAGGTAAAGAGTTTT

AATACTATCCTGAAAGCAGTGTGGGGTGGGAGGCATGACATTAAGGAAAT

TTAATAAGTGATGAAAGACCAAGATTTTCATCTTCAGAAGACTTCTCTGG

AGGTGGGTGGACCACTTGATGTCAGGAGTTCAAGACCAGCCTGGTCAACT

TGATGAAACCCCATCTCTACTAAAAATACAAAAAAATAGCTGGGCATGGT

GGTGCATGCCTGTAATCCCAGCTACTCAGGAGGCTGAGGCAGGAGAATAG

CTTGAACCCAAGAGGCAGAGGTGGCAGTGAGCCAAGATGGTGCCACTGCA

CTTCAGCCTGGATGACAGAGGGAGACTCCCTATTAAAAAAAAAAAAAAAA

AAGACTTCTCTGACTAGAGTTTTAAAGAAGGATGGGGAGTGCAAAAGGCT

AACATTAAATCAGTGGTTCTCATCCCTGGCTGCACGTTAGAATCACTAGG

GGGAGTTTAAAAAAAATGCCAATACTTGGACTCCACCCCAAACCAGTTAA

ATCAGAGCCTTAATAGGGCCCAGACATTCGTAGTTTTTAAAGCAGCCCTC

TTGATTCAAATGCACAGACAAGGTTGTGTTCCGCTGAACTAGAGAGACCA

TCAGGAGGCTTTTAACTAAAGCAATCTAGGGAAGAGAGAGATGAATGATA

TAGAGGCCAGAAGAGCTATTAAGGAGAAGTGACTCGGCATCTTCTTGGTT

AAAGTATAAGGAAGAAAGAAGAATGAAGGATAATTTGCATGTTTCTCCTT

CAGTGACTAAGTAGAGGAACAGATGTGTATCAGTGATTAGGGGAGAGGAA

GAATGAAGATGATGTAATCCAGTTTGGATATGTTGGCTATTTCATAGAGA

TTTAGTAGGCGTTTGGCTCTATGGATACAGAGTTCAAGAGACAATTCTTA

GCTTAAAACACAGATTTTACTCTGATTATTTTAGAGATAGTAAATGAAGC

AATATACATACATGAACTCACCAGAGAGAATATGTAAAGTGAAAACAAAA

AGATCAAAGGGAGACCCCTAGGGAATAGCAACATTTAAGGAATGGGCCTA

AGAGAGTGAGGAGTGGCCAAATAGGCAGAATAAAATCCAAGAAGGAATCT

TCCATATTAATCAAAAGAGAAGGTTTCAAAGAGGGTTCGTCAATTGGCAG

GTACTGAAGGGATATCTGAAAACATAAAGACTTTAGACTCTCTAATACTG

TAGCAACTTTAAGGTCACTGCTATCACCTACAAAAGTAATTTCAGTGGCA

AAAGCCAAAACGTAATGGTTTAGAAGGGTATGTGAGGTGTGGATGTGGAA

ATAGTGGGTATAGACTATTGCTTCTCAAAATGTAATCACCTGAAAAATCT

TATTTTAAAATGCAGATTTTGATTCATTAGCTCTAGAGTGGAGCCTGAGA

TTCTGCATTTCTAACAAGTTATCAGGTGATGCTGATGCTGCTCATACACA

AACCATCCTTTAAGTAGCACTGGTGTAAGCCACTCTCCCACAAAGGAGGA

AGACATAGGGTTTCCATCAGGGGACGCAGGTGGGGTGTAGGACCAAAGGA

AATAATACTCTTTTGTCTTTTGTTTGGTTGGTTTTGGTTGTTTTCTTCTT

AAAATAAACATGGTTGTAGACGGAGATTGAAGACATAGGAGGGGTAAAAG

ATGGAATCACTTCTCATAGAAGATGGAAGAGACTGGAACATTGAGTACAG

CGAGGGAATTAGGCATGGGTAGGAGGGACACCTAAATCTGAAGAGAAGGA

GGTAAGGAAGAATTGAAATACAGAAAAGTTCTGTCAGTAAGCAATGTATG

GAATTGTGCTTTTTAGCCATAGTTTCTGTTCAAGAAATTGTTTTCCATTT

ATTTTTATTTCTTAACTTGAATAGTTGGATGGTGGAGTATGCTTGTAAAA

ATAAGAGTGAGGCCACTTTGTTTTTCGGGGTTAATTTCCGTTTTCTGCAC

TCATCAAGTAAAAGTTGAAGTGGCAAGGTGTAGATATGAAATTCAGTGTG

TGCTAAGGGAGAAAAAAATGCTTTTTATTCTACATGATTTTAAAAATATT

TATATTCCAACAAATGCATCAAATTTGATGTGCAAATTTACAGTGATGAA

TGAGTTTTATTGTGTGCATTGCATGCTGGTGACATGGTAATAAATCTGTG

GTGCTAGAATTATAATGGTCCCCTTTAGCTTCGCTTTAATGAACTCTTGC

TGAACACTTTTGAGTTGTTAGTACTTTATTTGCTACATTTGGCACTTAAT

TAGTTAATGACTGAGATGCTGGACCGATGGATCATCTAATGATATGTTAG

GCCTATTATCACATCTAGATAGTTTCTTTTCTGTGACTTGTAAGTGACCT

AAGATGATAAACTGAAATATTTTTGCATAGATATACATCAAGCTTTTCTC

CTAACTTCAGGCTTTCATCTTAAGCAATAGTTTCCAACACCCCCAAAAGA

GAAGTCATTATGTTTTTAAAAAAATTATTCATTTTAATGTGATCAAATAA

TATCACATTTCAGCATTCACCTATTTAATTAATAAAACAACTTACATGTT

TCATTATGACTGGATGTTGATATTTTTTCATAATCTATTATCCTCCAACC

AGTGGTAAAAACCCAATCCTCCTCTCACCCAGCTCATCTTTCCGTATGGG

AAGCAATACATACTTCCCTATGTTTTATTACCAAAACAGGAGAATGAGCT

TTCTTTAGAAGGTTAACTCATTTTCTCTATTAGAATATTCAGCATACTTT

TAAGGAGGTAATCTGGTCTTTGACAGTCTGTTGATTAGAAAATTAAGAGA

CCTGCCTAAATTCCATTTCCAACTCCTCTCCATACACATTGTGACTTTGA

GCAAAACGTTTTGCCATTTCCACTCGTATAAGTTCTGTTTAGTATCTTTA

AACTTCCATATCCCACAGATGGTATTTTTTTCTTCATTGGAAAGTGGTGT

TAGTGATTCAGAAAACTGCTTAAATAATACACTGCTTTGTGTTTTCTGTG

AGAGAATTTTTTTTTTTTTTTGAGACAGAGTCTCACTATGTTGCCTAGGC

TGGAGTGCAGTGGCGCAATCTCGGCTCACTGCAACCTCTGCCGCCCGGGT

TCAAGCGATTCTCCTGCCTCAGCCTCCCGAGTAGCTGGGATTACAGGTGC

CTGCCACTGCGCCTGGCTAATTTTTGTATTTTTAGTACAGACAGGGTTTC

ACCATCTTGGCCAGGTTGATCTTAAGCTCCTGATCTCATGATCCGTTCAC

CTCGGCCTCCCAAAGTGCTGGGATTACAGGTGTGAGCCACCGTGCCCAGC

CGAGAGAACATTTTATCTAACATTCTATTTTAAAATTTTTCAAATACACA

GAAAGCTGAAAGAATTGTACAGTGAGTGCTCATGAACCCACTCTCTAGAT

TTTGTTGTATCACTTTACTTCTTTCTCTACCCGCCAACCCCTATTATTTT

CTGATGCCCTTCAAAGTAAGTTGGTGACATCGGTTCCCTTTACCCCTAAG

TTCTTCAACATGCATGTCATTAACCAGAGCTCAATATTTGTTCACATTTC

TTTTGTTTGTTTGTTTGTGGCAAAATTTGTATAGACTAGAATGTGTAAAT

CTCATGTGTATTATGAAATGAGTTTTGATGAATGGGTATACCTGTGTGAC

ACATGCCTCTCTTAAGATACAAAAAAATAGCCTTGGCCCAGACAGGAGGG

AACATTTTTTAGGTTGGCTTGAGTTTCCTTTAACTGACATGTAGCATGAC

TGAATATATGACCATAAGATTGCCAAGTTGAAATTTACCAAAGGTCCATC

CAGGGGAACAGTATGGCTATTGATACGTCATTTGTTCATCTAGGCACTGG

GCTGGGTGAGTTCTTCACAAAAACCTTGTAAGGTGAGCTTTCAGCTCTGA

CAGAGGCATCAAGAAACGTATTTCACAAACTGTTAGAGCTGAAAACATCT

CCAGGGATCATCCAATCTATCCTCCTTTGCTTTATGACTCAGGATTTAAG

AAGCCCCAGAAAAGTCCAACAGTGTTACCAAAGTACACGATTGCCTATTT

ACGCCCCTATAATTGAGAGCTGTCTAGGGGTCAGGTACTTTTCTCAGTGC

CTTGTGTACATTATTTCCTTTTATCTTTCTAGATTATTAATTTTCTTGTT

ATTATCCCCATATAATAGAGGAGTAAACAGTGTGAAAATAGCACTAAACT

GTTGATCTTTTCCATGTTCCAAACTAACCTTAAATGTTAGGCTAATATTA

TAAAATTTTAAAATTTAGTCATTTGTTCTGGGTCTGGCTTCCATGAACTT

AACCAAAATGCTTGGCTGTTTCTGCATTCTAGCTTTTTGGGATTTAGAGA

AAAAGCAACTTGGTCATCATAGTGGCCAGGATTTATTTATGTGCCAATAA

CTCCCCTCTGTTTTTAGCTTTTTGATCTGTTACATCTCAGATACTTTATT

CTCTATTTGTAAAACAACCACATTCCTCCTTTTCTATATCTAAGTGAAAA

AAAATGCCCGTTCATACTAGCTACATGGTTAGCATATATGTTTCTTCCTC

TTTGTGGGTTTGAGTTGAAACTTCTTTTGCAAAGCTTAACTATAGATAGG

TCTTCCACAGAACCCTGTTTAAATGCCTTGTGGAAGTAGAAGGGACATAT

CTTAATATTCCTTATAATCTGTGTTATTATTTGGACTTTCCTCAGTTCCT

TATATGTCTTTGTATGTCAAACATTGAGTTATGCTTACATTACTCTAGGT

ATAGTTGTGTCCTAGCTTTGTACAAGGTTACAATAATGGTGGTTTCCTTG

TTTTCAGCTTCATTCTTGATCATGCCTAACATTTTTTGATGGTTTTAGCC

TCGGAGAACAGTCTACTGCTATTTCTCCCTTTTTCCTGATCCCAACAAAT

AGTTTAGAAATCTTTGTCTTAACTGCAATTGGGATCATTTTTCCTTTAGA

GTTGCCCACAGTTAGTCATGTCCGTCACTCTTCACTCACTCAAATGACCT

CAAAAGATTTACCTGCAATTTAAGCCTTTCACCTTGGCATTTTCTACCTG

GAACTCTGGCGTACTCTGTGGGATCAGTGAGATCCATAGTGTGCACTCCC

TAGCAGATCGTGAATGTTTGTTGATAACTATAATTCATTGTTTAGTCTTG

TCTATACAGAGAAGCAGTAGCACCTTGCTTTAGTAGGATCCTTTATGCTT

TTATTCATTATTGAATTTTATCTTCACAATAATTCTGTGGTTCTGCAGTT

GTCATTATTTCAGTTTGGGAGTTGAGGAAACTGAGATCGTGGCAAATCAA

CTTTTCCCATTTAAATCCACTATTTCATCGCTAATCCAGGGTTATAACCC

AAGACTAACTGCTAGTCAAAGTTGCTTTCTAAAAATCAGTTACACAGACT

TCTAATTCTCGTGCTGTACAGTCAGTGCAGAAATTGCAACAGAACTGTTC

TTTGTTCTGTAGCAATGTGTATTCAAAAGGTAATCATTTCTTGTGTATAA

ATAACATTCCTCCCTAAGAGAGATGTTGTGGGAATTTATATCAGTCAAGA

ATGTATATCGATAATTAAACATAGCTAGTCTAAAAAACTTGAAGTGAAAG

ATTGGTTCGTATTCAATCAACTGAACTGTTTCTTTCTCTGCAATATTGTG

GTTCTTGACATGATTGCTGAGTTTCCAATTTGACACTTCTGGAGGTTCGT

AAATCAGGACACATGCTGGGTCTCAGTCTGTCATCCTGAGTCTTAGCATT

GGTTATTTATTTCCCTTTATTACCCAACATCTATTTACTGCACAAATTAG

TCGAGAAGCTTTCCATGACGGACCTTTGTGAGAAAAAAAAAATGTGTACA

TTGGGTAGCTCTTCAATTACAAACATGCACAGATTGTCTGGGTTTATGTG

CATATACTTTATTATTATTTCACTTTTGTATGTCAGATGAATTTGGTGTA

AATGTAAATCTAACAATATGGGCTCTAGTGGCCAAATACATTGCCATAAT

ATGTTTTTGATCTGTCAGTCTCCTGGGTGAAGTGTCGCAAGTGTGGTAAA

ATTCTGTAGTATAAATTGATGCTCAGTTATGGACAATTACCAGGTCTATA

TGATGTCAGACTACCACACTGATCCACTTTTAGGATAACAGCTTCGCTCT

GATGATCTTCAGATTTTAGATTTGCTTTCATTCCTTGACAAAATGAAATA

CATTTAAAACTTCTAAAATTGTCTTTTGTATATATTTCTGCAGGGTCTAC

TCTTGGGACTACTATAACAATCAATTGTACTGCTTTGTTTCTTTTATTAA

ATAGCAGAATAGCTGCTTGATTCGTCTGTATGTGCTGATGTGTGTCTGTG

AATCTAGTCCAGTTCACTGTCCAATAAGAATTTCTGAAATGTTCTTTGTC

ACATCAATGTACAGTCAAAATCGTATTGTGTTTGTAGCAGAATGATTGCA

TCTTTTATTATTCACAGCAGCCAAAATGACACATATTTCACACGTGACAC

CTTTTTTAAAAAAGATGAATTGTCCAAAGTTGTATGTAGAATATATTTCA

CAAATCAAATTCCCTATTTAATAAATGGTGCTGGGAGAACTGGCTAGCCA

TATGCAGAAGAGTGAATTTCTATAAATAGAATGCAACTGTGTAAATAGCA

TCCAAATCAAGAAACATAATATAATATAGCCAGAAGCTCAGGATCCCCCT

TCATGTCTTCTCCAGTCATGAACATTTCACAAGGGTGACTACTATCCTTA

CTTCAAAAGTTTTCTATTACTTTTTCCTGTTTTTGTGTATTATATGAAGG

AATCGAACAGTCATATCTACTTGTCTGTGGCTGTATTTTTCCCCCCAACA

ATATGTTTGTGAGATTCATCCATATTATTGTATGTAGTTGTGGATTGTTC

ACTTTTCTTACTGAATAATATTCTATTGATACCACAGTGTATTTACACAT

GTTGATATAGATGAGAAATTGAGTAGTTTCCAACATGAGGAAATTGCTGT

CAACAATTCTGCAATCCATAGCCATGCTATCAACAGTCTAGTACATGGTT

TTTGGTGAATATATTAATAACTATGCATTTCTGTTGGATATACACCTGGG

AGTGGAATTACAACTCAAATAATATTCATTTTAAAAATTTTATTTCGTTT

TTAGTCGACAACTATATTATGGGGTACATTGTGATGTTTCAATCCATATA

TATACATTGTGGAATAATCAAATCAGGCTAATAGCATATCTATCACCTCA

AATACTTCTCATTTTTGTGGTGAGAATATTTAAAATCCTCCTTTTTAGCT

ATTTGGAAATATACAATATGACAATATTAGCTATAGTTCCTGTGCTGTGC

AAAAGAACACCAGAACTTATTCCTCCTGTCTAACTGGAACTTTGTACCCA

TTGATAAACGTCTCTCATTTTTCCATCCACCCACCACTGCAGCTTTTGAT

CACCACCATAATACTCTCCATTTCTATGAGTTCAACTTTTTTTAAACTGC

ACATACAAATGAGATTATATGATATGTGTCTCTCTGTGCCAAGTTTATTT

CACTTAACCTAATGTCCTCCAGGCTCATCCTTATTATTCCAAATGACAGA

ATTTCCTAGGTTTTTAAAATTTTTTTTTTATTTTTAATTTTTTGGGGTAC

ATAGTAGGTATATATATTTACGGGGTACATGAGGTGTTTTGATATAGGCA

TGTAATGTGAAACAAGCACATCATGGAGAATGAGGCATCCATCCTGTCAA

GCATTTATCCTTTGTGCTACAAACAATGTAATTGTACTTTTAGTTATTTT

TTAATGTACAATTAAATTATTATTGACTATAGTCCCCCTGTTGCACTATC

AAATACTAGGTCTTACTCATTCTTTCTAACTATTTTTTGTAGCCGCTAAC

AATCCCCACCTATCCCCTACCTCCACACTACCCTTTGTAGCCTCTGGTAA

CCATCCTTTTATTCTGTCTCCATGAGTTCAATAGTTTTAATTTTTAGATC

CCACAAATAAGTGAGAACATGCAGTGATTTTCGTTCTGTGACTGGCTTAT

TTCATTTAATGTAATGACCTCCAGTTCCATCCAAGATGTTGCAAATGACA

GGATATAATTCTTTTTTATTGCTAAATAGTACCGCATCATTTATATGAGC

CACATTTTCTGTATCCATTCGCATGTTGATGGACAGTTAGCTTGCTTCCA

AATCTTGCCTGTTGTGAACAGTGCTACAACAATGTGAGAGTGCAGATAGC

TCTTCAATACACTCCCTCCTTTTCTTTTGAGTATGTACCCAGAAGTGGGA

TTTCTGGAACATATGGTCATTCTCTTTTTATTATTTTGAGAAACATTCAT

ACTGTCTTTATGGAGGCCGTTACTAATTCACAATACTACCAATAGTGGAT

AAGGTTTCCTTATTCTCTGTATCCTCATGAACACTTGTTATCTTTCAACT

TTTTGATAATAGCCAATCCAAAAGATATGAGGTGATATCTCATTGTGATT

TTAATTTGCATTTTTTGATGATTAGAGATGTTGAGTATTATACATATATG

TGTGTGTATATATATATATATATATATATATATATATATGCTGTTTGTCA

TCTTTTGAGAATGTCTATTCATATATTTGCCCATTTTTTTATTAGGGTTA

TTTGTTTTCTTGTTATTGAGTAGCTTGAGTTCCTTGTATATTTTGGATAT

TAGCACCTTATCTAATGTATGATTTGCAAATATCTTCTCCCAATCTGTGG

GTTGTCTCTTTATTCCATTAATTGTTTCCTTTGCTGTGCAAAGCTTTTTA

GTTTGATGCAATCTTACTTACCTATTTTTGTGTTGATTGTGTTTGGGGGT

CATATGCAAGAAACCACTGCCCAGACCAATGTCATGGAGCTCTTCTCTTA

TGTTTTTGTAGTTTTTAGTTTCAGGTATTACATTTAAGGCTTTAATCCAT

TTTGAGTTGATTCTTGTATAAGGGGTGAGATAAGGGTCCAGTTTTATTCT

GTATGTGAACATTCAGTTTTCCCAATACCATTTATTGAAGAGACTGTCCT

CTCCCTATTGTGTGTTCTTGCTACCTTTGTCAAAAATCAATTGATCAAAG

GTGTGTAGGTTTATTTTAGTCCTCTTTGTCTTATTCCATTGGTCTGTTTT

TATGTACTTGCCATGCTGTTTTGATTATTATAGCTTTGTAATACATTTTG

AAATCCAGTAGTGACATACTTCCAATTTTATTCTTTTTAGTAAAGACAGC

TTTGGCTATCCAGGGTCTTTTGTGGTTCCATGCAAATTTTAGGATTTTTT

AAAAAAAATTCTATAAAGAACAATATGCAGATTTTGTTAGTATTGTGTCG

AATCTTTAGATTGCTTTATGTTTAACAATATTAATTTTTCCAATTTATGA

ACACAGAAATCTTTCCATTTATTTGTGTCATCTTCAATTTCTTTCGTCAG

TGTCTTATAGTTTCAACACGCAGATCTTTCACTTTCTTGGTTAAATTCAC

TCCAAATATTTTTTCATGCTATTATAAGTGAGATTGTTTCCTTAATTTCT

ATTTTAGACAGTTTGTTGTTATTGTACAAACAATAACAATTGTTATCGCT

ACTGATTTTTGTAAGTTGATTTTGTACCCTGCAACTTTACTAAACTTGTG

TATGAATTCTAACAGTTTTCAGTGGAGTCCTTAGGATTTGCTGTATAAGA

TTATGTCATCAGCAAGAAGGGGCAATTTTACTTCATCCTTTTCAGTTTGG

TTGCCTTTTATTTCTTTCTCCTGCCTAATTGCTCTGGCTAGGACTCCCAG

TACTAAGTTAAACAAGGGTGGGGAGAGTGGGCATCTTTGTCTTGCTCCTG

ATCTTAGAGAAAAGCCTTCTACGTTTTACTGTTGTGCATGATGTTAGCTG

TGGGCTTGTAATTTATGGCTTTTATTCTTTTGGAGAACATTTCTTCTATA

CCTAATTTGCTAAGAGTTTTTCTCATAAAAGGATGTTGAATTTTGTCAAA

TGCTCTTTCTGAGTTTATTAAAATGATCATACGGTTTTTGTACTTCATTC

TGTTATATGTTGAATCACATTTATTAATTTGCATATATTGAAACAACCTT

CTATCCCAGGGATAAATCCCTCTTGGTCATGGTGAATAATCCTTCTAATA

AACTATTAAATATGGTTCACTAGTATTTCATTGAGAATTTTTGCATCTAA

TTTCATTCGTGATATTGGCCTATAGTTTTCTTTCCTTGTAGTGTCTTTGC

CTGGCTTTGGGATCAGGGTATTGCTGGCCTTGTAAAATAAATTTGGACGA

ATCCCTTCCTCTTTAGTTTTCCAAAAGAGTTTGAGAAAGATTTGTGTTAG

GTCTTCTTTAAATGTTTGTAGAATTCTCCCATGAAGCCATCTGGTCTTGA

GCTTTCCTTTGATGTGAGAACTTTTAAATACTGATGCAATCTCCTTAACT

CTTTCCTTAGCTGTTACTGGTCTTTTCAGATTTCCAATTTTCATTATTCA

GTTTTGGTAGATTATGTATTTCTAAGAATTCATCCATTTCTGTTAGGATG

TCCAATTTCTTGGTATATAATTGTTCATCGTAGTCTCTTAGGATCCTTTG

TATTTCTGTGTTATCAGTCATAATGTCTTCTCTTTGATTTCTGATTTGAT

TTATTTGAGCCTGCTCTCTTCATTCTTAGTCTAGCTAAGGATTTGTCAAT

TGTGTTTAGCTTTTCAAAAAACCAACTTTTAGTTTTATTGACTTTTTTTC

TATTGTTTCTCTAGTCTCTATTTCATTTATTTCTGCTCTGATCTTTGTTA

TTTTCTTCTTTCTGCTAACTTTGGGCTTAATTCATTCTTCTTTTTGTAGT

TTCCTGAGGTATAATGTTAGGTATTTCATTTGAGATATTTCTTCTTTTTT

GATGTAGGAATTTATTGATATAAACTTCCCTCTTAGCACTGCTTTTGCTA

CCCCCAGAAGTTTTTCTATGTTGTGTTTTCATTTCTGTTTGTCTCAAGAC

TTTTAAAAAATTTCCTCTTGAATTTCTTCTTTTGACCCAATAATTGTTTA

GGAGCATATTGTTTAGTTTCCACATATTTCTTAATTTTATATGATTTCTC

ATGTAATTGATTTCTAATTTTATATTGTGGTCAGAAAAGATACACGATGG

GTTTTCTTAAATTTGTTGAGACTTGTCTGTGGCCTAACATATGATCTATC

CTGGAGAATGTTACATGTGTACTTGAGAAGAATCTGTATTTTCCTACTGT

TCAGGGCACAATGTTCTGTATATGTCTGTTAGGTCCATTTGGTCTAAAAT

GTCATTCAAGTCCAATGTTTTCTTATGAATTTTTCTGTCTATTGCTTAAA

GTGGAATATTGAAATTGCCTGCTATTATTATGTTATAGGCTATGTTTCCC

TTCAGATCCCTTAATGTTTGCTTTATATATTTAGGTGCTCTGATTTGGGA

TGCTTATATACTTGTTATGTCCTCTTGATGAAATAACCTTTATCAATATA

TAATGATGTTCTTTGTCACTTTGAACAGATTTGACCTAAAGATTATTTTG

TCTGAAGTAAGTGTAACTACCCTGCTCTCTTTTTGTTCTCATGTACATGG

AGTATCTTTTTTCATCCCTTTACTTTCAGTCTATGCATGTCCTTTAAGGT

GAAATGAGCCACTTGTAGGCAGCACATATTTGGGTCTTGTTTTTTGTTGT

TGTCGTTAATCCACTCAACCACTCTATGCCTTTTGATTGGAGAGTTTAAT

CTATTTACATTCAAAATAATGATGGATGGGTAAGGACTTACTAGTGTCAT

TTTGTTCATTGTTTCCTGGTTGTCTTACAGATTCTTTGTTCCTTTCTTCC

TCTATTGCTGTCTTCCTTTGTGTTTTGATGGTTTTGTGTAGTAGTATACT

TTGGGTCTTTTGTTTTTATCATTCATGTATGTATTATAAGTTTGTGCTTT

GTGGATACTCCGAGGCTTACATAAAACATTTTATAAGCTGATAATAACTT

AAATTTGATTGTGTGCATATACTCAACACTTTGACTCTCCCTCCTCCCAC

ATTTTATGTTTCTAACATCACAACTTACTTTTTTTTTTAATTATACTTTA

AGTTCTAGGGTACATGTGCACAACTTGCAGGTTTGTTACATATCTATACA

TGTGCCGTGTTGGTATGCTCCACCCATTAACTTGTCATTTACATTAGGTA

TATCTCCCAATGCTATCCCTCCCCCGTCCCCTCACCCCACGACAGGCCCC

GGTGTGTGATGTTCCCCTTCCTGCGTCCAGGTGTTCTCATTGTTCAATTC

CCACCTATGAGTGAGAACATGCGGTGTTTGGTTTTCTGTCCTTGTGATAG

TTTGCTGAGAAAGATGATTTCCAGCTTCATCCATGTCCCTACAAAGGATG

TGAACTCATCCTTTTTTATGGTTGCATAGTATTCCATGGTGTATATGTGC

CACATTTTCTTAATCCAGTCTATCATTGATGGACATTTGGATTGGTTCCA

GGTCTTTGCTATTGTGAATAGTGCAGCAATAAACATACCTGTGCATGTAT

CTTTACAGCAGCAGGATTTATAATCCTTTGGGTATATACCCAGTAATGGG

ATAGCTGGGTCAAATGGTATTTCTAGTTCTAGATCCCTGAGGAATCGCCA

CACTGACTTCCACAATGGTTGAACTAGTTTACAGTCTCACTAACAGTGTA

AAGTGTTCCTATTTCTCCACATCCTCTCCAGCACCTGTTGTTTCCTGACT

TTTTAATGATTGCCATTCTAACTGGTGTGAGATGGTATCTCATTGTGGTT

TTGATTTGCGTTTCTCTGATGGCCAGTGATGATGAGCATTTTTTCATGTG

TCTTTTGGCTGCATAAATGTCTTCTTTTGAGAAGTGTCTGTTCATATCCT

TCGCCCACTTTTTGATGGGGTTGTTTGTTTTTTCTTGTAAGTTTGTTTGA

GTTCTTTGTAGATTCTGGATATTAGCCCTTTGTCAGATGAGTAGATTGCA

AAAATTTTCTCCCATTCTGTAGGCTGCCTGTTCACTCTGATGGTAGTTTC

TTTTGCTGTGCAGAAGCTCTTTAGTTTCATTAGATCCCATTTGTCCGTTT

TGGCTTTTGTTGCCATTGCTTTTGGTGTTTTAGACATGAAGTCCTTGCCC

ATGCCTACGTCCTGAATGGTATTGCCTAGGTTTTCTTCTAGGGTTTTTAT

GGTTTTAGGTCTAACATTAAGTCTTCAATCCATCTTGAATTAATTTTTGT

ATATGGTATAAGGAAGGGATCCAGTTTCAGCTCTCTACATATGGCTAGCC

AGTTTTCCCAGCACCATTTATTAAATAGGGAATCCTTTCCCCATTTCTTG

TTTTTGTCAGGTTTGTCAAAGATCAGATGGTTGTAGATGTGTGGTATTAT

TTCTGAGAGCTCTGTTCTGTTCCATTGGTGTATATCTCTGTTTTGGTACC

AGTACCATGCTGTTTTGGTTACCGTAGCCTTGTAGTATAGTTTGAAGTCA

GGTAGCGTGATGCCTCCAGCTTTGTTCTTTTGGCTCAGGATTGTCTTGGC

AATGTGGGCTCTTTTTTGGTTCCATATGAACTTTAAAGTAGTTTTTTCCA

ATTCTGTGAAGAAAGTCATTGGTAGCTTGATGGGGATGGCATTGAATCTA

TAAATTTCCTTGGGCAGTATGGCCATTTTCACGATATTGATTCTTCCTAT

CCATGAGCATGGAATGTTCTTCCATTTGTTTGTGTCCTCTTTTATTTCCT

TGAGCAGTGGTTTGTAGTTCTCCTTGAAGAGGTCCTTCACATCCCTTGTA

AGTTGTATTTGTAGGTATTTTATTCTCTTTGAAGCAATTGTGAATGAGAG

TTCACTCATGATTTGGCTCTCTGTTTGTCTGTTATTGGTGTATAAGAATG

CTTGTGATTTTTGCACATTGATTTTATATCCTGAGACTTTGCTGAAGTTG

CTTATCAGCTTAAGGAGATTTTGGGCTGAGACGATGGGGTTTTCTAAATA

TACAATCATGTCGTCTGCAAACAGGGACAATTTGACTTCCTCTTTTCCTA

ATTGAATACCCTTTATTTCTTTTTCCTGCCTGATCGCCCTGGCCAGAACT

TCCAACACTATGTTGAATAGGAGTGGTGAGAGAGGGCATCCCTGTCTTGT

GCCAGTTTTCAAAGGGAATGCTTCCAGTTTTGCCCATTCAGTATGATATT

GGCTGTGGGTTTGTCATAAATAGCTCTTACTATTTTGAGATACGTCCCAT

CAATACCTAATTTATCGAGAGTTTTTAGCATGAAGGGCTGTTGAATTTGG

TCAAGGGCCTTTTCTGCATCTATTGAGATAACCATGTGGTTTTTGTCGTT

GGTTCTGTTTATATGCTGGATTACATTTATTGATTTGCGTATGTTGAACC

AGCCTTGCATCCCAGGGATGAAGCCCACTTGATCATGGTGGATAAGCTTT

TTGATGTGCTGCTGGATTCGGTTTGCCAGTATTTTATTGAGGATTTTTGC

ATCGATGTTCATCAGGGATATTTGTCTAAAATTCTCTTTTTTTGTTGTGT

CTCTGCCAGGCTTTGGTATCAGGATGATGCTGGCCTCATAAAATGAGTTA

GGGAGGATTCCCTCTTTTTCTATTGATTGGAATAGTTTTAGAAGGAATGA

TACCAGTTCCTCTTTGCACCTCTGGTAGAATTCGGCTGTGAATCCGTCTG

GTCCTGGACTTTTTTTGGTTTGTAGGCTATTAATTATTGCCTCAATTTCA

GAGCCTGTTATTGGTCTATTCAGGGATTCAACTTCTTCCTGGTTTAGTCT

TGGGAAGGTGTATGTGTGCAGGAATTTATCCATTTCTTCTAGATTTTCTA

GTTTATTTGCGTAGAGGTGTTTATAGTATTCTCTGATGGTAGTTTGTATT

TCTGTGGTGTCGGTGGTGATATCCCCTTTATCATTTTTTATTGCATCTAT

TTGATTCTTCTCTCTTTTCTTCTTTATTAGTCTTACTAGCGGTCTATCAA

TTCTGTTGATCTTTTCAAAAAACTGGCTCCTGGATTCATTGATTTTTTTG

AAGGGTTTTTTGTGTCTGGATCTTGTAGGTATGCTTCATTGTTTCTTATT

ATTTTTTTCTTTTGTCTTCTCTGGCTGTGTATTTTCAAATAGGCTGTCTG

CCTACAAGATCCAGAAAATAGCCTCAAAAGGGTCAATCTAAGAGTTATTG

GCCTTAAACAGGAGGTAGAGAAAGAGATAGGGATAGAAAGTTGATACAAA

GGGACAATATCAGAGAACTTCAGAAACAGAGAAAGATACCAACATTCAAG

TACGACAAAGTTATAGAACACCAAGCAGAATTATCTCAGAGACTACCTCA

AGGCATGCAATAATCAAACTCCCACAGGTCAAGGATAAAGAAAGAATCCT

AAAAGCAGCAAGAGAAAGGAAACAAATAACATGCAGTGGAGCTTCAATAC

ATCTGGCAGCAGATTTTTCGGTGGAAATCTTAGGCCCCGGGCATATCCAG

AGATGCTGTCTGAGGGCCAGTCATTGGAGTCAAAAACCTTAGCAGTTTAC

CTCATGTTCTATTCTATTGTGGCTAAGCTAGCACTCACACCACAATATAA

AGTGCTCCCTGCTCTTCCTTGCCCTTTTAAAAGGCAGAGGATCCTCTCCC

TGTGGCCCTCACCACCATGAGGGTTCTGCTTGGCCTCCACTGGTGTTCAC

TTAAAGCCCAAGGGCTCTTCCATCAGCTTGTGGTGAATGCTGAGAGAACT

GGGACCCATATTTTAGGGCCTTGGGCTCCCCTCTGGCCCAAGGCAGGACC

AAAAATGCTGTCCAAGAACCTAGGCCAGGACTCAGAAATCCCAGAAGCCT

GCCTGCTTCTCTGCCTTTCTGTGGCTGAGCTGGTACCTAAGGGGCAAGAC

AAAGTCCCCTTTACTTTTCTGTCTACTTTTCTCAAACAGAAGGGGTCTTT

CACCATAACCACCACAGCTGGGAATTTGCTGGGTGACCTATGAAGCCAGC

ACATCTCAGAGGCCAAGTCCCACAGTGTACTCCCTGGGTATTGCAACTGG

TTATTCAACGTTCAAGGCCTCTTTAGTTAGTAGCTGATGAATCCTGATAG

GACTGAGTCCTTCCCTTTAAGGTAGCAGATTCCCTTTTGGCCCAGGGTGT

GTCTAGAAATGCTATCCAGGAACTAGGGCCTGGAATGGGGGCCTCATGAC

TCTGCCCATGCCCCATCCTACTGTGGCTGAGCTGGTATCCAAGATGCAAG

ACAAAGTCTTCTTTACTTTTCGCTCTCTTCTCCTTAACGAGAAGTAAGGA

GTCACTTCTGTTGCTGCAAGCTTCACTGCTGGGAGTAGGGGAGGTATGGT

GCAACCACTCCCTTAGCCATGCCAGCTGGTGTCTCCCTAGGTCATGTGGG

AGACCCTAATCCACTGGCTTCAATATATAGAGGGACGTTTCTAAATTATT

TATCTGTAATTTGATTAAGAAGATAGACACAGGCAATAGGATATGCCAAC

AGATTCTCTCTAACCTATAGTTATTTTAAGAAAGTTAGGGAAAGAGAGGT

CATTGATTAGTTTTGGCTTACTGAATTATTTGACCCTCCCATATCTTTTA

ATTTATGGATTTTATATAAGGCACAGATATTCTACTAGTAAACATGACAT

TAAAGATGTTTTATACAAATGAATGTGGTTGATACAAAGGCATTAAATAA

GAAACAAAGGAAATTCAGAGGACATTTGTTCGCCTGGAATAGAGATCATT

AGCATAAGCATAAGAGGAAAATAAGGAAGGAAATGGGAAAGTCTTGAGTC

CATTTTCAAATTATGAAAACTTGAAATGCAACAAACAAATAGTGGTTACT

TAAGAAGAAAGACTGAAGATGCTGGGCAAAGGTTAACTTTAGAAATGGCA

TTTTTATATCATTATAAAGAGAAAGTGGGGATAATGGAAATAATCTACAT

GTACAACTCATTTGACAGACATTTATTGAAGGTATTCCAAATGCCAAACA

CTAGTTTGTGCACTAAGGATGTTTTTAATTTTTACTTTTTACTTTATTTT

TATTTTCTAGCTTCTTCCGCTTTGCCAGAGAAGGATTTTTTTAAATGGAT

AAAATACTAACTTAGGCTAGCTTCTTTCAGAAACAGACCTTTAAACAATG

ATTAATGGTGAAAGTGGTTTGGTTGTAATGTGATCCCAGGAAGTACCAGT

AGGAGAGTGGGACAATGAGATAATAAATGAAAGAAGCCAATAATTAAGCC

AGTTATGGAACTAGTTATTGCTTTGGGCCACTGGGGTTTGATCCTGATGA

AGGAGCTCTGGGAGATAGTAAACAAACAAACAAACAAAAAACATGCCTCA

AAGTTGTCAACCACAAGGGGGTAAAGGCAAGGAACCAGGGCTAACTATTC

CAACTCTTATCCATCATTGGCTAAATGCTTCCTGGTACATAAACTTTCCA

GCACTTCTGGCCAGCGCACCTAGCAAGCTGAGGAAAATCTCTCAGGTTTG

CAGTAGGGCAAATGCTTGTACTAGGACACTGCTGGCATATACTTGAAGGA

TGAGTGCCAACGGCAGATAGATGGGCCCTGACAGCATCTTCTACAGATTC

TGTCCTTGCCTTTGAGGCAAGGACACTGTCTAGAGTCAGAGACAGAAATG

TAAAATGTAAAAATGTAAATCATAGTAGCACACAGTACAAGTTTTAAAAT

CTTAACAAAGTGTTATGGGAGAACCACGGTGAGAGAAAATGACTGCACAT

ATGAGAGCAGGGGAAGTCTTTATATGTGATGATGACATTTGAGTGGGTGT

TCCGTTATCAAAGATCTACTTAAAAGGGAAAAAGAGTAGAATTATTTTAA

ACCATGGATGTAACCAGGCGTGGCCACCCTATATGTGAATGTCCTGGGAT

AATTTTGTCCCCAGATGTCTCTCTTTTTGAGTGTCATTTTCATTTATGTA

TTCATAATATAAAGAAATTGAATAGAAAGAGGTAAACATTGGTGGCAAAA

ATCCCTTCCTACCCATAAGGCTCACAGAATAATGTTGTCTAAATGCCATA

ACATTTTTGGTAAGTTTTGGTTCTATGGGCTTTGGAAATTTTTTATGTAG

GAACTCCTCCAAGGTGATATAAGCATTCAGTGTATTTATCTAGATACCCT

AGTTAAAGCTACTTTAAGCAGGATCAAAGTCCTCTTTTAAACTTTTGTAT

TTGAAAGCTATTCCCATGTACAATTCCCATGATTCTTGTACAATTCCTAG

GAGACCTTTGTTTACATCAAAAGAGTTTGTCTCTCTCTATAAGTTAGAAC

TTGTACATTGTAGTAATGGAAAATCCATTTCAAAGTAGCTTAGACTTACT

CAGACAAAATGTATTGATCAGTGTCATTAAACAGTTGAAAGATAGACGAG

GCTTGATTCAGGAATTAAATAGCATGACCAGATTCTAGTTTTCCTTCTCC

ATTGCATGGCTCTGCTCCCCAGTATTTGTTCCGTTTAATTCCTCTTAATT

GTTCTAAGATGGATATCAGGATACTGCAGAACTACGTGCTTTCTCATCCA

TCTTAAACAAGAAGCAATGGGTTTTCTCTTTTAGAATCACGAACAACAAC

AACAACAACAAAAATCCTAGAGTTGTTTCATTGGTTCTGATTGACCTTAC

TTGGAACCATAAGTCCATCTTTGAATTAACCACTAAAGCCAGGGAGATGA

GTTAAAACTAAGTACACACCATCCACAGAGCAAAGAGAGGTCTATTGTGG

GAAAACTGCACAAATGGAAAAATAACAGGGGAAACACAGGAGAAGGAGGT

AGTTAGTTAATGTTCAATACACGCACCTTCTCAGGGACTTTTCTTCCATC

TGGCATCATCTTTGTCTTTTGACCATTTTCCCCAATCAATACCTATATTC

ATACTCAAGTTTTCCATATTTGGGAAAAAACTTCTCTTGACACCAAACTC

CCCTCTGACTTCCTTCTTATAATGATCTATGGTTGGATAGTCTACAATTT

ACTGTCCTTACTTTTTCATAGGTATTTTTTAATCTCCTACAGTAAGATTT

CTACCACTGCACTGAAATCATTTTGTGTATGGTCTTCAGGGAATTCCATG

TGGCCAAAGCCAATGGATACTTTTGGCATGGGCTTTGGAGGTCTAACTTC

TTGAATCCAAGTGTCGGCTCTAACAATCACTATCTATGTGACCTTAGACC

AGTTATTTAACGCTGTCTTTATTTCCTCATTTGTTAAATGAGGATAATTG

CAGCAATAGAAAAAGAACGTAAACAATTATACTTATAGGATTGTATGATG

ATTAAGTGATTAATGCAAGGAAAGAACAACACAGAGCTTCAGACAGTGTA

GATATTCAAATAAATGTTAGTTATGATTTTTATTATGTCTTGTGACATCT

ATGAGATTTGAAAGTGTTAATCACTCTTGTGTTTTTGAAAATCTTTCTTT

TCCATGTTCTATAAAAGTACACTTTCTTGATATTCCTCCTCCTTCTCTAG

TCTTTCATTCATCTTATTTGCTCTTTGTGTTTTATTTATTCTTTTTACGT

TGTATTTTCCAGGAGTCCATTCCCAGCCCGGTGTTGGGCTCAGTCTATGT

GCCATATAAATGAGAATGCACTTGCATTTGAGGCTTTGCTTTTTATTTCT

CTGTAGTGACGGCCAATCCCAGATTTCTGTCTCTCCCAAAATACACAGCT

ATGCTATAGATCCTTCAGTCCTCCTGACTAAAATGTTAGTTTTATGTGTG

TGTTCCTCACAACCTCCCAGTGAATAGGTCCAAAACTGAACTCATTATCT

TCTCCATGCTCACTCTGCATTCTTTGGATAGGCAGTGAATAGCACCACTA

TCTAACAGGCACCTAAACCAGAAGCCAGGTAATCTGTCTTAAACCCTTTT

GTTTTCCAAGTTCTTATCATAATGGCTGGTGTCTAACAGATGGTAAATAA

AATAGAGACTAGCTGGCTACATGGATGGATGTGTGGGGATGCACAGAGGG

ATAGATGGATTATTGAGTGAGTCTCTAGCCTATAAATATTTTTAATTAAC

TAATAAATTATGATAATGTACAGTTGAAGGTCAAGGGTGAAAAAGCATAC

CCTCAGTGGGATGCACACCCCAAGGAGCCATTTTAACTTGCTACATAACA

CACATATGACCACTTTTTTGCTGAAAGCCTTCTATTATTGAGCAAGCATT

CAAATCCCATGTTTGCCTGAAATAATGGTTCAGGTTATAAAAGTTTCCTT

CTTTTTCCAGGATTAAAATTATCTTCCTACATAATAGGAAGAACTGCTTT

ATCTTTTCCTAATATCTAGAGATGGCCTTTTAAAAATATAGACTGTTTTC

CCTATTGAAATGAACTGTAGGATGTACAAAATATTTACTGGCATGAATCA

AAAGAGCTTGCTATGTTTATGTGAAAACCACTAGGCATTCTAAAAAATAT

TGCTAGCATAGTAAAATGTTAGTAATTAAGACTAACGAAAGCGAAGGCAA

ATTGGAATCAGAGACTATTTTTAAGGAATGTCAACTGTATTATTTTCAAA

TACACATGGTACATAACAGTAGGATATGAGAAAAAGTCCCAAGTATGTGT

ACTAAAGTAGCCTGCTATGATAAGTTGAAAAAGGGTTTGTAATTGGAATA

TCCACAGAATATTTCAGAACACTTAAAGACATTTTCATTTACACTTTATA

CAGCTTTCTTATAAGAGCATTTACACCATTTATTTTATAAACCAAAGATT

AATTAGAAGACTAAACAATTACAAGGCCTCAACTACGAAAGCTGTTCCAC

TACCTAGTGGAACAACAACAATGAGACACACAAAACAATGGCGTTCAAAG

ATTAGAGAGAGACTTACGGTTAAACAGAGGTTGACATGTTAACTGAAGTT

GCAATATAATATGTCGACTAGTTTTGCAATACATAGCAAACACCCAAACA

GAAATAAACCTGATAAAAAAACAGTAGTCTATAATGTGTGCCACTTACTG

AGTTTTAATTATTCTGGGGACTATATTTTTGATTTCATGTTACAATCACT

AGTTTTGTGGGGTCTTTCTAGTCCTGATGCTTATTTACAAAATATCTGAA

GTATTTCTTTCTATGTATTTATTTTTGAGATGGAGTTTTGCTCTGTCACC

CAGGCTGGAGTGCAGCGGCATGATCTCGGCTCACTGCAACCTCTGCCTCC

CGGGTTCAAGTGATTCTCCTGCCTCAGCCTCCTGAGTAGCTGGGATTACA

GGCGTGTGCTAATTTTTGTATTTTTAGTAGAGACTGGGTTTCAGCATGTC

GGTCAGGCTGGTCTCGAACTCCTGACCTCATGATCCACCCGCTTTGGCCT

CCCAGAGACCTGGGATTACAGGCGTGAGCCACCGCACCTGGCCATCTCAA

GTATTTCTTTAACTTATAACTTCACATAACTTTGTGGAGGCAACAGGGTT

AATTAAAAAGGACTTTACTTACATAACAAAATAAGAAGCATAGTTTTATA

TTCCTGTGCCATATACATTTTGTTTGTCCATCTGTAGCCATTCTTTGACC

TTCTCTCCCTTGCTCTTTATTCTGGGAGGCTGACCTCTGTCATCATTGGG

CTCCCATGCCCTTTGGCTTCCAGTTGGTTTAGGCACCCAAGAGCCCTAGA

AGGAAATTGAAGACAGGAGGTAAAGTGAGGTCAAGATATTTATTCTCCTA

ATTCCCTCCCTTTGAGGTTGCCACAGGCTGACTATGTCCTTTGACAAAAG

GTTATTGCTCTTCTCAGGGTGGTTTCTCATTCCAATTCTCTGCTTTTGGC

CACTTTTCCCTCCCCTCATCCCTTGGGCCTAGATGTAGTAACAGCTCTAC

TGTTGCAAGGTTCTTGTATTATCTGTGATGGTTTCTTATACCCTGCTTAT

CTTGTGATTTGTTGCTTTGTAGATAAACCTCTCAGATTATCTAGGCAAGA

TCATAGAAGAACACGTATGTCCAGCTAAGATATTCCAGAAGACAGTAGGG

AAACATTGAAGGGTTTTATAAGAGGGAGTGCAGTGATCAGATTTATGTTA

GTTTTAATCTTTAATTGGGGTAGAATTTACATTCTAAAACAGAGATTTGG

GTCTGGGAGGATGATATAGAGCCTCTTATGGATGTGAGGGCAAAAAATGA

TAGAGGTTTGCAGTGCCAATAGAAAGAGGAAAGAAGTTATATATGAGAGA

AATTTGTAACTAATTATATGAGGTGTTGGGTGACGCTTAGGAAAGAATCT

AGAATGTCTATCAGGTTTCATGCTTAAGGGATAAAGTAGATGGCAGTTTT

ATTACTTGTTTTCTGTATTATTTTTATTTCATAAAACCAGCTTAGAGAAG

TTGCATAGAAAAAATAATGTAGTCCTGTTTATTTTAATATTTGAAAAGAA

CATATTTCAGAGTAGAATCTATATAGTACCTCCCTCTTGGACTTCCAATG

ATACCAGTGATAGCCTCAATATAAGCCAGTCTTACAAAATGCACCCAGCG

TGAATTCTTAGGTATTGTTAAAAGAAGTTGGCCAGGCGCGGTGGCTCACG

CCTGTAATCCCAGCACTTTGGGAGGCCGAGGCAGGTGGATCACCTGAGGT

CAGGAGTTTGAGACCAGCCCGGCCAACATGGTGAAACCCTGTCTCTATTA

AAAATACAAAAAAAAAAAATTAGCTGGGCATGATGGCACGTGCCTGTAGT

TCCTGCTACTCGGGAGGCTGAGACAGGAGGATAGCTTGAACCCGGGAGGC

GGAGGTTGCAGTGAGCGGAGATTGCACCATTGCACTCCAGCCTGGGTTAC

AAGAGCAAAACTCCATCAAAAAAAAAAAAGAAAGAAAAAGAAGAAGTTTC

TAATACACTTATCTTCCCTTGGGTTCACTCAGAAGACCCTTGGAAAAGGT

TTTAAGAGCAAGTGATTTATTTGGGGGGTAAATTAATCAGTAGAAGAGTG

GAAAAATGAGACAGGTGAGGCAAGGCAGCCAGTAAAGAGTGGTGCATTAT

CAAGCCAGCTGCTGTTGTGGGTCACTGGAGCTTTATCCCTTGGGAAACTC

TGGAACCCTTGTAAAATACATGCCTCAGAGTTATTTCCCCTAGCATCAAG

GGAGCTAGTGTAACAATATCCCAATTCCTACAATTAGTCATTATATACAG

GCTGCCTCTGGGAGCTGGAGGGGAGGCATCAGTTGCCTGGTATGTCTAGC

CTGTCACATGGATGGCAAAGCAAACTCCTGTGGCAACAGAAAGCCTTCAG

GCAATGAAATGCTGGCACTGGGAAATCAGGCTGATGGGTGCTGAAGTGGC

AAGGATGAGGGGATATGGATATTCTGCTGTAGTGCTTTTCTAACAGATGA

TTCATATTTGGTTCTAGGGATCAAGAATTGAGTTAAAATTTTATATATAT

GTTGATGTTCTATGTCACCTTCAGGAAAATAATTTAACAGAAACTAATAT

TTGCCATCAAAAAAGCAAAGAATCCTGTTGTTCATCATCCTAGCCATAAC

ACAATGAATAATTTTTTAAATAAGCAACATAAATGTGAGATAACGTTTGG

AAGTTACATTTAAAATGTCTCCTCCAGACTAGCATTTACTACTATATATT

TATTTTTCCTTTTATTCTAG

50044 BP

BLASTN TWO SEQUENCE: QUERY INTRON 50 VS INTRON 52. HIGHEST HOMOLOGY REGIONS (>80%) FASTED

PLOTS ANALYSIS


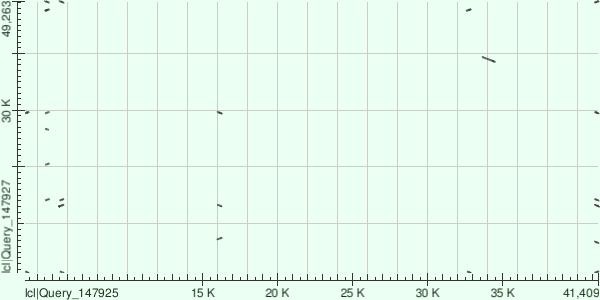


| **Sequence ID: Query_147927 (intron 50) Blastn Intron 52 Length: 50044Number of Matches: 24 REGIONS OF HOMOLOGY**  **In grey the TTTAAA-like motifs in the two sequences**  Range 1: 38506 to 39369[Graphics](https://www.ncbi.nlm.nih.gov/projects/sviewer/?RID=N0DNVFSK114&id=lcl|Query_147927&tracks=[key:sequence_track,name:Sequence,display_name:Sequence,id:STD1,category:Sequence,annots:Sequence,ShowLabel:true][key:gene_model_track,CDSProductFeats:false][key:alignment_track,name:other alignments,annots:NG Alignments|Refseq Alignments|Gnomon Alignments|Unnamed,shown:false]&v=38463:39412&appname=ncbiblast&link_loc=fromHSP)Next MatchPrevious Match   | **Alignment statistics for match #1** | | | | | | --- | --- | --- | --- | --- | | Score | Expect | Identities | Gaps | Strand | | 990 bits(536) | 0.0 | 759/868(87%) | 10/868(1%) | Plus/Minus |   Query 33654 CAAATTTACAAGaaaaaaaCAAACAACCCCATCAAAAAGTGGGGGAAGGATATAAACAGA 33713 |||| ||||||||||||| |||||||||||||||||||||||| ||||||||| |||||| Sbjct 39369 CAAACTTACAAGAAAAAA-CAAACAACCCCATCAAAAAGTGGGCGAAGGATATGAACAGA 39311 Query 33714 CTCTTCTCAAAAGAAGACATTTATGTGGCCAAAAGACACATGaaaaaaaGCTCATCATCA 33773 | ||||||||||||||||||||||| ||||||||||||||||||||| ||||||||||| Sbjct 39310 CACTTCTCAAAAGAAGACATTTATGCAGCCAAAAGACACATGAAAAAATGCTCATCATCA 39251 Query 33774 CTGGTCATTAGAGAAATGCAAATCAAAACCGCAATGAGATACCATCTCACATCAGTTAGA 33833 |||| ||| ||||||| ||||||||||||| |||||||||||||||||||| |||||||| Sbjct 39250 CTGGCCATCAGAGAAACGCAAATCAAAACCACAATGAGATACCATCTCACACCAGTTAGA 39191 Query 33834 AAGGCGATCATTAAAAAGTCAGGAAACAACAGATGCTGGAGAGGATGTGGAGAAATAGGA 33893 | ||| |||||||||||||||||||||||||| ||||||||||||||||||||||||||| Sbjct 39190 ATGGCAATCATTAAAAAGTCAGGAAACAACAGGTGCTGGAGAGGATGTGGAGAAATAGGA 39131 Query 33894 ACGCTTTTACACTGTTGGTGGGAGTGTGAATTACTTCAACCATTGTGGAAGACCGTGTGG 33953 || |||| |||||||| ||| || ||| || || ||||||||||||||||| | |||||| Sbjct 39130 ACACTTT-ACACTGTTAGTGAGACTGTAAACTAGTTCAACCATTGTGGAAGTCAGTGTGG 39072 Query 33954 CGATTCCTCAAGGATCTACAACCAGAAATACCATTTAACTCAGCCATCCCATTACTGGGT 34013 |||||||||| ||||||| ||| ||||||||||||| || |||| ||||||||||||||| Sbjct 39071 CGATTCCTCAGGGATCTAGAACTAGAAATACCATTTGACCCAGCTATCCCATTACTGGGT 39012 Query 34014 ATATACCCAAAGGATTATAAATCATTCTACTGTAAAGACACATGCACACGTACGTTTGTT 34073 ||||||||||||||||||||||| | || ||||||||| ||||||||| ||| |||| || Sbjct 39011 ATATACCCAAAGGATTATAAATCCTGCTGCTGTAAAGATACATGCACAGGTATGTTTATT 38952 Query 34074 GCAGCACTGTTTACAATAGCAAAGACTTGGAACCAACCCAAATGTCCATCAATAATAGAC 34133 || ||||| || |||||||||||||| ||||||||| |||||||||||||||| |||||| Sbjct 38951 GCTGCACTATTCACAATAGCAAAGACCTGGAACCAATCCAAATGTCCATCAATGATAGAC 38892 Query 34134 TGGATACAGAAAATGTGGCACATATACACCATAGAATACTATGCAGCCATAAAAAAGGAT 34193 ||||| ||||||||||||||||||||||||| |||||||||||| |||||||||||||| Sbjct 38891 TGGATTAAGAAAATGTGGCACATATACACCATGGAATACTATGCAACCATAAAAAAGGAT 38832 Query 34194 GCGTTCGTGTCCTTTGTAAGGACATGGATGAAGCTGGAAACCATCATTCTCAGCAAACTA 34253 | |||| ||||||||| ||||||||||||||||||||| |||| |||||||||||||| Sbjct 38831 GAGTTCACATCCTTTGTAGGGACATGGATGAAGCTGGAAATCATCTTTCTCAGCAAACTA 38772 Query 34254 ACACAGGAACAGAAAACCAAACACCGCATGTTCTCACTCATAAGTGGGAGTTGAACGATG 34313 |||| | |||||||||||||||||||||||||||||||||| |||||| |||||| ||| Sbjct 38771 TCACAAGGACAGAAAACCAAACACCGCATGTTCTCACTCATAGGTGGGAATTGAACAATG 38712 Query 34314  AGAacacatggagatgggggaggggaacatcacacaccag----t-t-gggggattgggg 34367 ||||||| |||| || |||||||||||||||||| | | | | ||| | | || Sbjct 38711 AGAACACCTGGAC-GCAGGAAGGGGAACATCACACACCGGGGCCTGTCGTGGGGT-GAGG 38654 Query 34368 ggcagagggagggaTAACGTTAGGAGAAATACCTAATGTAGATGAAGGGTTGATGGGTAC 34427 || | |||||||||| | || ||||| |||||||||||| |||| ||| |||||| Sbjct 38653 GGACGGGGGAGGGATAGCATTGGGAGATATACCTAATGTAAATGACAAGTTAATGGGTGG 38594 Query 34428 AGCAAACCACCATGGCACGTGTATACCTACGTAGCAAACCTGTACATTCTGCACATGTAT 34487 |||| |||| || ||||| |||||| || ||| |||||||| | || |||||||||| Sbjct 38593 AGCATACCAACACGGCACATGTATAGATATGTAACAAACCTGCAAGTTGTGCACATGTAC 38534 Query 34488  CCCAGAACTTAAAGTATAATT**T**AAAAAA 34515  || |||||||||||||||||| |||||| Sbjct 38533  CCTAGAACTTAAAGTATAATT**A**AAAAAA 38506 |
| --- | --- | --- | --- | --- | --- | --- | --- | --- | --- | --- | --- | --- | --- | --- | --- |
