# Supplementary figures and images for: The Genetic Landscape of Dystrophin Mutations in Italy: A Nationwide Study
Source: Front Genet. 2020 Mar 3;11:131. doi: 10.3389/fgene.2020.00131 (PMC7063120; doi:10.3389/fgene.2020.00131)

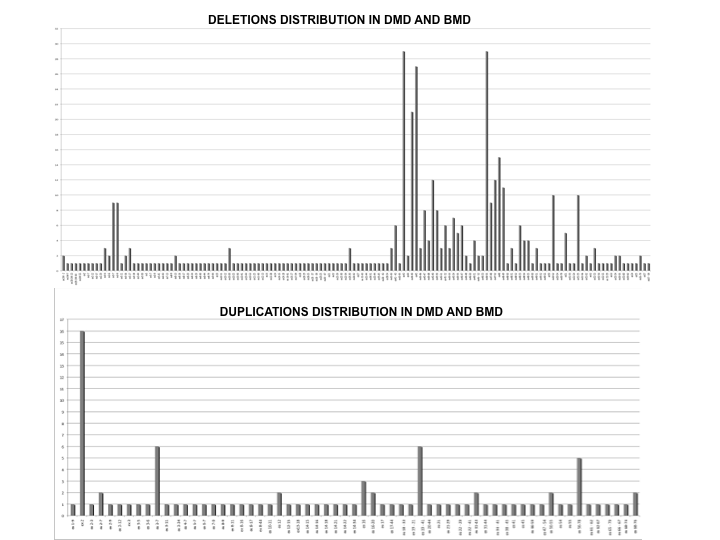

Supplement: Supplementary Figure 1 — Exonic distribution of deletions and duplications in DMD and BMD patients DMDs have 57% of deletions and 11% of duplications. The most common single exon deletion in DMD is exon 45 while the most common multiple exon deletion is 45-52. Deletions were non-randomly distributed, occurring in the two known hot spots at the 5’ and 3’ end of the gene and very heterogeneous. [file Image_1.tif]

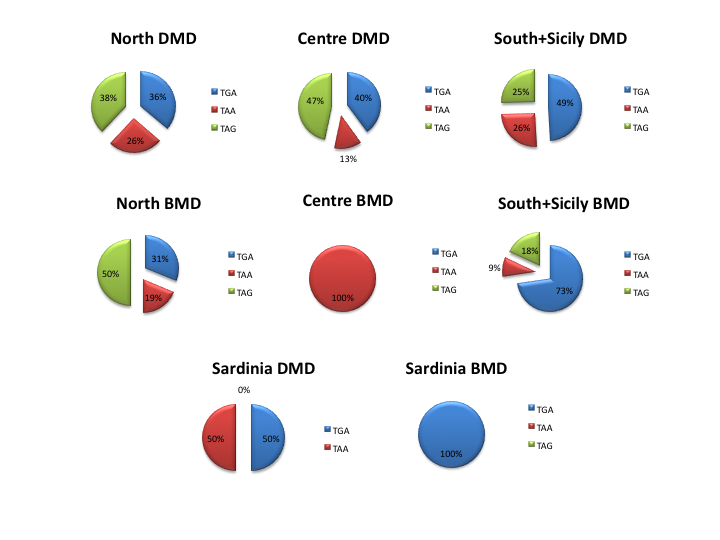

Supplement: Supplementary Figure 2 — Geographical distribution of nonsense codons in DMD and BMD patients The three codons are almost equally represented in the national mutation spectrum; regional distribution showed that in Northern and Central regions this trend is maintained with TAG slightly more frequent than the others (38% North, 47% Center). In Southern and Sicily the TGA codon is the more frequently occurring nonsense mutation (49%). Sardinian DMD show only TGA and TAA nonsense codons, while in BMD only the TGA codon (100%) was found. [file Image_2.tif]

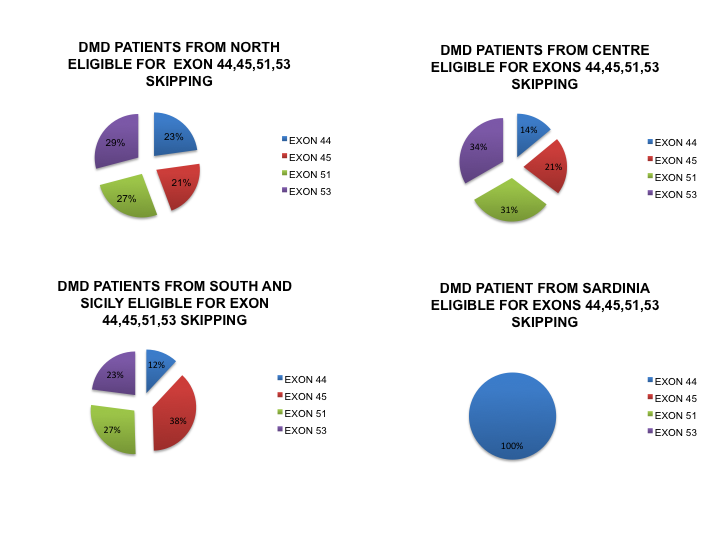

Supplement: Supplementary Figure 3 — Geographical distribution of skippable deletions in DMD patients Northern and Central regions show very similar percentages of skippability, being the exon 53 the more frequently skippable exon, South and Sicily have a different pattern being exon 45 the predominant skippable exon. In Sardinian patients, only exon 44 skipping is applicable in DMD patients. [file Image_3.tif]
